# Thioparib inhibits homologous recombination repair, activates the type I IFN response, and overcomes olaparib resistance

**DOI:** 10.15252/emmm.202216235

**Published:** 2023-01-18

**Authors:** Li‐Min Wang, Pingyuan Wang, Xiao‐Min Chen, Hui Yang, Shan‐Shan Song, Zilan Song, Li Jia, Hua‐Dong Chen, Xu‐Bin Bao, Ne Guo, Xia‐Juan Huan, Yong Xi, Yan‐Yan Shen, Xin‐Ying Yang, Yi Su, Yi‐Ming Sun, Ying‐Lei Gao, Yi Chen, Jian Ding, Jing‐Yu Lang, Ze‐Hong Miao, Ao Zhang, Jin‐Xue He

**Affiliations:** ^1^ State Key Laboratory of Drug Research, Cancer Research Center, Shanghai Institute of Materia Medica Chinese Academy of Sciences Shanghai China; ^2^ University of Chinese Academy of Sciences Beijing China; ^3^ Pharm‐X Center, School of Pharmacy Shanghai Jiao Tong University Shanghai China; ^4^ Institute of Evolution and Marine Biodiversity Ocean University of China Qingdao China; ^5^ The CAS Key Laboratory of Tissue Microenvironment and Tumor, Shanghai Institute of Nutrition and Health University of Chinese Academy of Sciences, Chinese Academy of Sciences Shanghai China

**Keywords:** homologous recombination repair, olaparib‐resistant, PARP inhibitor, PARP7, type I interferons, Cancer, DNA Replication, Recombination & Repair

## Abstract

Poly‐ADP‐ribose polymerase (PARP) inhibitors (PARPi) have shown great promise for treating BRCA‐deficient tumors. However, over 40% of BRCA‐deficient patients fail to respond to PARPi. Here, we report that thioparib, a next‐generation PARPi with high affinity against multiple PARPs, including PARP1, PARP2, and PARP7, displays high antitumor activities against PARPi‐sensitive and ‐resistant cells with homologous recombination (HR) deficiency both *in vitro* and *in vivo*. Thioparib treatment elicited PARP1‐dependent DNA damage and replication stress, causing S‐phase arrest and apoptosis. Conversely, thioparib strongly inhibited HR‐mediated DNA repair while increasing RAD51 foci formation. Notably, the on‐target inhibition of PARP7 by thioparib‐activated STING/TBK1‐dependent phosphorylation of STAT1, triggered a strong induction of type I interferons (IFNs), and resulted in tumor growth retardation in an immunocompetent mouse model. However, the inhibitory effect of thioparib on tumor growth was more pronounced in PARP1 knockout mice, suggesting that a specific PARP7 inhibitor, rather than a pan inhibitor such as thioparib, would be more relevant for clinical applications. Finally, genome‐scale CRISPR screening identified *PARP1* and *MCRS1* as genes capable of modulating thioparib sensitivity. Taken together, thioparib, a next‐generation PARPi acting on both DNA damage response and antitumor immunity, serves as a therapeutic potential for treating hyperactive HR tumors, including those resistant to earlier‐generation PARPi.

## Graphical abstract

PARP inhibitors (PARPi) resistance is ubiquitous in the clinic. A newly discovered pan‐PARP inhibitor, thioparib, is highly effective against olaparib‐resistant cancer models, which suggests that therapeutic vulnerabilities still exist in PARPi‐resistant tumors.

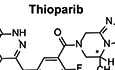

## Introduction

Homologous recombination deficiency (HRD), arising from germline mutations in *BRCA1* or *BRCA2*, provides unique opportunities for targeted therapy (Bryant *et al*, 2005; Farmer *et al*, 2005). To date, four poly‐ADP‐ribose polymerase (PARP) inhibitors (PARPi), including olaparib, niraparib, rucaparib, and talazoparib, have received approval from the FDA for treating BRCA‐mutated ovarian, breast, pancreatic, and prostate cancer (Wang *et al*, 2016b; Antonarakis *et al*, 2020). The use of PARPi for frontline maintenance offers substantial clinical benefits in patients who respond to platinum‐based therapy, including those with BRCA wild‐type tumors (Li *et al*, 2020; Dias *et al*, 2021).

Although PARPi hold great promise for treating BRCA‐deficient tumors, the clinical trial objective response rates rarely exceed 60%, and even fewer patients achieve complete remission (Li *et al*, 2020; Zhang *et al*, 2021). Moreover, many patients who initially respond to PARPi therapy eventually develop acquired resistance. Additionally, resistance to PARPi often correlates with platinum resistance, which remains the backbone therapy for most BRCA1/2‐mutated tumors. Several mechanisms of PARPi resistance have been identified, including the following: (i) Secondary somatic mutations restoring BRCA1/2 are the most common mechanism of PARPi resistance in patients; (ii) in a fraction of cases, loss of 53BP1 also causes PARPi resistance by partial restoration of homologous recombination (HR); and (iii) mutations in PARP1 that abrogate trapping have been reported to confer PARPi resistance (Li *et al*, 2020; Dias *et al*, 2021). Combination therapies with PARPi are being explored in an effort to enhance efficacy and overcome PARPi resistance, but more studies are needed to investigate the feasibility in the clinic (Curtin & Szabo, 2020; Dias *et al*, 2021). Currently, DNA polymerase theta (Polθ) inhibitors have been used to target PARPi‐resistant tumors mediated by the loss of 53BP1. However, tumor cells with other mechanisms of PARPi resistance, such as BRCA1 or BRCA2 restoration, remain resistant to Polθ inhibitors (Zatreanu *et al*, 2021; Zhou *et al*, 2021). Therefore, effective methods for overcoming PARPi resistance are still lacking.

The concept of applying PARPi to treat HR‐deficient cancers has largely been based on the fact that PARP inhibition impairs base excision repair (BER) and single‐strand repair (SSR), therefore leading to the accumulation of DNA double‐strand breaks (DSBs), which cannot be fixed by defective HR repair (Bryant *et al*, 2005; Farmer *et al*, 2005). Interestingly, several studies have reported that PARP1 knockdown or inhibition impairs HR (Jelinic & Levine, 2014; Chen *et al*, 2019b). One study demonstrated that PARP1 itself is required for HR by modulating the nucleosome density at damage sites. Particularly, the PARPi olaparib and PJ34, which impair PAR‐mediated chromatin relaxation response to DNA damage, have strong inhibitory effects on HR repair (Chen *et al*, 2019b). These findings suggest that PARPi may be a useful therapeutic strategy not only for treating BRCA‐deficient tumors but also for treating a wider range of hyperactive HR tumors, even HR‐proficient cancers.

It has been noticed that some non‐BRCA1/2 mutated, but HR‐deficient tumors, termed as BRCAness tumors, are sensitive to PARPi treatment as well (Lord & Ashworth, 2016; Hoppe *et al*, 2018). Because BRCA deficiency is rare in hematological malignancies, the clinical applications of PARPi have not been translated to blood cancers as an effective therapy (Zhao & So, 2016; Machado *et al*, 2020). Previous studies have reported that TCF3‐HLF‐positive leukemic cells, AML1‐ETO‐ and PML‐RARα‐transformed leukemic cells, and LMO2‐positive DLBCLs or T‐cell acute lymphoblastic leukemia (ALL) cells all exhibit high sensitivity to PARP inhibition (Esposito *et al*, 2015; Piao *et al*, 2017; Parvin *et al*, 2019). Therefore, PARPi may be a potential therapeutic approach for treating hematological cancers defective in HR.

Emerging evidence indicates extensive crosstalk between the DNA damage response (DDR) and the immune system. Several studies have reported that PARPi treatment produces cytosolic dsDNA, which induces type I interferons (IFNs) and related immune responses via the cGAS‐STING pathway, dependent or independent of BRCA status (Ding *et al*, 2018; Pantelidou *et al*, 2019; Shen *et al*, 2019). Moreover, the activation of cGAS‐STING signaling closely depends on PARPi‐mediated PARP1 trapping and the presence of PARP1 protein (Kim *et al*, 2020). A recent study indicated that the PARP7 inhibitor RBN‐2397, but not the PARP1 inhibitor olaparib, induced type I IFN signaling, as demonstrated by STAT1 phosphorylation (Gozgit *et al*, 2021). RBN‐2397 could inhibit the mono‐ADP‐ribosylation of TBK1, leading to the phosphorylation of TBK1 and subsequent activation of IFN signaling (Yamada *et al*, 2016; Gozgit *et al*, 2021). The role of PARP1 and/or PARP7 in PARPi‐induced type I IFN signaling needs to be further clarified. Nevertheless, these studies suggest that the activation of type I IFN signaling is a critical molecular mechanism underlying the therapeutic effects of PARPi.

In addition to catalytic inhibition, PARPi exert their cytotoxicity by preventing PARP1 auto‐PARylation and trapping it on damaged DNA. Compared with other PARPi, talazoparib is a 3‐ to 8‐fold more potent PARP1 inhibitor *in vitro* (Shen *et al*, 2013), but it exhibits 100‐fold greater potency at trapping PARP‐DNA complexes compared with olaparib and rucaparib in cells (Murai *et al*, 2014). In the “PARP1‐trapping” model, trapping is defined as chromatin‐bound PARP1 in methyl methanesulfonate (MMS)‐treated cells (Helleday, 2011; Ström & Helleday, 2012). By measuring changes in PARP1‐DNA binding (i.e., changes in fluorescence anisotropy values, termed ΔFA values) in the presence of NAD^+^, we showed that the differences in the cytotoxicity of PARPi originate from increased PARP1‐DNA binding due to the auto‐PARylation inhibition of PARP1 on DNA (Chen *et al*, 2019a). These results provide evidence that PARP‐DNA binding activity is an important factor for PARPi‐induced cytotoxicity. Furthermore, recent studies suggest that PARP trapping is primarily due to the inhibition of the activity of PARP1 and that the basis for the high potency of talazoparib lies in its extensive interactions with the active sites of PARP1 (Rudolph *et al*, 2022). Thus, we propose that novel PARPi should be screened and assessed by both PARP enzymatic and PARP1‐DNA binding activities.

At present, next‐generation PARPi are constantly being developed with the aim of identifying more specific PARP1 inhibitors (such as AZD5305) with fewer side effects (Johannes *et al*, 2021); however, it is unknown whether they can target tumors that have acquired resistance to earlier‐generation PARPi. Here, we report the discovery of new PARPi using screening based on both the inhibition of PARP enzymatic activity and changes in PARP1‐DNA binding activity, and identified thioparib as a high‐potent pan‐PARP inhibitor. Thioparib appeared to induce biochemical and cytotoxicity profiles similar to those of earlier‐generation PARPi, such as olaparib and talazoparib. However, thioparib could kill HR‐deficient, PARPi‐resistant tumor cells at much lower concentrations than other PARPi. Moreover, thioparib had strong inhibitory effects on HR‐mediated DNA repair, accompanied by the activation of type I IFN signaling. Our results demonstrate that thioparib can be used for treating HR‐deficient tumors, even after they acquire resistance to earlier‐generation PARPi.

## Results

### Thioparib is identified as a novel PARP inhibitor

**Figure 1:**
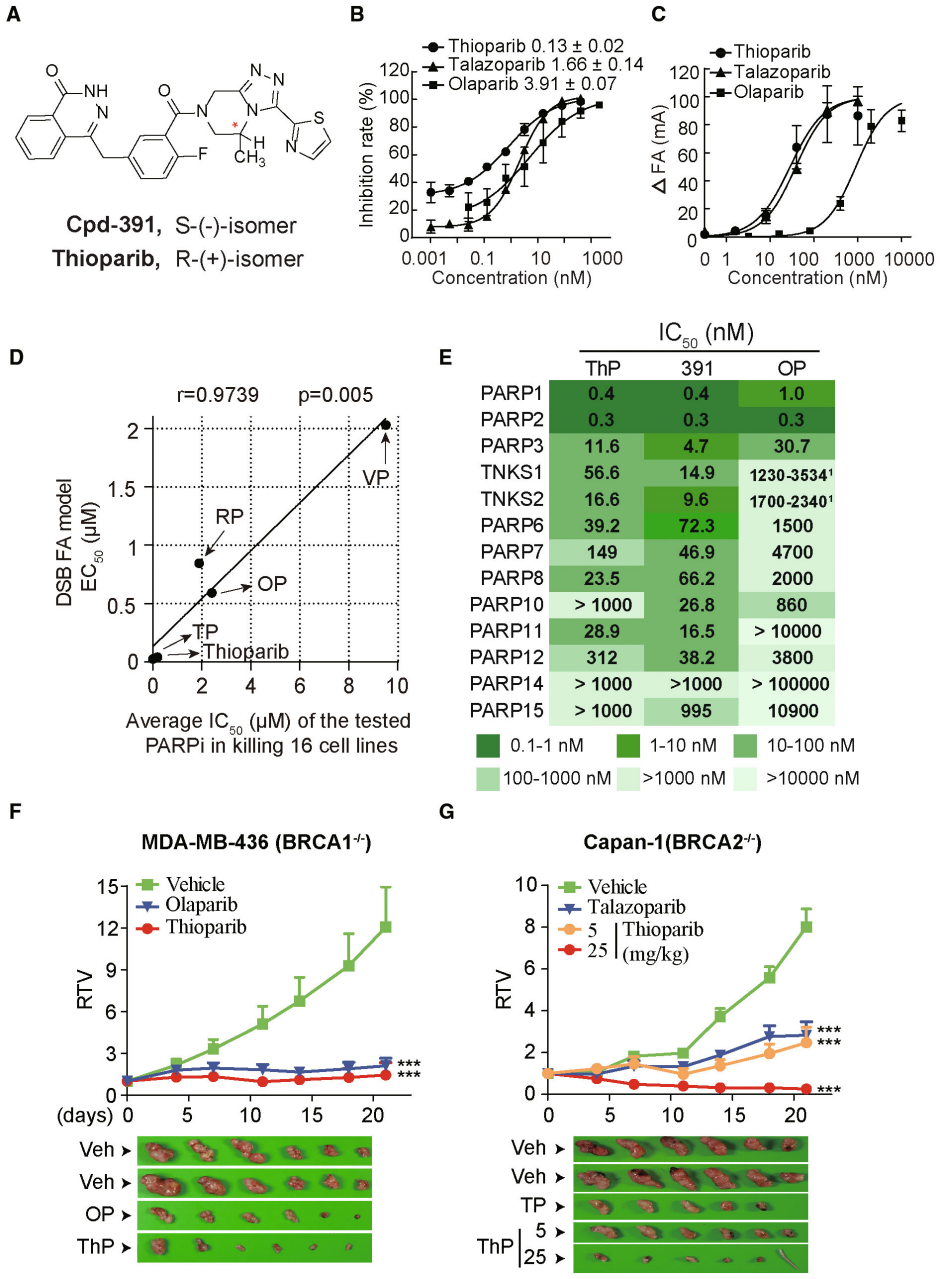
Thioparib is a potent pan‐PARP inhibitor and kills BRCA‐deficient tumors *in vivo* AStructures of thioparib and its enantiomer Cpd‐391.BThe concentration‐effect relationships of PARP1 inhibition by thioparib, talazoparib, and olaparib, as assayed by histone‐based ELISA. Data are obtained from three biological replicates and depicted as mean ± SEM.CConcentration‐dependent increase in PARP1‐DNA binding by three PARP inhibitors using DSB FA assays. FA was measured 60 min after adding NAD^+^, and the ΔFA values represent the changes in PARP1‐DNA binding. Data from four biological replicates are presented as mean ± SEM.DCorrelation between cytotoxicity and EC_50_, as measured by the DSB FA model. The cytotoxicity data are shown in Appendix Table S2. The Pearson test was performed to calculate the correlation.EIC_50_ values of the 13 isoforms of PARPs, as detected by biotinylated NAD^+^‐based luminescence assays. Numbers with superscripts are data from the reference (Antolin *et al*, 2020).F, GEffects of thioparib on BRCA1‐deficient MDA‐MB‐436 (F) and BRCA2‐deficient Capan‐1 (G) xenografts. Mice‐bearing MDA‐MB‐436 tumors were dosed orally with 10 mg/kg thioparib, 100 mg/kg olaparib, or vehicle (Veh) once daily for 3 weeks; and mice‐bearing Capan‐1 tumors were dosed orally with 5 or 25 mg/kg thioparib, 0.3 mg/kg talazoparib, or vehicle (Veh) once daily for 3 weeks (*n* = 6). Data are shown as mean ± SEM. Statistical analysis was performed by two‐way ANOVA. ***P < 0.0001. Representative images of xenograft tumors are shown, and mice tails represent complete regression of tumors. Data information: ThP, thioparib; OP, olaparib; TP, talazoparib; RP, rucaparib; VP, veliparib; 391, Cpd‐391.

PARPi are usually screened by determining their ability to inhibit the catalytic activity of PARP1 and PARP2. However, most PARPi induce different levels of cytotoxicity despite comparable inhibitory potency against PARP1/2 enzymatic activity (Murai *et al*, 2012). Notably, we previously reported that the cytotoxicity of PARPi is highly correlated with their abilities in inhibiting the dissociation of PARP1 from the PARP1‐DNA complex in the FA models (Chen *et al*, 2019a). Therefore, we used FA‐based methods to identify new PARPi that effectively inhibits PARP1‐DNA dissociation at nanomolar doses. To this end, we performed a two‐step screening strategy, comprising a histone‐based ELISA assay for the primary hit finding, followed by further assessment of the hit compounds of interest using the DSB FA model. Our screen led to the identification of thioparib as a potential PARP inhibitor, with an IC_50_ of 0.13 nM; the structure is shown in Fig 1A (Zhang *et al*, 2015). We observed an approximately 2‐fold difference in potency between thioparib and its enantiomer, ent‐thioparib (Cpd‐391; PARP1 IC_50_ = 0.26 nM) (Appendix Table S1). In a side‐by‐side comparison, we found thioparib to be more potent than the clinically approved PARPi talazoparib and olaparib (Fig 1B; Appendix Table S1). Most PARPi also inhibit the homologous enzyme PARP2. Indeed, PARP2 was catalytically inhibited by thioparib, with an average IC_50_ of 0.006 nM (Appendix Table S1).

When auto‐PARylation is inhibited, the PARP1 protein is unable to dissociate from the DNA strand. Therefore, PARPi are capable of enhancing PARP1‐DNA binding in the presence of NAD^+^ as earlier described (Hopkins *et al*, 2015). In FA models, the association reaction was measured by mixing DNA oligomer, PARP1 enzyme, and substate NAD^+^, and the changes in PARP1‐DNA binding or the dissociation of PARP1 from DNA (i.e., ΔFA values) represent the ability of each PARPi to inhibit auto‐modification of PARP1 on DNA. The kinetic characteristics of thioparib in terms of inhibiting PARP1‐DNA dissociation were assessed using DSB FA assays. Under the conditions of this experiment, thioparib, talazoparib, and olaparib showed IC_50_ values of 25.05, 41.03, and 592.65 nM, respectively (Fig 1C; Appendix Table S1). These data indicated that thioparib exhibited the strongest capacity to inhibit PARP1‐DNA dissociation, which was approximately 2‐ and 10‐fold greater than talazoparib and olaparib, respectively. Consistent with the previous study (Chen *et al*, 2019a), our analyses revealed a highly significant correlation (*r* = 0.9739; *P* = 0.005) between the EC_50_ values of five PARPi (thioparib, talazoparib, olaparib, rucaparib, and veliparib) in the DSB FA model and their average cytotoxic IC_50_ values in 16 HR‐deficient cell lines (Fig 1D; Appendix Table S2).

PARP1, PARP2, PARP5a/TNKS1, and PARP5b/TNKS2 are poly‐ADP‐ribosyl transferases (polyPARPs); PARP3, PARP4, PARP6‐8, PARP10‐12, and PARP14‐16 are mono‐ADP‐ribosyl transferases (monoPARPs); and PARP9 and PARP13 are inactive ADP‐ribose polymerases (Bütepage *et al*, 2015; Zhu *et al*, 2021). The selectivity and polypharmacology of thioparib and its enantiomer Cpd‐391 within 13 PARP isoforms were characterized *in vitro* using biotinylated NAD^+^‐based assays. As shown in Fig 1E, thioparib and Cpd‐391 showed similar inhibitory effects on PARP1 and PARP2 activities, with IC_50_ of 0.4 and 0.3 nM, respectively. Moreover, the two compounds were equally potent against most of the PARP isoforms, with an IC_50_ of 4.7–312 nM for PARP3, TNKS1, TNKS2, PARP6, PARP7, PARP8, PARP11, and PARP12. Thus, it can be said that thioparib is a pan‐PARP inhibitor that targets multiple PARPs.

**Figure EV1:**
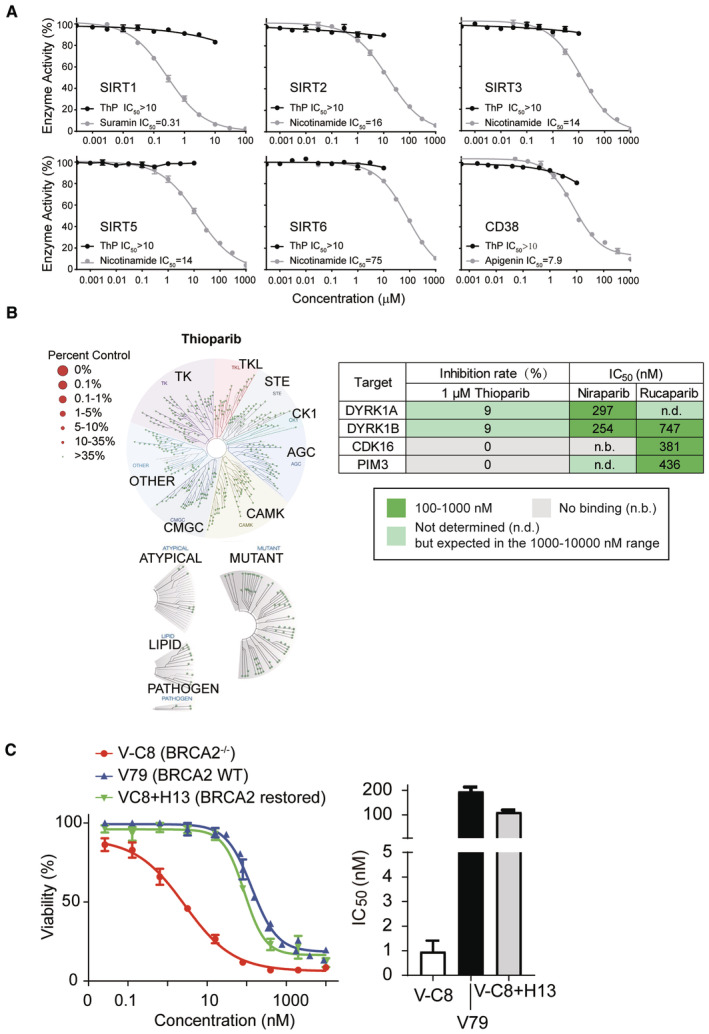
Effects of thioparib on NAD^+^‐related or kinase enzymes Concentration‐response curves of thioparib against NAD^+^‐related enzymes SIRT1, SIRT2, SIRT3, SIRT5, SIRT6, and CD38 using *in vitro* enzymatic assays. Suramin, Nicotinamide, and Apigenin are used as the positive controls. Data from two technical replicates are shown as mean ± SEM.Kinase selectivity of 1 μM thioparib in the DiscoveRx KINOMEScan screening platform. Left panel, TREEspot interaction maps for thioparib in 468 kinase targets. Right panel, a table summarizing the percentage of enzyme inhibition by 1 μM thioparib, and the IC_50_ values of niraparib and rucaparib for the off‐targets DYRK1B, CDK16, PIM3, and DYRK1A from published data.Thioparib sensitivity of V79 (BRCA2 wild‐type), V‐C8 (BRCA2 null), and V‐C8 + H13 cells (with BRCA2 restored) in SRB assay. Data from three independent experiments are presented as mean ± SEM.

In addition to PARPs, NAD^+^ is a substrate of multiple enzymes, including sirtuins and cyclic ADP (cADP) ribose synthases. We also assessed the effects of thioparib on SIRT1, SIRT2, SIRT3, SIRT5, SIRT6, and CD38. Although thioparib profoundly inhibited most of the PARP isoforms, it had no significant effect on these sirtuins and CD38 at concentrations of up to 10 μM (Fig EV1A), indicating that it displayed high selectivity for other NAD^+^‐related target classes (> 1,000‐fold). PARPi have also been found to inhibit the activity of some kinases, including DYRK1s, CDK16, and PIM3 (Antolin *et al*, 2020). To identify the potential off‐target activities, we performed a high‐throughput KINOMEscan *in vitro* binding assay and found that 1 μM thioparib showed no significant inhibitory or stimulatory activity against any of these kinases (Fig EV1B). Together, these data demonstrated that thioparib exhibited a high‐potent and broad spectrum of inhibitory activities against PARPs but did not inhibit the other tested enzymes.

### Thioparib effectively kills HR‐deficient tumors

As a result of DNA repair defects, HR‐deficient cancer cells are extremely sensitive to PARPi through the mechanism of synthetic lethality. Therefore, we next tested the inhibitory effects of thioparib in a panel of tumor cell lines harboring *BRCA1* ^−/−^, *BRCA2* ^−/−^, *PTEN* ^*−/−*^, or *EWS‐FLI1* gene fusion. Almost all of the tumor models, either *BRCA1/2* deficient (UWB1.289, HCC1937, Capan‐1) or *PTEN* deficient (U251, PC3), were profoundly sensitive to thioparib. It was also notable that compared with other PARPi, thioparib was more potent in these HR‐deficient tumor cells *in vitro*. The average IC_50_ value for thioparib was 0.96 nM, which is 50‐, 340‐, and 4,200‐fold more potent than that of Cpd‐391, talazoparib, and olaparib, respectively (Appendix Table S3). HR‐proficient cells were relatively resistant to thioparib, as indicated by the high IC_50_ values of 196 and 111 nM in wild‐type V79 and BRCA2‐restored V‐C8 + H13 cells, respectively (Fig EV1C). These results demonstrate that thioparib displayed selectivity against HR‐deficient cells.

To confirm and strengthen the *in vitro* results, we next evaluated the antitumor activity of thioparib *in vivo*. Animals bearing MDA‐MB‐436 (*BRCA1*‐deficient) xenografts were orally administered thioparib at a dose of 10 mg/kg for 21 days. Thioparib‐treated tumors were significantly smaller than those from vehicle‐treated mice after 11 days of treatment and beyond (Fig 1F). At the end of the experiment, thioparib at 10 mg and olaparib at 100 mg led to similar effects, with inhibition rates of 88 and 82%, respectively.

The antitumor effect of thioparib on the growth of *BRCA2* deficient tumors was also examined *in vivo*. Capan‐1 is a human pancreatic cancer cell line that harbors a mutant *BRCA2* with a frameshift mutation (6174 delT) (McCabe *et al*, 2005). Previous data revealed that olaparib at 75 mg/kg only resulted in 27% tumor growth inhibition (TGI) in Capan‐1 xenograft tumors (Sun *et al*, 2018). Therefore, the most potent PARP inhibitor talazoparib was used as a positive control in this model. Oral administration of thioparib at 25 mg/kg for 21 days significantly inhibited the growth of Capan‐1 xenografts in mice, and 6/6 mice achieved a partial or complete response with an inhibition rate of 97% (Fig 1G). At the lower dose of 5 mg/kg, thioparib caused similar growth inhibition as that with 0.3 mg/kg talazoparib, with an inhibition rate of 69 and 65%, respectively. Exposure of mice to thioparib ranging from 5 to 25 mg/kg was well tolerated. These data suggest that thioparib strongly suppressed the growth of both *BRCA1*‐ and *BRCA2*‐deficient tumors.

### Thioparib overcomes initial and acquired olaparib resistance

**Figure 2:**
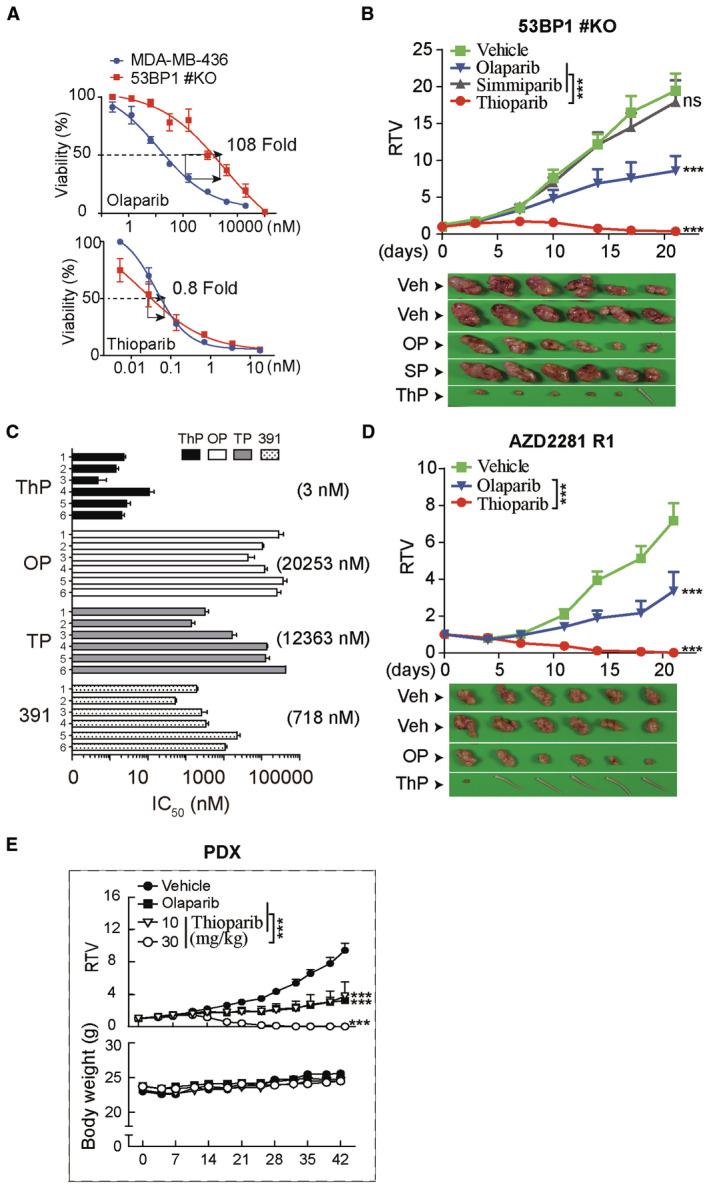
Thioparib overcomes multiple PARPi resistance mechanisms Thioparib and olaparib sensitivity of WT and 53BP1#KO (53BP1^−/−^) MDA‐MB‐436 cells. Cells were treated with thioparib or olaparib for 7 d and measured by CCK8 assay. Data from three independent experiments are presented as mean ± SEM.Efficacy of thioparib in the PARPi‐resistant CDX model MDA‐MB‐436 53BP1#KO. Mice‐bearing tumors (*n* = 6) were dosed with 10 mg/kg thioparib, 100 mg/kg olaparib, 10 mg/kg simmiparib, or vehicle (Veh) for 3 weeks. Data are depicted as mean ± SEM. ***P < 0.0001, ns, *P* = 0.7706.The IC_50_ values of thioparib in six acquired PARPi‐resistant cell lines. Numbers 1–6 represent the PARPi‐resistant Capan‐1/OP, Capan‐1/TP, MDA‐MB‐436/OP, MDA‐MB‐436/TP, U251/OP, and U251/TP cells, respectively. Cells were treated with the indicated PARP inhibitors for 7 days and then subjected to SRB assay. The IC_50_ values are expressed as the mean ± SD from three separate experiments.Efficacy of thioparib in the PARPi‐resistant model MDA‐MB‐436#AZD2281‐R1. Nude mice‐bearing MDA‐MB‐436#AZD2281‐R1 tumors (*n* = 6) were orally dosed with 40 mg/kg thioparib and 100 mg/kg olaparib for 3 weeks. Mice tails show complete regression of tumors. Data are shown as mean ± SEM. ***P < 0.0001.Efficacy of thioparib in the PDX model BR‐05‐0028 (BRCA1‐deficient). Mice‐bearing BR‐05‐0028 tumors derived from a patient with breast cancer (*n* = 6) were treated with 10 or 30 mg/kg thioparib or 100 mg/kg olaparib for 6 weeks. Data are depicted as mean ± SEM. ***P < 0.0001. Data information: Statistical analysis was performed by two‐way ANOVA. ThP, thioparib; OP, olaparib; TP, talazoparib; 391, Cpd‐391; SP, simmiparib.

Although several PARPi have been approved for treating multiple *BRCA*‐mutated advanced cancers, resistance remains a major challenge in this field. Loss of 53BP1 partially restores HR function in *BRCA1*‐deficient cells and confers resistance to PARP inhibition (Curtin & Szabo, 2020; Li *et al*, 2020; Dias *et al*, 2021). Genetic analysis found that *TP53BP1*‐loss was observed in 20% of PARPi‐resistant gBRCA1 patient‐derived xenografts (PDXs) (Cruz *et al*, 2018). We previously showed that compared with the parental BRCA‐deficient MDA‐MB‐436 cells, 53BP1/BRCA1 dual deficiency (namely MDA‐MB‐436 53BP1#KO) cells displayed significantly reduced sensitivity to various PARPi, including olaparib and simmiparib (Yang *et al*, 2017). Strikingly, we found that MDA‐MB‐436 53BP1#KO cell lines remained sensitive to thioparib but not to olaparib (Fig 2A). Consistently, thioparib monotherapy significantly reduced the growth of tumors and resulted in a 98% inhibition rate on day 21 in the MDA‐MB‐436 53BP1#KO xenograft model (Fig 2B). These results suggest that thioparib can achieve great efficacy in PARPi resistance tumors mediated by the loss of 53BP1.

**Figure EV2:**
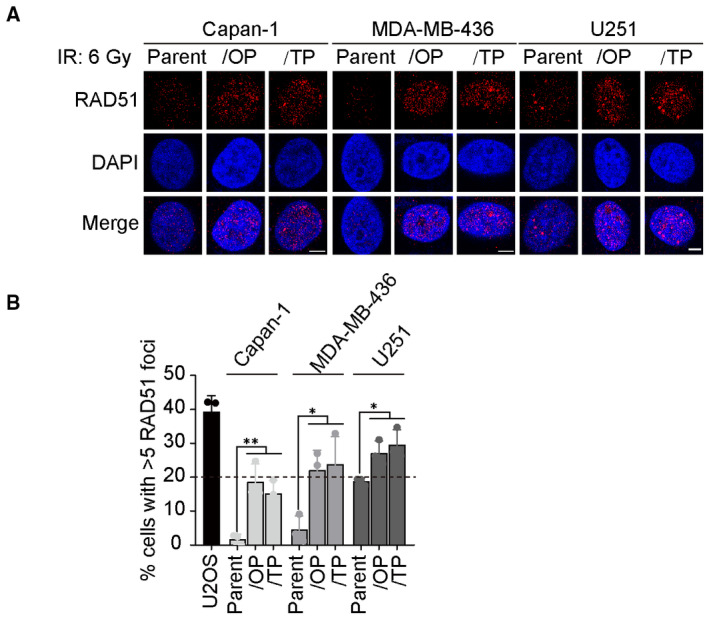
Restoration of RAD51 foci formation in a panel of cell lines with acquired PARPi resistance Immunofluorescence staining of RAD51 foci induced by 6 Gy irradiation in PARPi‐resistant clones Capan‐1/OP, Capan‐1/TP, MDA‐MB‐436/OP, MDA‐MB‐436/TP, U251/OP, U251/TP, and their parental cells. Representative pictures are shown. Scale bar: 5 μm.Quantification of RAD51‐positive cells with ≥5 foci. HR‐proficient U2OS cells were used as the positive control (data from Fig 5C). Data represent the mean ± SD. Data from three independent experiments were analyzed by one‐way ANOVA. **P = 0.0032, *P* = 0.0093, *P = 0.0247, *P* = 0.0170, *P* = 0.0433, *P* = 0.0153 (from left to right).

Another well‐known mechanism of PARPi resistance in *BRCA1/2*‐deficient tumors is the somatic reversion of *BRCA1/2* genes (Li *et al*, 2020; Dias *et al*, 2021). Previous studies have revealed that PARPi‐resistant cells with the reversion of *BRCA2* were not only resistant to the existing PARPi but also to Polθ inhibitors (Zatreanu *et al*, 2021; Zhou *et al*, 2021). To evaluate whether thioparib could overcome acquired PARPi resistance caused by different mechanisms, we established a panel of PARPi‐resistant cell lines (MDA‐MB‐436/OP and MDA‐MB‐436/TP cells with the reversion of *BRCA1*; Capan‐1/OP and Capan‐1/TP with the reversion of *BRCA2*; U251/OP and U251/TP with *53BP1* loss) through step‐wise dose escalation of olaparib (OP) or talazoparib (TP) for more than 1 year (Wang *et al*, 2018; Chen *et al*, 2020a). All these PARPi‐resistant cell lines had acquired the ability to form RAD51 foci, a hallmark for HR‐mediated DSB repair, in response to irradiation as compared to parental cells (Fig EV2A and B). The average IC_50_ value for thioparib was 3 nM in the six PARPi‐resistant cells we analyzed, which was 200‐, 3,600‐, and 6,000‐fold more potent than Cpd‐391, talazoparib, and olaparib, respectively (Fig 2C; Appendix Table S4). These data suggest that these cell lines remain sensitive to thioparib *in vitro*, although they were highly resistant to other PARPi, including olaparib and talazoparib. We then tested the *in vivo* potential of thioparib as a single agent to eliminate cancer cells refractory to olaparib using an olaparib‐resistant MDA‐MB‐436 xenograft model (named MDA‐MB‐436 #AZD2281‐R1). This olaparib‐resistant model was established by inoculating 5 × 10^6^ MDA‐MB‐436/OP cells s.c. in nude mice. Strikingly, thioparib at 40 mg/kg once a day revealed complete regression in five of the six mice examined, with an inhibition rate of 99%, showing greater efficacy than olaparib, which exhibited an inhibition rate of only 53% (Fig 2D), with a significant difference between the two groups (*P* < 0.001). These data show that thioparib is even effective in some tumors with acquired resistance to olaparib.

Some *BRCA*‐deficient patients may show intrinsic resistance and an insufficient response to PARPi (Li *et al*, 2020; Dias *et al*, 2021). Therefore, we used a PDX model BR‐05‐0028, generated from a *BRCA1*‐deficient human breast cancer, to further evaluate the effect of thioparib. Nude mice‐bearing tumors of the BR‐05‐0028 PDX model were treated with the indicated drugs, and tumor growth was monitored every 3 days. Five of the six mice showed complete tumor regression after treatment of BR‐05‐0028 with 30 mg/kg thioparib, with an inhibition rate of 99% (Fig 2E). By contrast, we only observed a small degree of tumor growth inhibition (~ 60%) following olaparib treatment at 100 mg/kg, with a significant difference observed between the two groups (*P* < 0.001). Taken together, these data show that thioparib may overcome multiple mechanisms of resistance to olaparib.

### Thioparib targets HRD‐associated hematological malignancies

**Figure 3:**
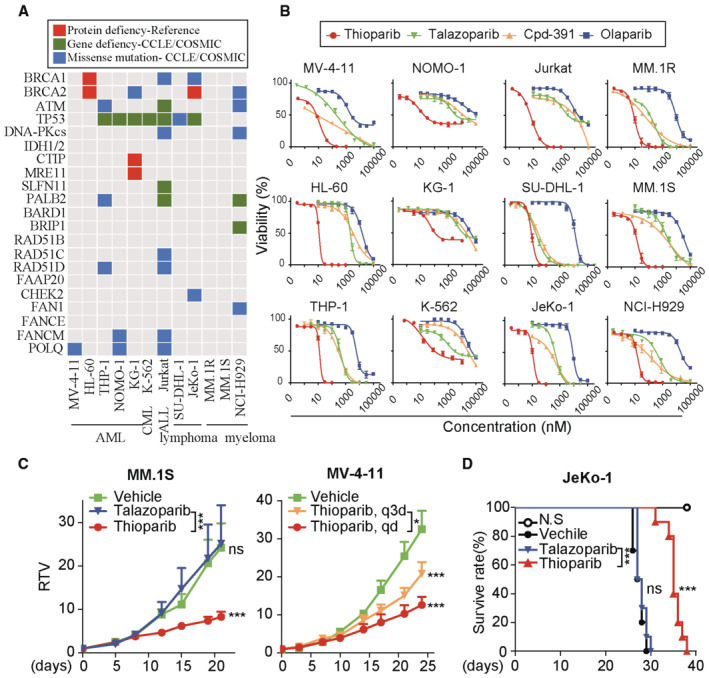
Thioparib targets HR‐deficient hematologic cancer cells *in vitro* and *in vivo* The chart indicates alterations of genes involved in the HR pathway in a panel of malignant hematologic cell lines.Concentration‐dependent effects of thioparib on the viability of malignant hematologic cells. Cells were treated with thioparib or other PARP inhibitors for 72 h and then subjected to CCK8 assay. Data from three independent experiments were presented as mean ± SEM.Effects of thioparib on HR‐proficient MM1.S (left) and POLQ‐deficient MV‐4‐11 (right) xenografts (*n* = 4). Mice‐bearing tumors were dosed orally with 0.3 mg/kg talazoparib, 10 mg/kg thioparib daily (qd), or 30 mg/kg thioparib once every 3 days (q3d). Data shown represent the mean ± SEM. Statistical analysis was performed by two‐way ANOVA. Left panel: *P* = 0.0007, *P* = 0.9741, *P* = 0.0005; right panel: *P* = 0.0296, *P* = 0.0003, *P* < 0.0001 (top to bottom).Kaplan–Meier survival curve of M‐NSG mice transplanted with JeKo‐1 cells treated with 10 mg/kg thioparib, 0.3 mg/kg talazoparib, or vehicle (*n* = 10). Data information: Statistical analysis was performed by log‐rank (Mantel–Cox) test. ***P < 0.0001, ns, *P* = 0.3526.

Germline or somatic mutations in HR‐associated genes have been identified in various hematological malignancies (HMs), particularly in acute myelocytic leukemia (AML) (Zhao & So, 2016; Machado *et al*, 2020). Several studies have indicated that changes in HR repair gene expression may render hematological cancers sensitive to PARPi (Esposito *et al*, 2015; Piao *et al*, 2017; Parvin *et al*, 2019). We therefore screened hematological cancer‐derived cell lines of various genetic and phenotypic backgrounds for sensitivity to thioparib; these included five AML (MV‐4‐11, HL‐60, THP‐1, NOMO‐1, and KG‐1), one chronic myelogenous leukemia (CML) (K‐562), one acute lymphoblastic leukemia (ALL) (Jurkat), two lymphomas (SU‐DHL‐1 and JeKo‐1), and three myeloma (MM.1R, MM.1 S, and NCI‐H929) cell lines. In addition to *BRCA1* and *BRCA2* genes, HRD‐associated gene mutations were defined as pathogenic lesions in the following genes: ATM, IDH1/2, CtIP, MRE11, SLFN11, PALB2, BARD1, BRIP1, RAD51B, RAD51C, RAD51D, FAAP20, CHEK2, FAN1, FANCE, FANCM, and POLQ (Wang *et al*, 2016a; 2017; Lok *et al*, 2017; Sulkowski *et al*, 2017). As shown in Fig 3A, 75% of the tested cell lines had a mutation in at least one of the HRD‐associated genes (Xiao *et al*, 2008; Gaymes *et al*, 2013; Faraoni *et al*, 2015). Notably, most of the cell lines (10/12) were profoundly sensitive to thioparib, with IC_50_ values ranging from 6.38 nM to 19.33 nM (Fig 3B). By contrast, the BCR‐ABL1‐positive KG‐1 and K562 cells were relatively resistant, with IC_50_ values of 79.71 nM and 119 nM, respectively. The average IC_50_ value for thioparib in the 12 hematological cell lines was 26 nM, which was 220‐, 80‐, and 990‐fold more potent than that of Cpd‐391, talazoparib, and olaparib, respectively (Appendix Table S5).

To further explore the *in vivo* antitumor effects of thioparib in blood cancers, we used xenografts of the HR‐proficient MM.1 S cell line, the POLQ mutant MV‐4‐11 cell line, and the BRCA1/2‐deficient JeKo‐1 cell line (Xiao *et al*, 2008), all of which have different genetic backgrounds and high sensitivity to thioparib. As a result, oral administration of thioparib at 10 mg/kg led to 65.7% tumor growth inhibition compared with the vehicle control in the MM1.S xenograft model. In the same study, talazoparib (0.3 mg/kg), the most potent PARP inhibitor, resulted in no significant inhibition of tumor growth (Fig 3C; left panel). Similarly, thioparib as a single agent significantly suppressed the growth of MV‐4‐11 xenograft tumors, with a maximum 61.26% inhibition of tumor growth (Fig 3C; right panel). It was noticed that once‐a‐day (QD) dosing might be more effective than a once‐three‐day (Q3D) dosing regimen. Additionally, thioparib treatment significantly prolonged the overall survival of mice‐bearing JeKo‐1 xenograft tumors compared with the vehicle group, with median survival times of 35 and 27.5 days for thioparib‐ and vehicle‐treated mice (*P* < 0.001, *n* = 10), respectively (Fig 3D). Talazoparib at 0.3 mg/kg revealed no significant effect on these models.

### Thioparib induces PARP1‐dependent DNA damage, S‐phase cell‐cycle arrest, and apoptosis

**Figure 4:**
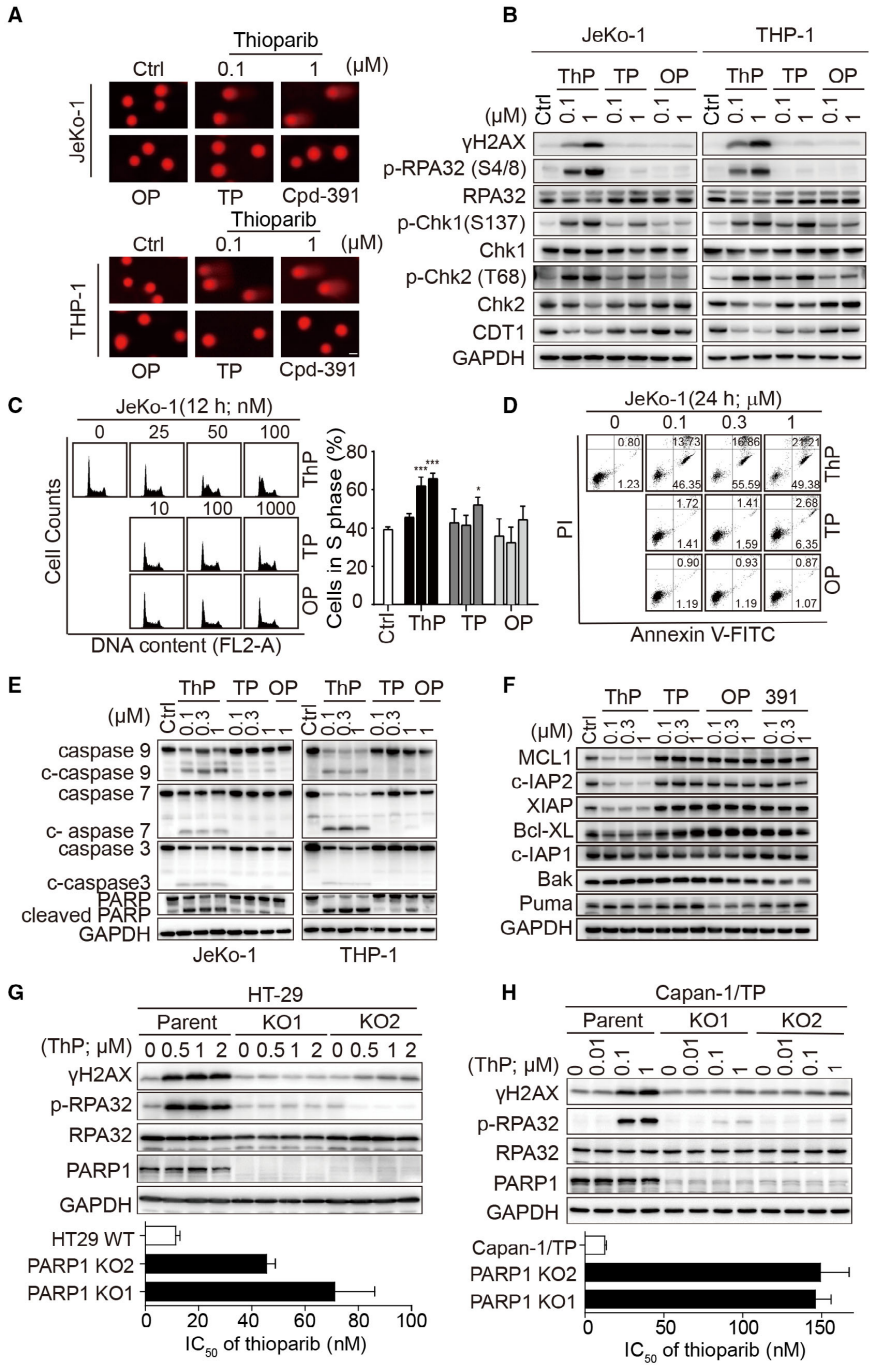
Thioparib treatment led to PARP1‐dependent DNA damage and induced S‐phase arrest and apoptosis ADNA damage as determined by the alkaline comet assay. JeKo‐1 and THP‐1 cells were treated with the indicated drugs for 12 h before being harvested for the comet assay. Representative photographs of the comet assay are shown; Scale bar: 25 μm.BWestern blotting of γH2AX and phosphor‐RPA32 in cells exposed to thioparib, talazoparib, or olaparib for 12 h.C, DEffects of thioparib on cell cycle and apoptosis. Cell‐cycle arrest (C) or apoptosis (D) induced by thioparib, talazoparib, and olaparib in JeKo‐1 cells were analyzed by PI staining‐based or Annexin V‐FITC‐based flow cytometry. Data from three independent experiments are expressed as the mean ± SD. Statistical analysis was performed by two‐way ANOVA. ***P < 0.0001, *P = 0.0131.E, FThioparib caused changes in apoptosis‐related proteins in JeKo‐1 or THP‐1 cells, as determined by western blotting. Cells were exposed to thioparib, talazoparib, olaparib, or Cpd‐391 for 24 h and then subjected to western blotting.G, HDepletion of PARP1 in HT‐29 and Capan‐1/TP cells resulted in a decrease in DNA damage and thioparib resistance. The upper panel shows the changes in γH2AX and phospho‐RPA32 in HT‐29 PARP1 knockout (KO) clones (G) or Capan‐1/TP PARP1 KO clones (H) and their parental cells after exposure to thioparib for 24 h. The lower panel shows the IC_50_ values of thioparib in these PARP1 KO clones and parental cells. Cells were treated with thioparib for 5 or 7 d and the IC_50_ values were determined by SRB assay from three separate experiments. Data information: Data are presented as the mean ± SD. ThP, thioparib; OP, olaparib; TP, talazoparib; 391, Cpd‐391. Source data are available online for this figure.

We next studied the mechanism of cell killing by thioparib. Previous studies have demonstrated that PARPi exert antitumor effects mainly through the blockade of BER repair, which leads to the generation of massive DSBs in HR‐deficient cells (Bryant *et al*, 2005; Farmer *et al*, 2005). Therefore, we used a comet assay to analyze DNA damage in JeKo‐1 (BRCA1/2 mutant) and THP‐1 (RAD51D mutant) cells in the presence or absence of thioparib. The amount of DNA damage was significantly higher when cells were treated with thioparib versus DMSO, while no significant change was observed in cells treated with either olaparib or talazoparib (Fig 4A; Appendix Fig S1A). Consistent with this result, increased levels of γH2AX and the DNA checkpoint markers p‐Chk1 and p‐Chk2 were observed in thioparib‐treated cells (Fig 4B). By comparison, only a slight increase of γH2AX was observed with talazoparib treatment, although elevated levels of p‐Chk1 and p‐Chk2 were noted. Similarly, the levels of replication stress marker, phosphorylation of RPA32 (S4/S8), were strongly upregulated upon thioparib treatment but not by other PARPi. CDT1 is an essential initiation factor for DNA replication, which is rapidly degraded in response to DNA damage (Kanellou *et al*, 2020; Misra *et al*, 2020). Indeed, the expression of CDT1 was strikingly reduced in response to thioparib (Fig 4B). The BRCA1‐deficient MDA‐MB‐436 cells were very sensitive to most of the PARPi, and a similar DNA damage response was observed after thioparib and other PARPi treatment (Appendix Fig S1B). Increased levels of γH2AX and p‐RPA32 were also observed after thioparib treatment in the colon cancer HT‐29 (POLQ mutant) cells and PARP‐resistant Capan‐1/TP cells (Appendix Fig S1C and D).

Previous studies have shown that PARPi are effective in activating the cell‐cycle checkpoint, resulting in typical G2/M arrest and caspase‐dependent apoptosis in sensitive cells (He *et al*, 2017; Yuan *et al*, 2017). Therefore, we performed flow cytometry analysis to examine the cell‐cycle effects following thioparib treatment in JeKo‐1 and THP‐1 cells. Surprisingly, most of the thioparib‐treated cells were arrested in S phase as early as 12 h after treatment (Fig 4C; Appendix Fig S1E). Moreover, severe S‐phase accumulation occurred in cells exposed to as low as 50 nM thioparib. By contrast, the S‐phase population only showed a slight accumulation at a higher dose (1 μM) talazoparib, whereas olaparib had no pronounced effect. Consistently, thioparib treatment markedly increased the percentage of apoptotic cells (60–72%) in JeKo‐1 and THP‐1 cells after 24 h (Fig 4D; Appendix Fig S1F). However, talazoparib or olaparib treatment induced much less apoptosis than thioparib in both JeKo‐1 and THP‐1 cells. To characterize the apoptotic cell signaling pathway induced by thioparib, activation of the apoptosis executioner was examined by western blotting. The protein levels of cleaved caspase‐3, caspase‐7, caspase‐9, and cleaved PARP1 were concentration‐dependently increased in cells with thioparib treatment but not significantly changed with talazoparib or olaparib treatment (Fig 4E). Furthermore, the antiapoptotic proteins MCL‐1, c‐IAP2, XIAP, c‐IAP1, and Bcl‐XL were markedly decreased in a concentration‐ and time‐dependent manner in cells following thioparib treatment, whereas the expression of the proapoptotic Bak and Puma proteins were not significantly changed (Fig 4F; Appendix Fig S1G). Interestingly, the kinetics of MCL‐1 and c‐IAP2 protein downregulation were somewhat in line with the activation of caspase‐3. By contrast, treatment with other PARPi did not significantly affect the antiapoptotic nor proapoptotic proteins in these cell lines. Together, these results suggest that thioparib treatment elicited replication stress/DNA damage, cell‐cycle arrest, and cell death in our model.

To investigate whether the effect of thioparib on DNA damage was due to PARP1 inhibition, we knocked out PARP1 in HT‐29 and Capan‐1/TP cells and examined replication stress/DNA damage makers. Our results showed that PARP1 depletion significantly impaired the effect of thioparib, as expected. As shown in Appendix Table S6, PARP1 knockout (#KO1) by CRISPR/Cas9 rendered HT‐29 cells resistant to thioparib, as well as other PARPi, including Cpd‐391, talazoparib, and olaparib. Similar results were also obtained with an additional *PARP1* KO clone (#KO2) obtained using CRISPR/Cas9 targeting different sequences of the gene. Additionally, relative to the PARPi‐resistant Capan‐1/TP cells, the Capan‐1/TP PARP1^−/−^ variants displayed up to 12‐fold reduced sensitivity to thioparib. Most importantly, the treatment of PARP1‐depleted cells with thioparib did not increase the levels of γH2AX and p‐RPA32 (Fig 4G and H). Overall, these results demonstrate that the observed sensitivity of HR‐deficient and/or PARPi‐resistant cell line models to thioparib is potentially due to strong induction of DNA damage, S‐phase cell‐cycle arrest, and apoptosis upon PARP1 inhibition with thioparib treatment.

### Thioparib represses HR repair activity but induces excessive RAD51 foci

**Figure 5:**
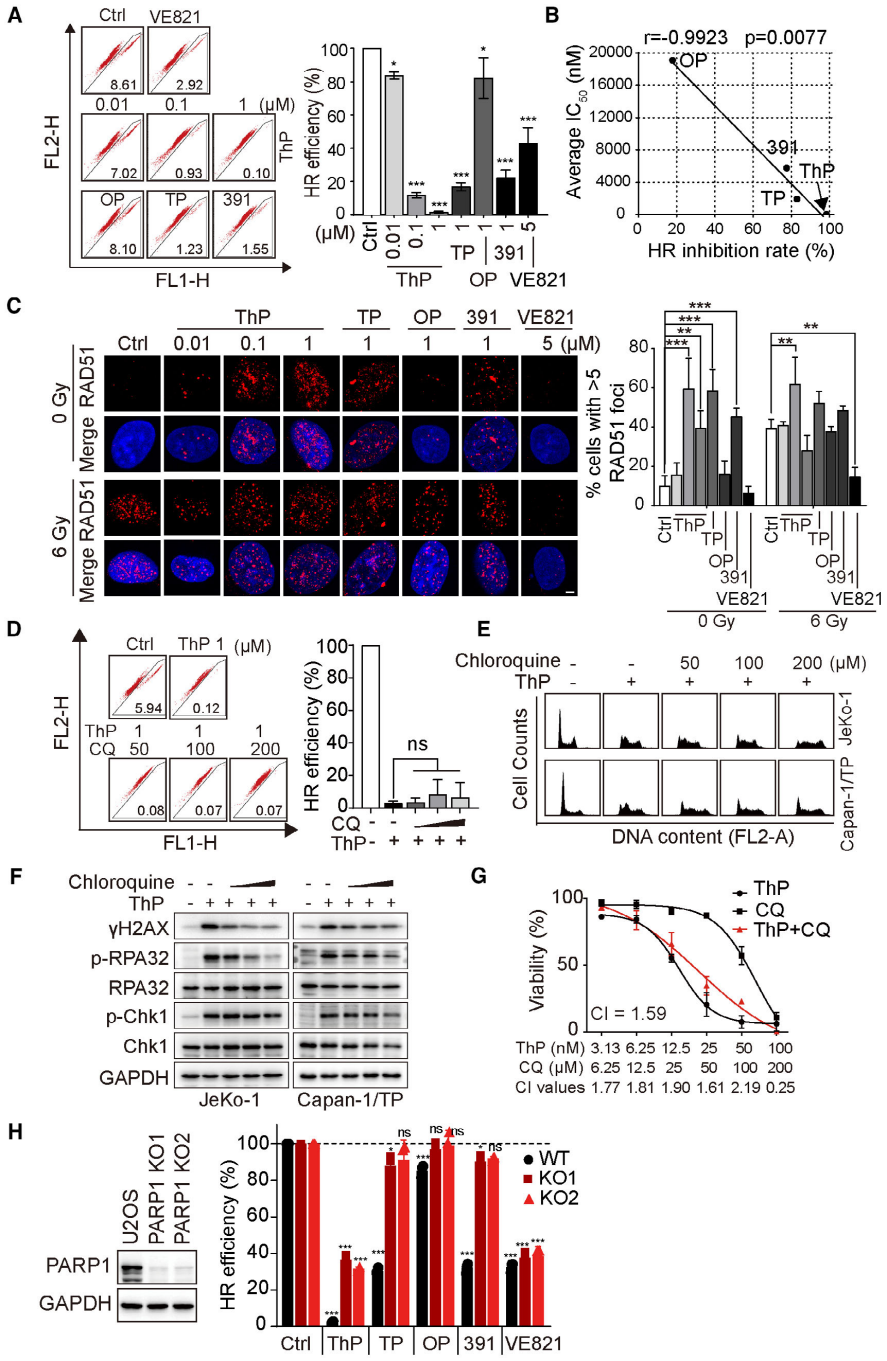
Thioparib treatment led to aberrant homologous recombination (HR) repair AHR repair assays in U2OS‐DR‐GFP cells treated with increasing concentrations of thioparib. Cells were incubated with the indicated drugs at the time of I‐SceI introduction for 48 h. GFP‐positive cells were analyzed after I‐SceI transfection for 72 h by flow cytometry. The ATR inhibitor VE‐821 was used as a positive control. Three independent experiments were performed and data are expressed as mean ± SD. Statistical analysis was performed by one‐way ANOVA, and all groups were compared with the control. ***: *P* < 0.0001, *: *P* = 0.0205, *P* = 0.0101 (from left to right).BCorrelation between the cytotoxicity of PARPi in 12 hematologic cancer cell lines (Appendix Table S5) and HR repair inhibition rates. The Pearson test was performed to calculate the correlation.CRepresentative images of RAD51 foci in U2OS‐DR‐GFP cells with or without radiation. Graphs (right panel) show the quantification of cells with ≥ 5 RAD51 foci in vehicle‐ and drug‐treated cells. Scale bar: 5 μm. Data are obtained from three biological replicates and presented as mean ± SD. Statistical analysis was performed by one‐way ANOVA. Left to right: *P* < 0.0001, *P* = 0.0033, *P* < 0.0001, *P* = 0.0006, *P* = 0.0031, *P* = 0.0015.DHR repair assays in U2OS‐DR‐GFP cells treated with thioparib with or without chloroquine. Cells were pretreated with chloroquine for 4 h, followed by thioparib treatment for 48 h. GFP‐positive cells were analyzed by flow cytometry 72 h later. Data are expressed as the mean ± SD from three independent experiments. Statistical analysis was performed by one‐way ANOVA. ns: *P* > 0.9999, *P* = 0.9763, *P* = 0.9972.E, FWestern blotting of γH2AX, phospho‐RPA32, and phospho‐Chk1 (E) or PI staining‐based flow cytometry analysis of the cell cycle (F) in cells exposed to thioparib with or without chloroquine. Cells were pretreated with chloroquine for 4 h followed by thioparib treatment (100 nM in JeKo‐1 cells for 12 h and 1 μM in Capan‐1/TP cells for 24 h).GThe effect of the thioparib and chloroquine combination on the viability of JeKo‐1 cells. Cells were pretreated with chloroquine for 4 h followed by thioparib treatment for 72 h and subjected to CCK‐8 assay. The combination index (CI) was calculated from three independent experiments using CompuSyn software and the average CI values are presented (CI <1, synergism; CI = 1, additive effect; CI >1 antagonism). Data are expressed as mean ± SEM. ThP—thioparib; OP—olaparib; TP—talazoparib; 391—Cpd‐391; CQ—chloroquine.HHR repair assay. U2OS‐DR‐GFP (WT) and PARP1 KO cells (KO1 and KO2) were treated with the indicated drugs for 48 h and collected after 72 h of I‐SceI transfection as in (A). VE‐821 served as a positive control. PARP1 knockout efficiency was evaluated by western blotting. Data from three biological replicates are presented as mean ± SD. Data information: Statistical analysis was performed by one‐way ANOVA, and each group was compared with the control. ***P < 0.0001, *P = 0.0143, 0.0483, ns: *P* = 0.3061, *P* = 0.8269, *P* = 0.9997, *P* = 0.3906 (from left to right). Source data are available online for this figure.

HR repair mechanisms play an important role in DSB repair that occurs during or after S‐phase DNA replication. A previous study demonstrated that PARP1 is required for HR repair by opening chromatin at DNA damage sites (Chen *et al*, 2019b). The fact that thioparib retains sensitivity to PARPi‐resistant cells carrying partial HR restoration raises the question whether thioparib has an effect on HR activity. Surprisingly, thioparib strongly, and in a concentration‐dependent manner, inhibited HR activity in U2OS‐DR‐GFP human osteosarcoma cell lines, as measured by the direct repeat (DR)‐GFP reporter assay, in which the ATR inhibitor VE‐821 was used as a positive control (Fig 5A). We found that thioparib inhibited up to 95% of HR activity at 1 μM for 48 h. By contrast, 1 μM talazoparib or Cpd‐391 displayed much weaker inhibitory effects than thioparib, while 1 μM olaparib had little effect on HR activity under the same conditions. Notably, a highly significant correlation was observed between the average cytotoxic IC_50_ values of the 4 PARPi in 12 hematological cell lines and their capacity of inhibiting HR activity (*r* = 0.9923, *P* = 0.0077, Fig 5B). These results suggest that thioparib treatment significantly suppresses HR repair activity, thus enhancing or restoring the sensitivity of thioparib in hyperactive HR and PARPi‐resistant cells.

**Figure EV3:**
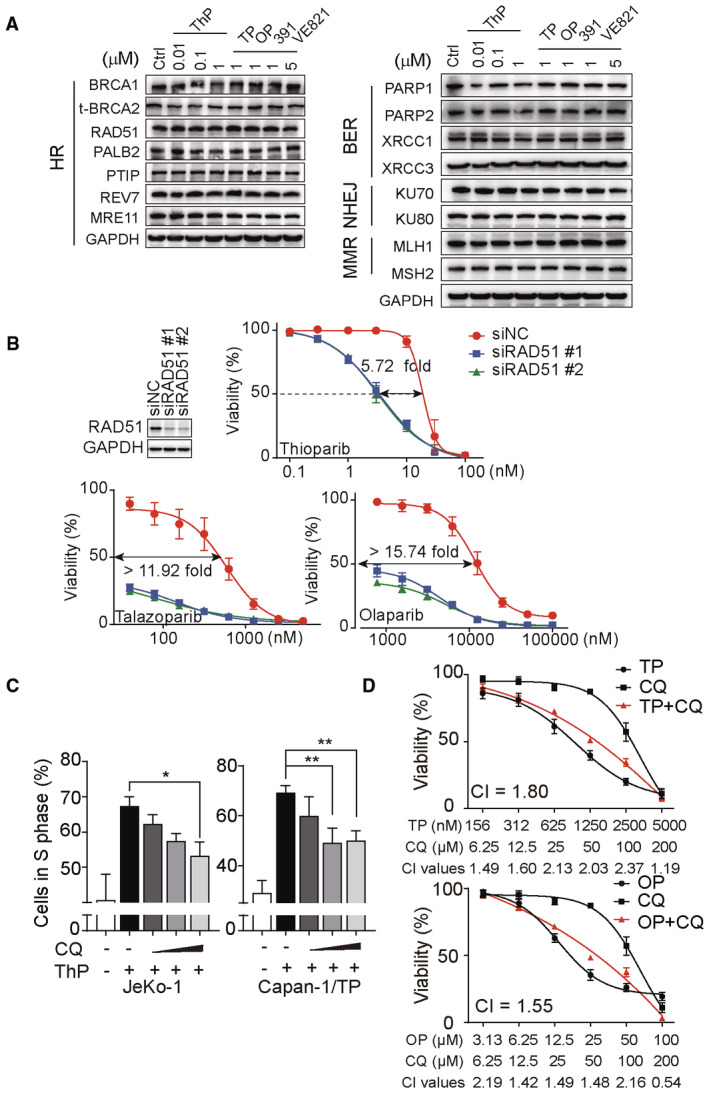
Effects of thioparib on DNA damage‐related proteins and the combination effects with chloroquine The changes in protein levels of DNA repair‐related proteins [including HR (left panel), BER, NHEJ, and MMR (right panel) pathways] following thioparib or the indicated drug treatment for 12 h.Depletion of RAD51 sensitized Capan‐1/TP cells to thioparib, olaparib, and talazoparib. Data from three biological replicates are shown as mean ± SEM.The percentage of cells in the S phase in JeKo‐1 and Capan‐1/TP cells treated with thioparib and chloroquine alone or in combination. The cell cycle was analyzed by PI staining‐based flow cytometry. Data are shown as the mean ± SD. Data from three independent experiments were analyzed by one‐way ANOVA. Left to right: *P = 0.0139, **P = 0.0072, *P* = 0.0093.The effect of chloroquine and the indicated PARP inhibitor combination on the viability of JeKo‐1 cells. Cells were pretreated with chloroquine for 4 h followed by talazoparib or olaparib treatment for 3 days and subjected to CCK‐8 assay. Average CI values from three independent experiments were presented. Data are expressed as the mean ± SEM. Data information: ThP, thioparib; OP, olaparib; TP, talazoparib; 391, Cpd‐391; CQ, chloroquine. Source data are available online for this figure.

As RAD51 activity is essential for HR repair, we examined the RAD51 foci formation in the presence or absence of thioparib after 6 Gy X‐ray in U2OS‐DR‐GFP cells. The resulting data showed a robust increase in RAD51 foci numbers when treated with thioparib alone or along with IR (Fig 5C). The cells also exhibited increasing numbers of RAD51 foci following talazoparib or Cpd‐391 treatment but not following treatment with 1 μM olaparib. Additionally, thioparib had no significant effects on the protein levels of HR‐, BER‐, NHEJ‐, and MMR‐related proteins (Fig EV3A). Previous works have shown that more RAD51 foci after PARPi treatment are induced by collapsed replication forks in HR‐competent cells (Bryant *et al*, 2005). In Fig 4, we showed that thioparib‐treated cells have a high level of γH2AX and p‐RPA32, likely representing an elevated level of spontaneously collapsed replication forks. Notably, a previous study showed that Polθ inhibitor induces excessive DSB end resection and nonfunctional RAD51 foci, leading to the cell death of PARPi‐resistant cancer. Because HR activity was greatly reduced upon thioparib treatment, elevated RAD51 levels appear nonfunctional for HR repair. To test this, we knocked down RAD51 using RNA interference in PARPi‐resistant Capan‐1/TP cells. To our surprise, we found that a lack of RAD51 causes increased sensitivity to thioparib, olaparib, and talazoparib (Fig EV3B). These data indicate that the inhibition of PARP by thioparib reduced HR reporter activity but induced excessive RAD51 foci formation.

As PARP1 has been shown to facilitate HR by promoting the relaxation of chromatin at DNA damage sites (Chen *et al*, 2019b), we proposed that thioparib treatment impairs cellular chromatin relaxation, leading to HR inhibition. However, by forcing the relaxation of chromatin using chloroquine (CQ), the HR activity showed no additional changes in thioparib‐treated cells (Fig 5D). Therefore, supplementation with CQ failed to rescue the HR efficiency of thioparib‐treated cells. Strikingly, CQ pretreatment prevented thioparib from inducing DNA damage and subsequent cell‐cycle arrest in both JeKo‐1 and Capan‐1/TP cells (Figs 5E and F, and EV3C). Importantly, the simultaneous thioparib/CQ combination and other PARPi/CQ combinations showed antagonism in JeKo‐1 cells (CI value > 1; Figs 5G and EV3D). These data suggest that CQ has different effects on thioparib‐induced DNA damage and HR suppression.

To further explore the role of PARP1 in mediating the HR suppression of thioparib, we generated PARP1#KO cells in the U2OS‐DR‐GFP background using CRISPR‐Cas9 technology. As expected, PARP1 depletion resulted in GFP‐positive frequencies greater than that of the wild‐type cells following thioparib or other PARPi treatment, suggesting that loss of PARP1 could potentially restore the HR repair efficiency in cells treated with PARPi. In the same condition, the HR defect induced by ATR inhibitor VE‐821 was not affected. However, we noted that the GFP‐positive frequencies of thioparib‐treated cells remained lower than those of other cells, suggesting an incomplete reversion of HR repair (Fig 5H). These results suggested that HR suppression induced by thioparib involves a combined effect of PARP1 inhibition and some other mechanisms.

### Thioparib induced a type I IFN response through PARP7‐STING/TBK1 and p38 MAPK pathways

Compared with the most potent PARPi talazoparib, thioparib is approximately 10‐fold more potent in inhibiting PARP1/2 activity but has a much greater potency advantage in inhibiting HR‐deficient and PARPi‐resistant cells. This suggests that the ability of thioparib to kill these cells may be partially due to PARP1/2‐independent activities. As shown in Fig 1E, in addition to PARP1‐3, thioparib also inhibited tankyrases TNKS1 and TNKS2 *in vitro* with the IC_50_ of 56.6 nM and 16.6 nM, respectively. However, we found that neither thioparib nor Cpd‐391 increased Axin2 protein levels, a marker of TNKS1/2 inhibition, in SW480 cells (Appendix Fig S2A).

**Figure 6:**
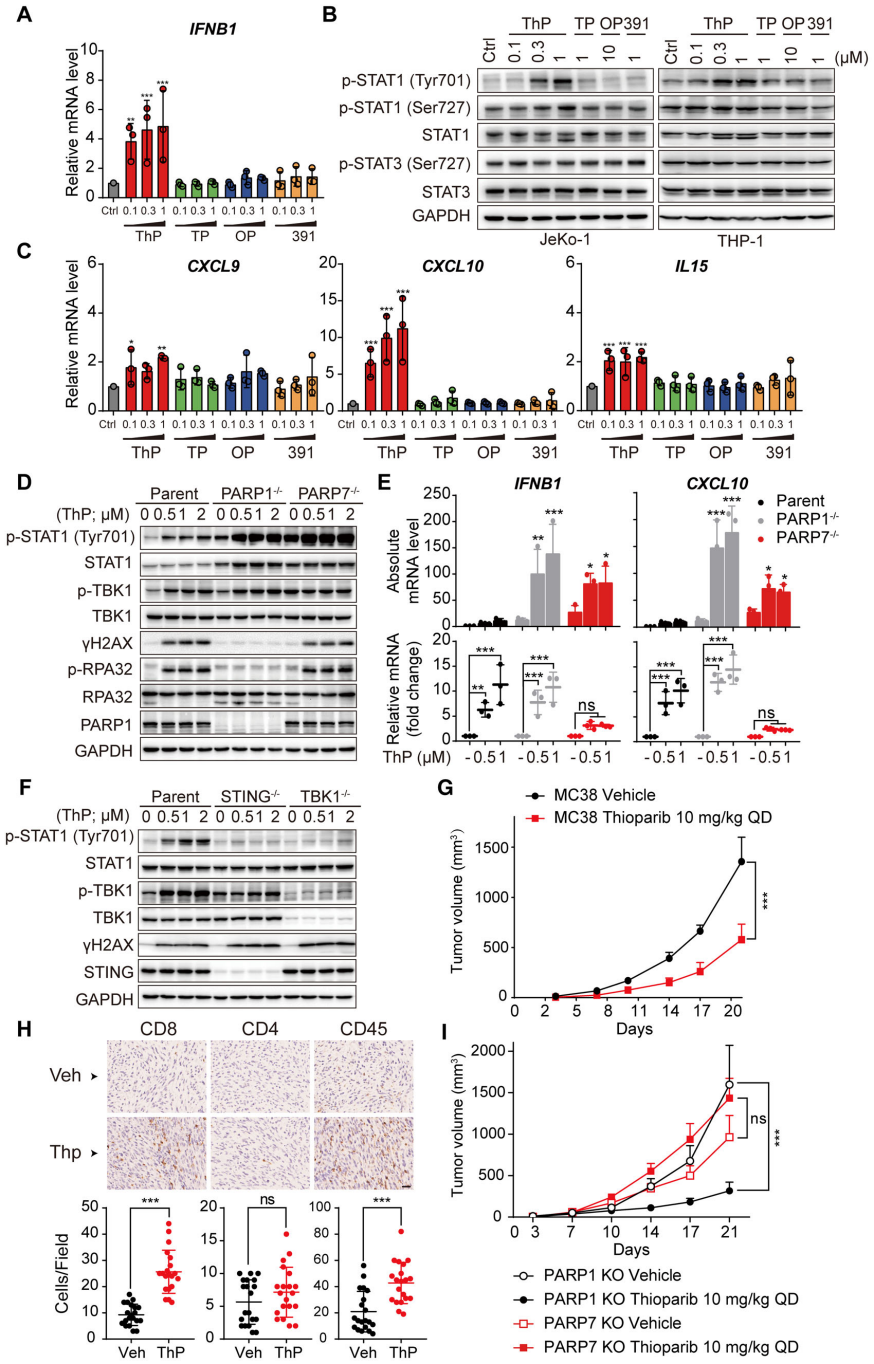
Thioparib induces a type I interferon (IFN) response in tumor cells through PARP7 inhibition *in vitro* and *in vivo* *IFN‐β* mRNA levels in JeKo‐1 cells treated with the indicated drugs for 12 h. Data from three biological replicates are depicted as mean ± SD. Statistical analysis was performed by two‐way ANOVA. ***P < 0.0001, **P = 0.0013.Concentration‐dependent increase in p‐STAT1 by thioparib in JeKo‐1 and THP‐1 cells.Effects of thioparib on *CXCL9*, *CXCL10*, and *IL15* mRNA in JeKo‐1 cells. Data from three independent experiments are shown as the mean ± SD. Statistical analysis was performed by two‐way ANOVA. Left panel: *P = 0.0367, **P = 0.0011; median panel: ***P = 0.0002, *P* < 0.0001, *P* < 0.0001; right panel: ***P = 0.0005, *P* = 0.0009, *P* = 0.0001 (from left to right).The protein levels of p‐STAT1, p‐TBK1, γH2AX, and phospho‐RPA32 in HT‐29 parent, PARP1^−/−^ (#KO1), and PARP7^−/−^ cell lines after 24 or 48 h thioparib treatment.The changes in *IFNB1* and *CXCL10* mRNA levels in HT‐29 parent, PARP1, and PARP7 KO cells exposed to thioparib. Data from three biological replicates are shown as the mean ± SD. Statistical analysis was performed by two‐way ANOVA. Upper panel: the absolute mRNA levels by normalizing against the parent untreated group, and *P*‐value was calculated compared with the parent untreated group, *P = 0.0125, *P* = 0.0105, *P* = 0.0187, *P* = 0.0349 (from left to right), **P = 0.0021, ***P < 0.0001. Lower panel: the relative mRNA fold change normalized against the untreated group in each cell line, and *P*‐value was calculated compared with the untreated group in each cell line, ns, *P* = 0.3194, *P* = 0.3373, *P* = 0.4158, *P* = 0.4907, **P = 0.0070, ***P < 0.0001, *P* = 0.0009, *P* < 0.0001, *P* < 0.0001, *P* < 0.0001, *P* < 0.0001, *P* < 0.0001 (from left to right).Western blot analysis of p‐STAT1, p‐TBK1, and γH2AX in HT‐29 parent, STING^−/−^, and TBK1^−/−^cells after 24 or 48 h thioparib treatment.Antitumor activity of 10 mg/kg thioparib in MC38 syngeneic tumor model. Tumor‐bearing C57BL/6J mice were dosed orally with vehicle or thioparib for 21 days (*n* = 6). *P*‐value was determined by two‐way ANOVA, ***P < 0.0001. QD, once daily. Data are presented as the mean ± SEM.Immunohistochemistry staining of CD4, CD8, and CD45 expression in MC38 tumor tissue sections. Quantification of cells positive for CD4, CD8, and CD45 was shown. Scale bar: 25 μm. Data from 10 technical replicates from each of the two mice are presented as mean ± SD. Statistical analysis was performed by unpaired *t*‐test. ***P < 0.0001, ns: *P* = 0.1958.Antitumor activity of 10 mg/kg thioparib in C57BL/6J subcutaneous tumor model using MC38 PARP1 KO or PARP7 KO cells (*n* = 6). *P*‐value was determined by two‐way ANOVA. ***P < 0.0001, ns, *P* = 0.0597. Data are presented as the mean ± SEM. Source data are available online for this figure.

PARPi (olaparib and talazoparib) have been reported to activate IFN signaling genes through the stimulator of interferon genes (STING) pathway (Pantelidou *et al*, 2019). Moreover, the PARP7 inhibitor RBN‐2397 activates the type I IFN response by inducing STAT1 phosphorylation through STING/TBK1 signaling (Gozgit *et al*, 2021). Considering that thioparib inhibited PARP7 *in vitro* (Fig 1E), we next investigated whether it was also the case in thioparib‐treated cells. As shown in Fig 6A, *IFNβ1*, a key upstream gene in the type I IFN pathway, was concentration‐dependently increased in JeKo‐1 cells following thioparib treatment. Importantly, thioparib also led to a concentration‐dependent increase in the phosphorylation of the transcriptional activator STAT1 (p‐STAT1‐Y701), whereas it exhibited little effect on the phosphorylation of STAT3 (p‐STAT3‐S727) (Fig 6B). Accordingly, the transcription of IFN‐related genes, including *CXCL9*, *CXCL10*, and *IL15*, was significantly enhanced by thioparib in JeKo‐1 cells (Fig 6C). These effects were not observed in cells exposed to talazoparib, olaparib, or Cpd‐391. Consistent with these results, thioparib‐induced activation of STAT1 and IFN‐related STAT1 target genes was also enhanced in THP‐1 and HT‐29 cell lines (Fig 6B; Appendix Fig S2B and C).

**Figure EV4:**
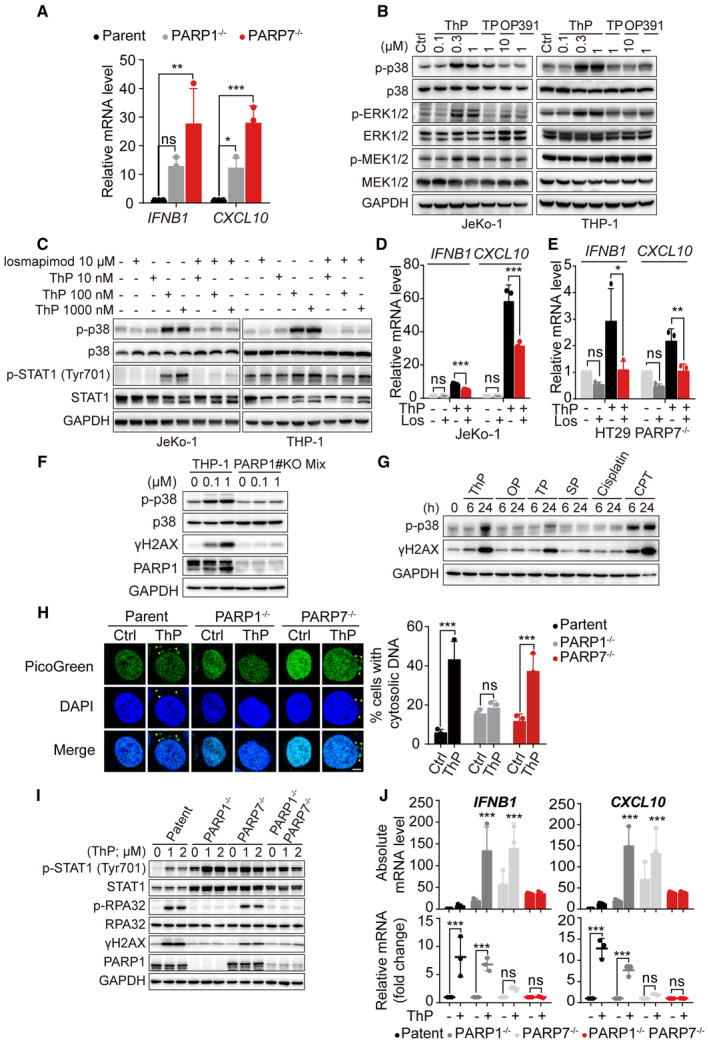
Effects of thioparib on p38 MAPK signaling A
*IFN‐β* and *CXCL10* mRNA levels in HT29 PARP1^−/−^ or PARP7^−/−^ cells. Statistical analysis was performed by one‐way ANOVA. ns: *P* = 0.1558, **P = 0.0075, *P = 0.0225, ***P = 0.0003. Mean ± SD of three independent experiments is shown. Data were from Fig 6E.BEffects of thioparib on MAPK signaling determined by western blotting in JeKo‐1 and THP‐1 cells, followed by PARPi treatment for 12 h.CInhibition of p38 activity with losmapimod prevents thioparib‐induced p‐STAT1 in JeKo‐1 and THP‐1 cells.D, EInhibition of p38 activity with losmapimod reverses the increase in *IFNB1* and *CXCL10* mRNA levels induced by thioparib in JeKo‐1 (D) and HT‐29 PARP7^−/−^ (E) cells. Cells were pretreated with 10 μM losmapimod for 4 h, followed by thioparib treatment. Data are shown as the mean ± SD from three independent experiments. Statistical analysis was performed by one‐way ANOVA. Left panel: ns: *P* = 0.7380, *P* > 0.9999, ***P < 0.0001, *P* = 0.0009; right panel: ns: *P* = 0.8173, *P* = 0.1596, *P = 0.0301, **P = 0.0036 (from left to right).FThe activation of p38 by thioparib is abolished by *PARP1* depletion. The wild‐type and *PARP1* knockout THP‐1 cells were treated with the indicated concentrations of thioparib and subjected to western blotting with the indicated antibodies.GPhosphorylation of p38 MAPK is associated with increased DNA damage. JeKo‐1 cells were treated with the indicated agents, and p‐p38 and γH2AX were detected using western blotting.HImmunofluorescence microscopic analysis of cytosolic dsDNA in wild type, PARP1^−/−^ and PARP7^−/−^ HT‐29 cells treated with or without thioparib (1 μM for 36 h). Left, representative images of PicoGreen (green; dsDNA) and DAPI (blue; nucleus) staining. The yellow arrowheads indicate the occurrence of cytosolic dsDNA. Scale bar: 5 μm. Right, the graph shows the percentage of cells with cytosolic dsDNA (*n* > 100 cells each) from three biological replicates. Data are shown as the mean ± SD. Statistical analysis was performed by two‐way ANOVA. ***P < 0.0001, *P* = 0.0005 (from left to right), ns*P* = 0.9065.IThe protein levels of p‐STAT1, p‐RPA32, and γH2AX in HT‐29 parent, PARP1, 7 single or double knockout cells after thioparib treatment.JThe changes in *IFNB1* and *CXCL10* mRNA levels in HT‐29 parent, PARP1, 7 single or double KO cells exposed to thioparib. Data from three independent experiments are shown as the mean ± SD. Statistical analysis was performed by two‐way ANOVA. Upper panel: the absolute mRNA levels by normalizing against the parent untreated group, and *P*‐value was calculated compared with the parent untreated group, ***P = 0.0004, *P* = 0.0003, *P* = 0.0001, *P* = 0.0004 (from left to right). Lower panel: the relative mRNA fold change normalized against the untreated group in each cell line, and *P*‐value was calculated compared with the untreated group in each cell line, ns: *P* = 0.5233, *P* > 0.9999, *P* = 0.6815, *P* > 0.9999, ***P < 0.0001, *P* = 0.0002, *P* < 0.0001, *P* < 0.0001 (from left to right). Data information: ThP, thioparib; OP, olaparib; TP, talazoparib; 391, Cpd‐391; Los, losmapimod; SP, simmiparib; CPT, irinotecan. Source data are available online for this figure.

To further clarify the on‐target activity of thioparib on type I IFN signaling, we used CRISPR/Cas9 to knockout PARP1 and PARP7. Sequencing of the *PARP7* gene identified insertion/deletion mutations resulting in frameshift mutations in exon 2, and KO of PARP7 was further confirmed by RT–qPCR (Appendix Fig S2D). Under untreated conditions, we found that PARP7 KO cells presented high levels of phosphorylated STAT1 when compared to parental cells (Fig 6D). In agreement, PARP7 KO strongly upregulated *IFNB1*, and *CXCL10* gene expression, while PARP1 KO had a smaller effect on type I IFN signaling (Fig EV4A). The additional increase in STAT1, TBK1 phosphorylation, and *IFNB1* or *CXCL10* mRNA by thioparib was almost attenuated in HT‐29 cells that stable knockout (KO) of PARP7 (Fig 6D and E). However, the depletion of PARP1 had a slight effect on the induction of type I IFN signaling by thioparib. Notably, p‐STAT1 was greatly increased under basal conditions in PARP1 KO cell lines, which might be due to DNA damage‐induced accumulation of cytosolic dsDNA upon PARP1 depletion. As a result, thioparib had a stronger effect on p‐STAT1 and type I IFN signaling in PARP1 KO cells (Fig 6D and E). It was also noted that basal levels of STAT1 are increased in the PARP1 KO1 clone (Fig 6D) but not in the KO2 clone (Appendix Fig S2E). However, regardless of the levels of STAT1, treatment of PARP1 KO variants with thioparib led to a significant increase in p‐STAT1. We also assessed the effects of the specific PARP7 inhibitor RBN‐2397 in these cell lines and showed that PARP7 knockout is sufficient to prevent the additional increase in STAT1 phosphorylation and type I IFN signaling (Appendix Fig S2F and G). These results demonstrate that thioparib induces a type I IFN response by inhibiting PARP7 activity. Interestingly, the increase in the levels of γH2AX and p‐RPA32 by thioparib was significantly inhibited after PARP1 knockout, while PARP7 depletion had no significant effect (Fig 6D). Consistently, the PARP7 inhibitor RBN‐2397 did not increase the protein levels of γH2AX and p‐RPA32 (Appendix Fig S2F).

Given that STING‐TBK1‐IRF3 and MAPK cascades (including MEK, ERKs, p38, and JNKs) play essential roles in the regulation of STAT1 (Fang *et al*, 2013; Meissl *et al*, 2017), we first evaluated the effects of thioparib on these two main pathways. As shown in Appendix Fig S2H, thioparib treatment did not induce significant changes in the phosphorylated and basal protein levels of STING, TBK1, or IRF3 in JeKo‐1 cells. Increases in p‐STING and p‐TBK1 were detectable after thioparib treatment in THP‐1 cells; however, the protein level of p‐IRF3 remained unchanged (Appendix Fig S2H). Surprisingly, KO of STING or TBK1 is sufficient to prevent thioparib‐induced STAT1 phosphorylation in HT29 cells (Fig 6F). Likewise, type I IFN response induced by RBN‐2397 was diminished in STING or TBK1 KO cells, demonstrated by abrogation of p‐STAT1; while a highly selective PARP1 inhibitor AZD5305 had no effect on p‐STAT1 (Appendix Fig S2I). Together, these results indicate that PARP7‐STING/TBK1 signaling is likely a key player in thioparib‐induced type I IFN response.

Given that MAPK pathways appear to play essential roles in the induction of IFN responses (Fang *et al*, 2013), we next determined the effect of thioparib on the phosphorylation of MAPK family members, p38, ERK1/2, and MEK1/2. As shown in Fig EV4B, phosphorylation of p38 and ERK1/2 was significantly increased after 12 h of thioparib treatment in both JeKo‐1 and THP‐1 cells, while only a slight effect was observed on the phosphorylation of MEK1/2. Consistently, the enhanced phosphorylation of p38 was detectable as early as 3 h and then augmented with time (Appendix Fig S2J). Notably, the time course of p38 activation was faster than that of ERK or STAT1 activation. Furthermore, the thioparib‐mediated increase in p‐STAT1‐Y701 was blocked by the p38 inhibitor losmapimod (Fig EV4C) but not by the ERK inhibitor LY3214996 (Appendix Fig S2K). Moreover, the levels of *IFNB1* and *CXCL10* mRNA were significantly lower following losmapimod treatment (Fig EV4D). As shown in Fig 6D and E, we noticed that PARP7^−/−^ cells retained a slight increase in the phosphorylation of STAT1 and IFNB1/CXCL10 mRNA levels upon thioparib treatment. However, supplementation with losmapimod reversed these effects in HT29 PARP7^−/−^ cells (Fig EV4E). These data imply that p38 MAPK activation was an early event after thioparib treatment, while type I IFN signaling may serve as a downstream mediator. These data indicate that both PARP7‐STING/TBK1 and p38 MAPK signaling are centrally involved in the thioparib‐induced type I IFN response. Thioparib did not have significant interaction with the p38 family, ERK1/2, and MEK1/2 (Appendix Fig S2L). Interestingly, we found that p38 activation after thioparib treatment was abolished by PARP1 KO (Fig EV4F). It has been suggested that the p38 pathway was mainly activated by stress signals, including DNA damage (Wood *et al*, 2009). Indeed, p38 MAPK activation is associated with high γH2AX expression (Fig EV4G). These data suggest that thioparib activates p38 MAPK signaling most likely through the induction of DNA damage. It has been reported that PARPi treatment generates cytosolic dsDNA in a PARP1‐dependent manner (Kim *et al*, 2020). Consistent with previous studies, our data showed that thioparib treatment results in the robust production of cytosolic dsDNA (Fig EV4H). Notably, cytosolic dsDNA accumulation can be prevented by the depletion of PARP1 but not PARP7. Moreover, compared with PARP7 single‐KO, the double KO of PARP1 and PARP7 completely diminished thioparib‐induced type I IFN response (Fig EV4I and J). These *in vitro* observations suggested that the inhibition of PARP1 might also contribute to the IFN response activation.

To investigate the induction of antitumor immune response and therapeutic effects of thioparib *in vivo*, we orally dosed MC38 tumor‐bearing, immune‐competent female C57BL/6J mice with vehicle or thioparib (10 mg/kg) once daily. As shown in Fig 6G, thioparib effectively inhibited the growth of MC38 tumors in mice. Consistently, immunohistochemical staining demonstrated that the proportion of CD8^+^ T cells increased significantly in thioparib‐treated tumors, whereas the proportion of CD4^+^ T cells was not changed (Fig 6H). Using MC38 KO cell lines, we found that PARP7 is indispensable for the antitumor effect of thioparib in immune‐competent mice compared with PARP1. As shown in Fig 6I, administration of thioparib (10 mg/kg, qd) for 3 weeks significantly suppressed tumor growth compared with vehicle (*P* < 0.001) in the PARP1 KO MC38 model; however, no antitumor activity was observed in PARP7 KO tumor‐bearing mice. Immunohistochemical staining results of CD4, CD8, and CD45 from PARP1/7 KO tumors obtained from the same experiment were also consistent with the observed *in vivo* antitumor activity (Appendix Fig S3A and B). Collectively, these data show direct evidence that thioparib induces immune responses and antitumor effects *in vivo* and strongly suggest that PARP7 is indispensable in this respect.

### CRISPR screening identifies determinants of thioparib sensitivity

**Figure 7:**
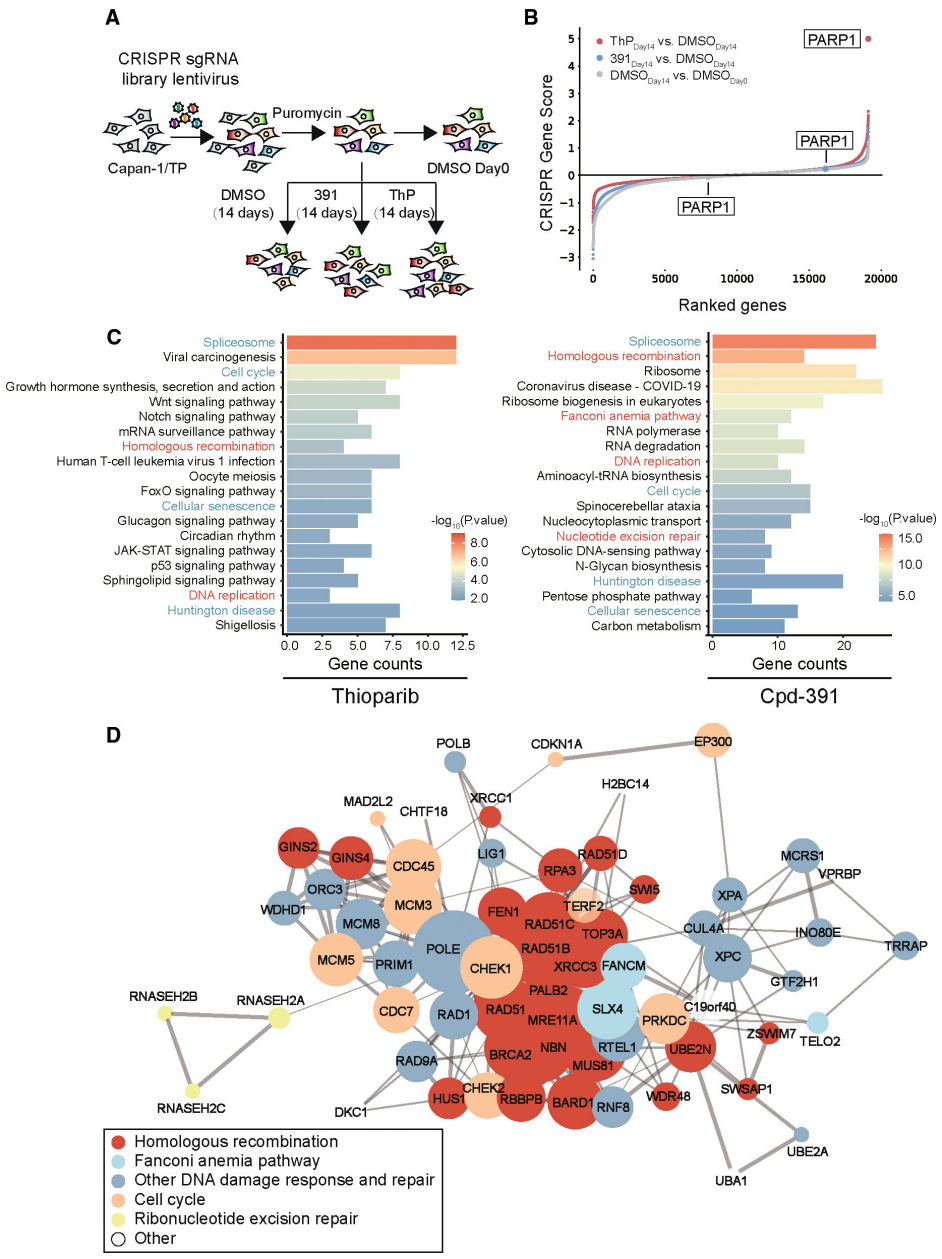
Genome‐wide CRISPR‐Cas9 screening identifies determinants of the thioparib response Experimental scheme of CRISPR‐Cas9 knockout (GeCKO) screening.CRISPR gene score analysis revealed that *PARP1* knockout led to resistance of thioparib and Cpd‐391 in PARPi‐resistant Capan‐1/TP cells but did not affect the viability of cells treated with DMSO. Red dots: thioparib group; blue dots: Cpd‐391 group; gray dots: DMSO group.Top 20 enriched KEGG pathway analysis of the genes in the top candidates (with ≥3 sgRNA hits, *P*‐value ≤ 0.05, and log fold change ≤ −0.5) whose disruptions increase sensitivity to thioparib (left panel) or its enantiomer Cpd‐391 (right panel).Network analysis of potential interactions between hits among thioparib and Cpd‐391 using STRING protein–protein interaction networks. Sixty‐six genes enriched in the DNA damage response (DDR) pathway and cell‐cycle pathway by GO analysis were mapped on the network. Data information: ThP, thioparib; 391, Cpd‐391.

Previous studies have demonstrated that most PARPi selectively inhibit tumor cells with genetic defects in HR and DNA damage response (DDR) (Curtin & Szabo, 2020; Dias *et al*, 2021). To identify new determinants of thioparib sensitivity or resistance, we performed genome‐wide screening using a CRISPR‐Cas9 knockout library (with 76,441 sgRNAs targeting 19,114 human genes) and compared the genetic sensitization/resistance profile of thioparib with its isomer Cpd‐391. As shown in Appendix Table S4, PARPi‐resistant Canpan‐1/TP cells remained sensitive to thioparib but were highly resistant to Cpd‐391 and olaparib. We then selected Canpan‐1/TP cells to perform this screening. The experimental procedure of CRISPR screening is shown in Fig 7A and in the Materials and Methods.

Using CRISPR Gene Score analysis, we observed that *PARP1* was the top candidate selected in the thioparib_Day 14_ group, suggesting that PARP1 was required for the survival of PARPi‐resistant Capan‐1/TP cells upon thioparib treatment (Fig 7B). *PARP1* showed no significant change in the DMSO_Day14_ group, indicating that knockout of *PARP1* did not affect the proliferation of Capan‐1/TP cells. The MAGeCK algorithm was implemented to further analyze the data (Li *et al*, 2014, 2015). The sgRNAs targeting *PARP1* were again selected out in the insensitive parts of the MAGeCK analysis (detailed data in Dataset EV1), suggesting that cells with *PARP1* KO were significantly resistant to thioparib. Indeed, PARP1 depletion using the same sgRNAs resulted in thioparib resistance (Fig 4H; Appendix Table S6). Thus, we conclude that *PARP1* is a key gene in determining the sensitivity of Canpan‐1/TP cells to thioparib. By contrast, *PARP2*, *PARP3*, *TNKS1*, *TNKS2*, and *PARP7* showed no significant changes during the screening.

In PARPi‐resistant Capan‐1/TP cells, thioparib was 35‐fold more potent than its isomer Cpd‐391 in inhibiting cell proliferation (IC_50_ 1.49 nM versus 53.36 nM), contrasting the 2‐fold potency difference in PARP1 activity inhibition. Next, we compared the major determinants of sensitivity/resistance to both compounds. The Kyoto Encyclopedia of Genes and Genomes (KEGG) pathway enrichment analysis of 256 and 706 genes (with ≥3 sgRNA hits, *P*‐value ≤0.05, and log fold change ≤ −0.5), the inactivation of which caused sensitization to thioparib or Cpd‐391, respectively, showed strong enrichment for biological processes related to homologous recombination, DNA replication, and the Fanconi anemia pathway (Fig 7C; Appendix Fig S4A). These data confirmed that the selected genes are the bona fide regulators of PARPi. We also identified four pathway‐based gene signatures, including spliceosome, cell cycle, cellular senescence, and Huntington disease, which were common to both compounds. Mapping the 66‐gene set using the STRING protein–protein interaction networks (Fig 7D) generated a highly connected network consisting of DNA damage response genes that include many regulators of homologous recombination (such as BRCA2, RAD51B, RAD51C, and PALB2), components of the Fanconi anemia pathway (such as FANCM, TELO2, and SLX4), as well as the CHEK1 and XPC. Outside or at the edge of the network, we noted the presence of genes encoding the ribonucleases RNASEH2A, RNASEH2B, and RNASEH2C, histone acetyltransferase EP300, and genes coding the nucleolar protein MCRS1.

Similarly, KEGG pathway enrichment analysis of 256 genes (with ≥ 3 sgRNA hits; *P*‐value ≤ 0.01, and log fold change ≥ 1) whose knockout led to thioparib resistance showed significant enrichment in thermogenesis, oxidative phosphorylation, and aminoacyl‐tRNA biosynthesis pathways (Appendix Fig S4B) but with no significant enrichment results observed in the Cpd‐391 screening group. These results were common to the gene profile of olaparib, suggesting similar functions between thioparib and olaparib in targeting PARP1 and subsequent cellular response (Clements *et al*, 2020).

**Figure EV5:**
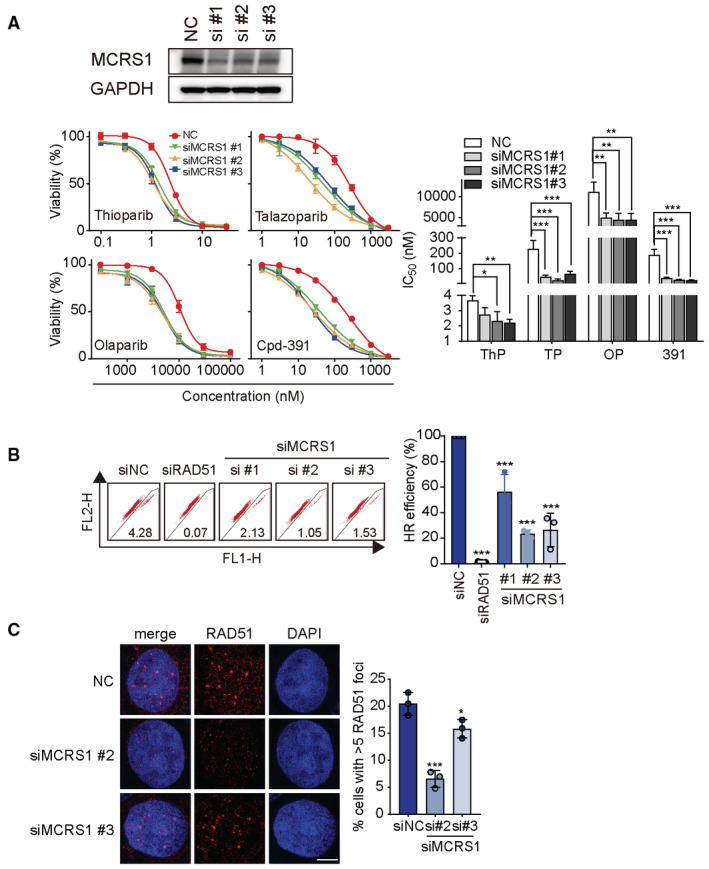
MCRS1 depletion enhances PARPi sensitivity due to defective HR MCRS1 knockdown increased the sensitivity of Capan‐1/TP cells to the indicated PARPi. Statistical analysis was performed by one‐way ANOVA. Left to right: *P* = 0.0146, *P* = 0.0096, *P* = 0.0002, *P* = 0.0001, *P* = 0.0005, *P* = 0.0050, *P* = 0.0029, *P* = 0.0030, *P* < 0.0001, *P* < 0.0001, *P* < 0.0001. In the left panel, data from three independent experiments are presented as mean ± SEM. In the right panel, IC_50_ values from the same experiments are shown as mean ± SD.HR‐mediated DNA repair is impaired in MCRS1‐depleted U2OS‐DR‐GFP cells. U2OS‐DR‐GFP reporter cells were transfected with siRNAs targeting MCRS1, RAD51 (positives control), or negative control for 24 h, followed by I‐SceI transfection. Data from three biological replicates are shown as the mean ± SD. Statistical analysis was performed by one‐way ANOVA. Left to right: ***P < 0.0001, *P* = 0.0003, *P* < 0.0001, *P* < 0.0001.Reduced RAD51 foci formation in MCRS1‐depleted Capan‐1/TP cells after 6 Gy of ionizing radiation (IR) exposure. Graphs (right panel) show the quantification of cells with ≥ 5 RAD51 foci. Scale bar: 5 μm. Data represent the mean ± SD. Data from three independent experiments were analyzed by one‐way ANOVA, ***P = 0.0002, *P = 0.0353. ThP, thioparib; OP, olaparib; TP, talazoparib; 391, Cpd‐391. Source data are available online for this figure.

In the list of the 47 common genes (Appendix Fig S4A) that determine sensitivity to both thioparib and Cpd‐391, we noticed MCRS1, which has not previously been linked to the response to PARP inhibition. We next examined how this gene affects the sensitivity to thioparib and other PARPi. Knockdown of MCRS1 increased the sensitivity of PARPi‐resistant Capan‐1/TP cells to thioparib by 2‐fold (Fig EV5A). Indeed, siMCRS1 also increased the cellular sensitivity of talazoparib, olaparib, and Cpd‐391 by up to 9‐, 3‐, and 7‐fold, respectively. As deficiency in HR repair is frequently associated with PARPi sensitivity, we next detected whether MCRS1 is involved in this pathway. Indeed, MCRS1 knockdown using three independent siRNAs remarkably decreased the HR efficiency, as assessed by the direct repeat‐green fluorescent protein (DR‐GFP) assay (Fig EV5B). Additionally, we observed impaired RAD51 foci formation in MCRS1‐depleted cells, which further strengthened the deficiency in HR‐meditated DNA repair (Fig EV5C). These results indicate that the depletion of MCRS1 sensitizes PARPi‐resistant Capan‐1/TP cells to thioparib and other PARPi by inhibiting HR activity.

## Discussion

In this study, we performed a hit finding using a combination of ELISA‐based screening and DSB FA assay detection. These efforts led to the identification of thioparib, which displayed impressive results in both PARP1 enzyme inhibition, with an IC_50_ value of 0.13 nM, and PARP1‐DNA binding enhancement, with an EC_50_ value of 25.05 nM. Compared with other PARPi, thioparib as a single agent has a much greater potency advantage in inhibiting BRCA‐deficient breast, pancreatic cancer, and HR‐deficient hematological cancer cells (Figs 1 and 3). Importantly, tumors that acquire olaparib resistance by partially restoring HR repair (e.g., *53BP1* loss or *BRCA1*‐mutant restoration) are likely to remain sensitive to thioparib (Fig 2). Thioparib was also effective in multiple *in vivo* xenograft models. Importantly, mechanistic studies revealed that thioparib displayed both high HR repression activity (Fig 5) and immunologic properties (Fig 6).

Several mechanisms of PARPi resistance have been described, including restoration of HR capacity, stabilization of replication forks, diminished trapping of PARP1, and P‐gp‐mediated drug efflux (Li *et al*, 2020; Dias *et al*, 2021). Previous studies have revealed that Polθ inhibitors overcome some but not all mechanisms of PARPi resistance. For example, PARPi‐resistant cells with 53BP1 defects are sensitive to the Polθ inhibitor NVB *in vitro* and *in vivo*; however, other PARPi‐resistant cells with the stabilized mutant BRCA1 protein or somatic reversion of BRCA2 were relatively resistant to its treatment (Zhou *et al*, 2021). In this study, we demonstrated that thioparib exhibited strong anti‐proliferative activity (average IC_50_ = 3.39 nM) in several PARPi‐resistant models, representing different mechanisms of acquired PARPi resistance. While highly resistant to olaparib, MDA‐MB‐436#53BP1, MDA‐MB‐436#AZD2281‐R1, and BR‐05‐0028 xenograft tumors were highly sensitive to thioparib (Fig 2). Hematologic cancer cell lines carrying at least one mutation in the HR gene, except for BCR‐ABL1‐positive lines, were also relatively sensitive to thioparib. Additionally, HR‐proficient MM1S myeloma cell lines were highly sensitive to thioparib by an unknown mechanism (Fig 3). Therefore, our data suggest that thioparib may overcome multiple mechanisms of acquired resistance to PARPi *in vitro* and *in vivo*. Moreover, other tumors defective in HR, including some hematological malignancies, are also good candidates for thioparib monotherapy.

Previous studies showed that PARP1 depletion or inhibition significantly decreased HR activity in U2OS‐DR‐GFP or HCA2‐hTERT cell lines (Jelinic & Levine, 2014; Chen *et al*, 2019b). Accordingly, our data revealed that thioparib activates S‐phase arrest and strongly suppresses HR repair. Even at a low concentration (10 nM thioparib), which did not cause any obvious cell‐cycle alteration, HR repair was significantly inhibited following treatment. Notably, the ability of PARPi to inhibit HR repair seems to directly correlate with their cytotoxic potential. We also found that thioparib triggered RPA32 and Chk1 phosphorylation, indicative of strong replication stress. Additionally, thioparib triggered γH2AX accumulation and RAD51 foci formation but did not alter expression levels of RAD51 or other HR‐related proteins. Therefore, we propose that thioparib might function to treat cancer by blocking HR activity, leading to the accumulation of DNA damage. Furthermore, thioparib treatment resulted in a dramatic decrease in HR activity, suggesting that thioparib can be used alone for treating hyperactive HR tumors, even after the acquisition of olaparib resistance.

In this work, the mechanistic insights into how thioparib impairs HR repair remain to be investigated. A previous study indicated that cell‐cycle status influences HR outcomes, while olaparib decreases HR activity by reducing replicative S‐phase cells (Jelinic & Levine, 2014). However, a 20% increase in the percentage of cells in the S phase was observed upon thioparib treatment. Moreover, supplementation with CQ could prevent DNA damage accumulation and reduce S‐phase arrest in thioparib‐treated cells but failed to rescue HR efficiency, suggesting that cell‐cycle status did not associate with HR repression by thioparib. Interestingly, we find that loss of PARP1 is able to partially rescue the suppression of HR by thioparib. In addition to inhibiting PARP catalytic activity, PARPi trap PARP1/2 on DNA. Another study has revealed that PARP1 regulates HR by reducing nucleosome density at DNA damage sites, while PARPi suppress PAR formation to reduce chromatin relaxation and impair HR (Chen *et al*, 2019b). In this model, PAR plays a vital role in clearing nucleosomes through the recruitment of BRG1 and SIRT1 to damage sites, thereby promoting HR repair. Using DSB FA models, we observed that thioparib displayed ~ 2‐ and 24‐fold more potent at inhibiting PARylation on DNA than talazoparib and olaparib, respectively (Appendix Table S1). Furthermore, thioparib had the strongest effect on HR function among the four PARPi we tested (Fig 5). These results led us to conclude that the effect of PARPi on HR repair in U2OS‐DR‐GFP cells is dependent on its potency in inhibiting PAR formation on DNA sites. Nevertheless, whether thioparib inhibits HR through modulating nucleosome density remains to be further determined. Additionally, our data suggest that thioparib also impairs HR repair in a way that differs from that of other PARPi, although the precise mechanisms remain elusive.

Type I IFN cytokine IFN‐β can activate STAT1, and subsequently induces type I IFN gene expression. Indeed, our results showed that thioparib dramatically increased the transcription levels of *IFNB1* and a range of IFNγ‐induced genes (*CXCL9*, *CXCL10*, and *IL15*), suggesting that thioparib stimulates the production of type I IFNs. PARPi have been reported to promote the accumulation of cytosolic DNA fragments, which in turn activate the innate immune cGAS‐STING signaling (Ding *et al*, 2018; Pantelidou *et al*, 2019; Shen *et al*, 2019). In this study, we were unable to detect similar STING activation (upregulation of p‐TBK1 and p‐IRF3) following PARPi treatment in JeKo‐1 cells. However, the knockout of STING or TBK1 could prevent thioparib‐induced STAT1 phosphorylation. Additionally, thioparib showed relatively low potency against PARP7, whereas KO of PARP7 attenuated an additional increase in STAT1 phosphorylation and type I IFN response. We also observed enhanced type I IFN signaling by RBN‐2397 after PARP1 depletion, suggesting that PARP1 inhibition may enhance the antitumor immunity of the PARP7 inhibitor. Consistently, PARP1 was selected in the CRISPR screening of RBN‐2397 as previously reported (Gozgit *et al*, 2021). Therefore, the dual inhibition of both PARP1 and PARP7 may be a feasible strategy for antitumor immunity. Interestingly, our data also indicate that the activation of p38 MAPK is associated with PARP1‐dependent DNA damage, which may partially contribute to thioparib‐induced STAT1 activation. Therefore, we conclude that thioparib treatment triggers the activation of STING/TBK1 and p38, which collaborate to induce the expression of type I IFNs and elicit innate antitumor immunity. Moreover, thioparib treatment resulted in robust production of cytosolic dsDNA only in the presence of PARP1 protein. Given that the type I IFNs signaling was also slightly induced by thioparib in PARP7 KO cells, we thus proposed that PARP1 inhibition with thioparib treatment leads to replication‐associated DNA damage, triggers the release of cytosolic dsDNA, and subsequently activates the cGAS/STING signaling. Our *in vivo* data suggest that the antitumor activity of thioparib can be achieved in the immunocompetent MC38 mouse model and support that PARP7, but not PARP1, makes a major contribution to the immunomodulatory activity of thioparib. Together, these data suggest that thioparib treatment elicits strong immune responses and antitumor effects *in vitro* and *in vivo*. Consistent with the *in vitro* data (Fig 6D and E), immunocompetent PARP1 KO MC38 mice respond better to thioparib treatment, suggesting that a specific PARP7 inhibitor, rather than a pan‐inhibitor such as thioparib, would be more relevant for clinical applications. However, based on our data (Fig 2), thioparib is potent against PARP1i‐resistant cancer cell lines, which remains to be tested in more mouse models.

Although several reports have performed CRISPR/Cas9 screening to identify modifiers of the PARPi response (Zimmermann *et al*, 2018; Wei *et al*, 2019; Clements *et al*, 2020), our study is the first to report MCRS1 as a candidate modifier of response to PARPi. MCRS1 was originally described as a RanGTP‐regulated factor essential for noncentrosomal microtubule assembly. A subsequent study described its additional role in the DNA damage and the p53/p21 senescence pathway (Hsu *et al*, 2012). Here, increased sensitivity to thioparib and other PARPi was observed upon depletion of MCRS1 in PARPi‐resistant Capan‐1/TP cells (Fig EV5). These results suggest that the depletion of MCRS1 results in re‐sensitization of PARPi‐resistant cells to PARPi, which is consistent with our observation that MCRS1 knockdown impaired homologous recombination repair and reduced the formation of IR‐induced Rad51 foci (Fig EV5). Our results advance the current understanding by demonstrating that MCRS1 is essential for HR repair.

Profiled head‐to‐head, thioparib was more potent than olaparib *in vitro* and *in vivo*. We also observed a large (> 50‐fold) difference in cellular potency between thioparib and its enantiomer Cpd‐391, although they were comparable at inhibiting PARP catalytic activity. Except for PARP1, we also assessed the effects of thioparib and Cpd‐391 on TNKS1/2 and PARP7 inhibition in cell‐based assays. We did not observe significant stabilization of Axin2 in SW480 cells following treatment with thioparib or Cpd‐391, although both compounds displayed high TNKS1/2 inhibition (IC_50_: 16.6–56.6 nM). However, thioparib significantly increased *IFNB1* and *CXCL10* mRNA levels in a PARP7‐dependent manner; by contrast, Cpd‐391 had little effect on type I IFN signaling. We speculate that the conformation of thioparib might be an important contribution to cellular cytotoxic potency and antitumor immunity. We also extended our off‐target screening to alternative target classes, which indicated only feeble activity at 10 μM against a panel of NAD^+^‐related enzymes and 468 kinases. Importantly, PARP1‐depleted cells exhibited a great decrease in thioparib‐induced DNA damage and marked resistance to thioparib versus parental cells. Finally, we identified PARP1 as one of the top genes that negatively modulates thioparib and Cpd‐391 sensitivity in genome‐scale CRISPR screening, which was consistent with previous reports indicating that the cytotoxicity of PARPi highly depends on the cellular PARP1 activity.

These data suggest that the remarkable cytotoxic properties of thioparib in tumor cells are likely a direct result of its ability to inhibit PARP1. However, the thioparib‐induced type I IFN response may closely depend on PARP7 activity. Previous studies have suggested that PARP7 inhibitors show selective pharmacology in a subset of cancer cell lines(Gozgit *et al*, 2021). As only two cell lines (HT‐29 and MC38) were used to explore the functional relationship between PARP1, 7, and antitumor immunity induced by thioparib, the results need to be confirmed in more models. As thioparib targets multiple PARPs, whether other PARP isoforms are involved in thioparib‐induced cytotoxic effects and antitumor immunity needs to be further determined in the future. In conclusion, we report a highly potent PARP inhibitor thioparib, which significantly inhibits PARylation on DNA and blocks the HR repair of DSBs, subsequently inducing DNA damage checkpoint and type I IFN response. We demonstrated that HR‐deficient cell lines, as well as olaparib‐resistant cells, are hypersensitive to thioparib and demonstrated its superior potency to the clinically approved PARPi in both *in vitro* and *in vivo* models. This study provides a strong rationale for considering thioparib as a new generation of PARP inhibitor in future clinical studies to overcome olaparib resistance.

## Materials and Methods

### Compounds and inhibitors

Simmiparib was synthesized at the Shanghai Institute of Materia Medica, Chinese Academy of Sciences. Olaparib, G007‐LK, VE‐821, chloroquine, losmapimod, RBN‐2397, cisplatin, and LY3214996 were purchased from MedChemExpress (NJ, USA). Talazoparib, veliparib, irinotecan, and rucaparib were purchased from Selleck Chemicals (Shanghai, China). All drugs were dissolved in DMSO and stored at −20°C.

### Synthesis of thioparib and Cpd‐391

The compound 4‐(4‐Fluoro‐3‐(5‐methyl‐3‐(thiazol‐2‐yl)‐5,6,7,8‐tetrahydro‐[1,2,4]triazolo[4,3‐*a*]pyrazine‐7‐carbonyl)benzyl)phthalazin‐1(2*H*)‐one (Cpd‐391) and (*R*)‐4‐(4‐fluoro‐3‐(5‐methyl‐3‐(thiazol‐2‐yl)‐5,6,7,8‐tetrahydro‐[1,2,4]triazolo[4,3‐*a*]pyrazine‐7‐carbonyl)benzyl)phthalazin‐1(2*H*)‐one (thioparib) were synthesized at the Shanghai Institute of Material Medica at the Chinese Academy of Sciences following a similar procedure as described in the Chinese patent, CN201510818057.7 (Zhang *et al*, 2015). High‐performance liquid chromatography (HPLC) analysis confirmed that the purity of both thioparib and Cpd‐391 was ≥ 98%. ^1^H NMR (300 MHz, DMSO‐d6) *δ* 12.61 (s, 1H), 8.25 (d, *J* = 7.7 Hz, 1H), 8.08 (dd, *J* = 11.8, 3.3 Hz, 1H), 7.98–7.80 (m, 3H), 7.63–7.38 (m, 2H), 7.29 (t, *J* = 9.2 Hz, 1H), 5.55 (d, *J* = 17.7 Hz, 0.5H), 5.28 (s, 0.5H), 5.07 (s, 0.5H), 4.90–4.47 (m, 2H), 4.47–4.24 (m, 2H), 3.81 (d, *J* = 12.8 Hz, 0.5H), 3.57 (t, *J* = 15.0 Hz, 1H), 1.41 (d, *J* = 6.4 Hz, 1.5H), 1.15 (s, 1.5H). ^13^C NMR (126 MHz, DMSO‐d6) *δ* 165.6, 165.1, 159.3, 157.4, 155.4, 154.8, 148.6, 148.2, 146.4 (d, *J* = 9.8 Hz), 144.8, 144.1, 135.1, 133.5, 132.2 (d, *J* = 16.9 Hz), 131.5, 129.0, 128.9, 127.9, 126.0, 125.4, 123.1 (d, *J* = 18.1 Hz), 122.7 (d, *J* = 17.8 Hz), 122.0, 116.1 (d, *J* = 21.1 Hz), 50.7, 50.2, 48.6, 48.2, 38.8, 36.4, 19.2, 18.4. HRMS (ESI) calcd. for C_25_H_20_FN_7_O_2_S 501.1383 (M + H)^+^, found 501.1381.

The racemate was subjected to chiral separation through preparative chiral chromatography (Column: CHIRALPAK ID, 0.46 cm I.D. × 15 cm L; mobile phase: CH_2_Cl_2_/MeOH = 90/10) to give Cpd‐391 as the fast‐moving enantiomer and Cpd‐392 (thioparib) as the slower‐moving enantiomer in a ratio of 1:1.

### *In vitro* enzyme assays

The inhibition of the tested compounds on PARP1 and PARP2 enzymatic activity was determined by histone‐based ELISA as reported previously (Ye *et al*, 2013). Briefly, reactions were carried out in 100 μl reaction buffer (10 mM Na_2_HPO_4_, 10 mM NaH_2_PO_4_, 150 mM NaCl, pH 7.4) in 96‐well plates precoated with histone, and a final reaction system containing 10 ng/well‐purified PARP1, 8 μM of NAD^+^, 100 μg/ml of DNA with DMSO or thioparib.

The selective inhibition of the tested compounds on 13 PARP isoforms (including PARP1, PARP2, PARP3, TNKS1, TNKS2, PARP6, PARP7, PARP8, PARP10, PARP11, PARP12, PARP14, and PARP15), as well as NAD^+^‐related enzymes (SIRT1, SIRT2, SIRT3, SIRT5, SIRT6, and CD38), were measured by biotinylated NAD^+^‐based luminescence assays or substrate‐based fluorescence assays by BPS Bioscience (San Diego, CA).

The inhibition of 1 μM thioparib on p38 isoforms, ERK1/2, and MEK1/2 enzymatic activity was determined by competition binding assays as described previously (Lowe *et al*, 2012) by DiscoverX Corporation (Fremont, CA). The inhibition rate (%) was calculated as: [1 − (test compound signal ‐ positive control signal) / (negative control signal − positive control signal)] × 100%.

The DiscoveRx KinomeScan platform was used to detect interactions between thioparib and 468 available kinases.

### Fluorescence anisotropy (FA) PARP1‐DNA binding assays

The effects of the tested compounds on PARP1‐DNA binding were determined by the DSB fluorescence polarization (FA) model as described previously (Chen *et al*, 2019a). Briefly, the binding reactions were carried out in a buffer containing 12 mM HEPES (pH 8.0), 60 mM KCl, 0.12 mM EDTA, 5.5 μM β‐mercaptoethanol, 8 mM MgCl_2_, 0.05 mg/ml BSA and 4% glycerol. Reactions contained a 10 nM DNA probe and 250 nM purified His‐PARP1 protein with DMSO or thioparib. The sequence of the DNA strand used in the assay was 5′‐GGGTTGCGGCCGCTTGGG‐3′ that carried 6‐carboxyfluorescein on the 5′‐terminus, and it was annealed to its complementary DNA strand. The reaction was started with 1 mM NAD^+^ and the data were collected at 60 min with a Synergy H1 microplate reader (BioTek, Winooski, VT).

### Cell lines

Human Jurkat, JeKo‐1, HL‐60, HCT‐15, and U251 cells were purchased from the Shanghai Institutes for Biological Sciences of the Chinese Academy of Sciences. Chinese hamster cell lines V‐C8, V79, and V‐C8 + H13 were gifts from Prof. M. Zdzienicka (Leiden University, Amsterdam, the Netherlands). All other cell lines were from the American‐type culture collection (ATCC; Manassas, VA). All of the cell lines were authenticated by short tandem repeat (STR) testing by GENESKY (Shanghai, China) and tested for mycoplasma with MycAway™‐Color One‐Step mycoplasma detection kit UNG plus (CAT: 40612ES08, Yeasen biotech, Shanghai, China).

Stable knockout cell lines MDA‐MB‐436 53BP1#KO (53BP1^−/−^/BRCA1^−/−^) were generated by the transcription activator‐like effector nuclease (TALEN) technique as described previously (Yang *et al*, 2017). The PARPi‐resistant cell lines Capan‐1/OP, Capan‐1/TP, MDA‐MB‐436/OP, MDA‐MB‐436/TP, U251/OP, and U251/TP were generated by treating cells with increasing concentrations of the PARPi olaparib (OP) or talazoparib (TP) as described previously (Wang *et al*, 2018; Chen *et al*, 2020a).

### Cell viability assays and combination analysis

Briefly, 1,000–8,000 cells per well were seeded in 96‐well plates. After 24 h, cells were treated with indicated drugs for 3 or 7 days and then subjected to the sulforhodamine B (SRB) or CCK8 assays as described previously (Chen *et al*, 2019a). The average IC_50_ values (mean ± SD) were determined with the logit method from three independent tests.

The Combination Index (CI) was calculated by the CompuSyn software following the Chou–Talalay equation (Chou, 2006). CI <1, CI = 1, and CI >1 represented synergism, additive effect, and antagonism, respectively.

### Western blotting

The standard western blotting protocol was conducted to detect the levels of indicated proteins as described previously (Yuan *et al*, 2017; Li *et al*, 2021). Antibody against GAPDH (AF0006) was from Beyotime (Shanghai, China). Antibodies against γ‐H2AX (#2577), CDT1 (#8064), Chk1 (#2360), p‐Chk1(S317, #2344), p‐Chk2 (Thr68, #2661), caspase‐3 (#9662), caspase‐7 (#12827), caspase 9 (#9502), PARP (#9542), Bak (#12105), BID (#2002), Puma (#4976), Noxa (#14766), Bcl‐XL (#2764), XIAP (#14334), MRE11 (#4895), c‐IAP1 (#7943), c‐IAP2 (#3130), STAT1 (#14994), p‐STAT1 (Tyr701, #9167), p‐STAT1 (Ser727, #8826), STAT3 (#9139), p‐STAT3 (Ser727, #9145), STING (#13647), p‐STING (#19781), TBK1 (#3504), p‐TBK1 (#5483), IRF3 (#11904), p‐IRF3 (Ser386, #37829), p38 (#9212), p‐p38 (#9211), ERK1/2 (#9102), p‐ERK1/2 (#4370), MEK1/2 (#4694), p‐MEK1/2 (#9154), JNK (#9252), Axin2 (#2151), KU70 (#4588) and KU80 (#2753) were from Cell Signaling Technology. Antibodies against RPA32 (sc‐271578), Bax (sc‐493), MCL1 (sc‐819), PTIP (sc‐367459), Chk2 (sc‐9604), PARP2 (sc‐30622), XRCC1 (sc‐11429), XRCC3 (sc‐271714), MLH1 (sc‐581), MSH2 (sc‐494), TNKS1/2 (sc‐365897), p‐JNK (sc‐6254), PARP1 (sc‐7150) and PAR [pADPr (10H) (sc‐56198)] were from Santa Cruz Biotechnology (Santa Cruz, CA). Antibody against p‐RPA32 (PLA0071) was from Sigma (Shanghai, China). Antibody against RAD51 (ab63801), CTIP (ab70163), MCRS1/MSP58 (ab247013) and PALB2 (ab202970) were from Abcam. Antibody against MAD2L2/REV7 (BD‐612266) was from BD Biosciences. Antibody against BRCA1 (OP92) and BRCA2 (OP95) were from Millipore. Goat anti‐mouse IgG horseradish peroxidase antibody was provided by Merk/Calbiochem (Darmstadt, Germany). All of the primary antibodies, except for GAPDH, were used after 1:1,000 dilution, and GAPDH primary antibody was used after 1:5,000 dilution. Second antibodies were used following 1:2,000 dilution.

### Comet assays

The OxiSelect™ Comet Assay Kit (Cell Biolabs, CA, USA) was used for comet assays. JeKo‐1 and THP‐1 cells following treatment with indicated drugs for 12 h were collected and assessed the level of DNA damage by quantifying the olive tail moment as described previously (Li *et al*, 2021).

### Cell‐cycle and apoptosis analysis

JeKo‐1 and THP‐1 cells were incubated with indicated drugs for 12 h (for cell‐cycle arrest) or 24 h (for apoptosis) and then analyzed by PI staining‐based or Annexin V‐FITC/PI staining‐based flow cytometry as described previously (Yang *et al*, 2017; Li *et al*, 2021).

### GFP reporter‐based HR repair assays

HR repair assays were performed using the U2OS‐DR‐GFP reporter cell line as described previously (Pierce *et al*, 1999). To measure the repair efficiency, 2 **×** 10^5^ cells were plated in 6‐well plates. Cells were transfected with siRNA for 24 h before I‐SceI transfection. Thioparib or other indicated agents were added at the time of I‐SceI transfection and lasted for 48 h. GFP‐positive cells were collected after being transfected by I‐SceI for 72 h and then quantified by FACSCalibur flow cytometer (BD biosciences, USA).

### RAD51 foci formation assay

Cells were exposed to indicated drugs or DMSO for 48 h before receiving 6 Gy irradiation. After irradiation, cells were incubated for another 6 h and fixed with methanol at −20°C for 20 min. RAD51 foci were examined by staining with a rabbit anti‐RAD51 antibody (Abcam, ab63801) and Goat anti‐Rabbit Alexa Fluor 594 (Invitrogen, A11012). Antibodies were diluted at the ratio of 1:200. Images were acquired using a Leica TCS‐SP8 STED confocal microscope.

### Real‐time quantitative PCR

Cells were plated in 6‐well plates and treated with indicated drugs. The HiPure Total RNA mini kit (MGBio) and PrimeScript™ RT master mix (Takara) were used to extract the total RNA and to reverse transcribe the RNA into cDNA, respectively. Real‐time quantitative PCR was performed with TB green premix EX Taq (Takara). The primer sequences were as follows: 5′‐CCATGGAGAAGGCTGGGG‐3′ (forward) and 5′‐CAAAGTTGTCATGGATGACC‐3′ (reverse) for human *GAPDH*; 5′‐GGAAGCAGCCAAGTCGGTTA‐3′ (forward) and 5′‐TTCACTGAACCTCCCCTGGA‐3′ (reverse) for human *CXCL9*; 5′‐GAACTGTACGCTGTACCTGCA‐3′ (forward) and 5′‐TTGATGGCCTTCGATTCTGGA‐3′ (reverse) for human *CXCL10*; 5′‐TCCATCCAGTGCTACTTGTGT‐3′ (forward) and 5′‐CTGCACTGAAACAGCCCAAAA‐3′ (reverse) for human *IL15*; 5′‐ATGACCAACAAGTGTCTCCTCC‐3′ (forward), 5′‐GGAATCCAAGCAAGTTGTAGCTC‐3′ (reverse) for human *IFNB1*, 5′‐CCACTGAAGCTCCAGAACGAG‐3′ (forward), 5′‐CACTTGAAACTGGGTGCAAAAGA‐3′ (reverse) for human *PARP7*, 5′‐GGTCCCAGCTTAGGTTCATCA‐3′ (forward) and 5′‐CCCAATACGGCCAAATCCGT‐3′ (reverse) for mouse *Gapdh*, and 5′‐AGGGCCAATTACCAGAAGCG‐3′ (forward), 5′‐AGGATGCAAAAGGTCAGTTTGG‐3′ (reverse) for mouse *Parp7*.

### PicoGreen staining assay

PicoGreen staining was performed using Quant‐iT PicoGreen dsDNA Reagent (P7581) supplied by Thermo Fisher Scientific (Shanghai, China). Cells were exposed to indicated drugs or DMSO for 36 h and fixed with 4% polyformaldehyde at 4°C overnight. The PicoGreen staining reagent was added 90 min before harvesting in a dilution of 1:1,000 into the cell culture. After fixation, cells were permeated with 0.5% Triton X‐100 for another 20 min and stained with DAPI. Images were acquired using a Leica TCS‐SP8 STED confocal microscope.

### Immunohistochemistry staining assay

C57BL/6J tumor‐bearing mice were dissected at the end of the experiment and tumor tissues were immediately fixed with 4% polyformaldehyde. The process of paraffin embedding and immunohistochemistry against CD8, CD4, and CD45 was conducted by Shanghai ZuoCheng Bio Company (Shanghai, China). Images were acquired by the same company using a Leica DM6 B microscope equipped with an sCMOS camera (Leica, Wetzlar, Germany).

### Genome‐wide CRISPR‐Cas9 knockout library screening

Lentiviruses containing human CRISPR Knockout Pooled Library (Brunello) (a gift from David Root and John Doench, Addgene#73179) were produced by HEK293T cells and were precipitated by PEG buffer as previously reported (Liu *et al*, 2019; Chen *et al*, 2020b). 5 × 10^8^ Capan‐1/TP cells were infected with virus‐containing library sgRNA at an MOI of 0.4. After being selected by 1 μg/ml puromycin for another 7 days, transfected cells were randomly divided into 4 groups: 2 × 10^7^ cells without further treatment were harvested as the DMSO_Day0_ group. The remaining cells were divided into three groups, 2 × 10^7^ cells receiving 0.1% DMSO for another 14 days termed as DMSO_Day14_ group, 2 × 10^8^ cells treated with 50 nM thioparib (ThP) for another 14 days termed as ThP_Day14_ group, and 2 × 10^8^ cells treated with 50 nM Cpd‐391 for another 14 days termed as 391_Day14_ group. Cells of all four groups were harvested for extracting genomic DNA and amplified by PCR. The PCR products were sequenced by NovoSeq 6000 by Novogene (Tianjin, China). The sequenced data were analyzed by MAGeCK and gene score analysis. CRISPR gene score (CS) = average [log2 (ThP_Day14_ sgRNA abundance/DMSO_Day0_ sgRNA abundance)].

PCR primers used for amplification were as follows: forward primer (P5): AATGATACGGCGACCACCGAGATCTACACTCTTTCCCTACACGACGCTCTTCCGATCTCTTGTGGAAAGGACGAAACACCG; reverse primer (P7): CAAGCAGAAGACGGCATACGAGATGCTGGATTGTGACTGGAGTTCAGACGTGTGCTCTTCCGATCTCCAATTCCCACTCCTTTCAAGACCT.

### Functional pathway enrichment and interaction network analyses

The Kyoto Encyclopedia of Genes and Genomes (KEGG) and Gene Ontology (GO) terms pathway enrichment were conducted by Metascape (https://metascape.org/) (Zhou *et al*, 2019). The min overlap was set to 3, the *P*‐value cutoff was set to 0.01, and the min enrichment was set to 1.5. After that, the top 20 enrichment pathways were visualized using ggplot2 (https://ggplot2.tidyverse.org/). The STRING database (https://cn.string‐db.org/) was used to calculate the human protein interaction network (von Mering *et al*, 2003). The edges were set to indicate both functional and physical protein associations, the line thickness was set to indicate the strength of data support, and the minimum required interaction score was set to high confidence (0.7). The network was then exported and visualized by Cytoscape (https://cytoscape.org/) (Shannon *et al*, 2003).

### RNA interference

siRNA transfection was performed with RNAiMAX transfection reagent (Invitrogen) according to the manufacturer's instructions. The following target sequences were synthesized by GenePharma (Shanghai, China): 5′‐UUCUCCGAACGUGUCACGUTT‐3′ for scrambled siRNA (siNC); 5′‐GCGUGUGAAGAAGAGUAAATT‐3′ for MCRS1#1; 5′‐GCAUAAGUGGCAGGUGCUATT‐3′ for MCRS#2; 5′‐GCAGAGCAACCAAGGAUAATT‐3′ for MCRS1#3;5′‐ GUUGCCUAUGCGCCAAAGA‐3′ for RAD51#1; 5′‐CGGTCAGAGATCATACAGATT‐3′ for RAD51#2.

### Generation of gene knockout cells using CRISPR/Cas9

PARP1, PARP7, STING, and TBK1 single‐gene knockout cells were generated by lentiviral transfection followed by puromycin selection. The pLenti CRISPRv2 vectors inserted with specific sgRNA oligos were transfected together with psPAX2 and pMD2G to product lentiviruses. The sgRNA sequences designed for human *PARP1* knockout were 5′‐CGATGCCTATTACTGCACTG‐3′, 5′‐AGCTAGGCATGATTGACCGC‐3′, and 5′‐CCGGCACCCTGACGTTGAGG‐3′; sgRNA sequence designed for human *PARP7* knockout was 5′‐CACTGAAGCTCCAGAACGAG‐3′; sgRNA sequence designed for human *STING* knockout was 5′‐GGCTGTCACTCACAGGTACC‐3′; sgRNA sequence designed for human *TBK1* knockout was 5′‐AGAGCACTTCTAATCATCTG‐3′; sgRNA sequence designed for mouse *Parp1* knockout was 5′‐ CGAGTGGAGTACGCGAAGAG‐3′; sgRNA sequence designed for mouse *Parp7* knockout was 5′‐AAGGATGCGCTTCTGGTAAT‐3′.

HT‐29 PARP1^−/−^ PARP7^−/−^ double genes knockout cells were conducted by transfecting HT29 PARP7 KO clone cells with lentiviral containing specifically edited pLX‐sgRNA followed by blasticidin selection. The sgRNA sequences designed for *PARP1* knockout was 5′‐ CGATGCCTATTACTGCACTG‐3′.

### *In vivo* anticancer activity experiments

MDA‐MB‐436, Capan‐1, MM1.S, and MV‐4‐11 xenografts were established by inoculating 5 × 10^6^ cells s.c. in female BALB/c nude mice (aged 4–6 weeks), respectively. When the xenografts reached 80–150 mm^3^, mice were randomly assigned to vehicle and treatment groups and received thioparib, talazoparib, olaparib, or vehicle orally once daily for 21 days. Tumor volume and body weight measurements were performed twice a week.

Female M‐NSG mice (Model Organisms, Shanghai) at the age of 7 weeks were injected with 1 × 10^7^ JeKo‐1 cells and randomly assigned to vehicle and treatment groups (*n* = 10 per group). Mice were orally given thioparib, talazoparib, or vehicle once daily for 28 days. The survival rates of the mice were monitored every day. Data were analyzed by log‐rank (Mantel–Cox) test.

Female C57BL/6J mice from Beijing Huafukang Bio Company (Beijing, China) were subcutaneously inoculated with 6 × 10^5^ MC38 cells. When the tumor was palpable, animals were randomly divided into two groups, one received vehicle, and the other receive thioparib 10 mg/kg oral treatment. Treatment lasted for 3 weeks. Tumor volume and body weight measurements were performed twice a week.

All animal experiments were conducted following the Institutional Animal Care and Use Committee guidelines of the Shanghai Institute of Materia Medica. Animals were kept under standard housing conditions in temperature and humidity‐controlled rooms with a maximum of 6 mice in each cage. During the study period, all the mice had free access to irradiation‐sterilized standard laboratory rodent food and sterile water.

PDX model BR‐05‐0028 was derived from a breast cancer patient with BRCA1‐mutated (exon10 C1630T [Q544X]). The assay for anticancer activities of thioparib and olaparib against PDX BR‐05‐0028 was conducted by WuXi AppTec (Shanghai, China) as described previously (He *et al*, 2017; Yuan *et al*, 2017).

### Study design and statistical analyses

For animal experiments, animals were randomly allocated into groups, and measurements of tumor volume were blind to reduce subjective bias. No inclusion or exclusion criteria were conducted in the experiments. For image acquisition in immunofluorescence and immunohistochemical experiments, slides and sections were blind to reduce subjective bias. Statistical analysis was performed by GraphPad Prism. *P*‐values were determined using unpaired *t*‐test, one‐way ANOVA, two‐way ANOVA, and log‐rank (Mantel–Cox) test. Ns: Not significant, *P < 0.05, **P < 0.01, ***P < 0.001. Unless otherwise stated, all experiments were taken from distinct biological replicates with *n* = 3. All data are presented as the mean ± SEM unless otherwise noted.

## Author contributions

**Limin Wang:** Formal analysis; validation; investigation; visualization; methodology; writing – original draft; writing—review and editing. **Pingyuan Wang:** Resources; investigation; methodology. **Xiaomin Chen:** Investigation; methodology. **Hui Yang:** Formal analysis; validation; investigation. **Shanshan Song:** Formal analysis; investigation; methodology. **Zilan Song:** Investigation; methodology. **Li Jia:** Validation; investigation; methodology. **Huadong Chen:** Formal analysis; investigation; methodology. **Xubin Bao:** Formal analysis; investigation. **Ne Guo:** Investigation. **Xiajuan Huan:** Formal analysis; investigation. **Yong Xi:** Investigation. **Yanyan Shen:** Investigation. **Xinying Yang:** Investigation. **Yi Su:** Investigation. **Yiming Sun:** Investigation. **Yinglei Gao:** Investigation. **Yi Chen:** Resources; project administration. **Jian Ding:** Resources; project administration. **Jingyu Lang:** Conceptualization; resources; supervision; methodology; project administration; writing—review and editing. **Ze‐Hong Miao:** Conceptualization; resources; supervision; funding acquisition; project administration; writing—review and editing. **Ao Zhang:** Conceptualization; resources; supervision; project administration; writing—review and editing. **Jinxue He:** Conceptualization; resources; supervision; funding acquisition; visualization; methodology; writing—original draft; project administration; writing—review and editing.

## Disclosure statement and competing interests

The authors declare that they have no conflicts of interest.

## Supporting information



Appendix

Expanded View Figures PDF

Dataset EV1

Source Data for Expanded View

PDF+

Source Data for Figure 4

Source Data for Figure 5

Source Data for Figure 6

## Data Availability

This study includes no data deposited in external repositories.
