# Supplementary material for: Thioparib inhibits homologous recombination repair, activates the type I IFN response, and overcomes olaparib resistance
Source: EMBO Mol Med. 2023 Jan 18;15(3):e16235. doi: 10.15252/emmm.202216235 (PMC9994488; doi:10.15252/emmm.202216235)
Supplement: Supplementary file 1 — Appendix [file EMMM-15-e16235-s002.pdf]

Thioparib Inhibits Homologous Recombination Repair, Activates the Type-I IFN Response, and Overcomes Olaparib Resistance

Li-Min Wang[1,3a], Pingyuan Wang[1,2,5a], Xiao-Min Chen[3,4a], Hui Yang[1,3], Shan-Shan Song[1,3], Zilan Song[2], Li Jia[1,3], Hua-Dong Chen[1,3], Xu-Bin Bao[1,3], Ne Guo[1,3], Xia-Juan Huan[1,3], Yong Xi[1,3], Yan-Yan Shen[1,3], Xin-Ying Yang[1,3], Yi Su[1,3], Yi-Ming Sun[1,3], Ying-Lei Gao[1,3], Yi Chen[1,3], Jian Ding[1,3], Jing-Yu Lang[3,4*], Ze-Hong Miao[1,3*], Ao-Zhang[1,2,3*], Jin-Xue He[1,3*]

Table of Content

Appendix Information	
**Appendix Fig. S1**	**Effects of thioparib on DNA damage, cell cycle, and apoptosis**
**Appendix Fig. S2**	**Effects of thioparib on Axin2 stabilization and type I IFN signaling**
**Appendix Fig. S3**	**Immunohistochemistry staining of CD4, CD8, and CD45 expression in PARP1/7 knockout MC38 tumor tissue sections**
**Appendix Fig. S4**	**KEGG pathway analysis of the top candidates in thioparib and/or Cpd-391 treated groups**
**Appendix Table S1**	**Summary of thioparib *in vitro* activities**
**Appendix Table S2**	**PARPi' cytotoxicity in HR-deficient cells**
**Appendix Table S3**	**Antiproliferation activity of thioparib in cancer cells harboring deficient BRCA1, BRCA2, PTEN, or EWS-FL11**
**Appendix Table S4**	**Antiproliferation activity of thioparib in PARPi-resistant cell lines**
**Appendix Table S5**	**Antiproliferation activity of thioparib in the HR-deficient hematologic cancer cell lines**
**Appendix Table S6**	**Antiproliferation activity of thioparib in the PARP1 knockout (KO) cell lines**

Appendix Figures

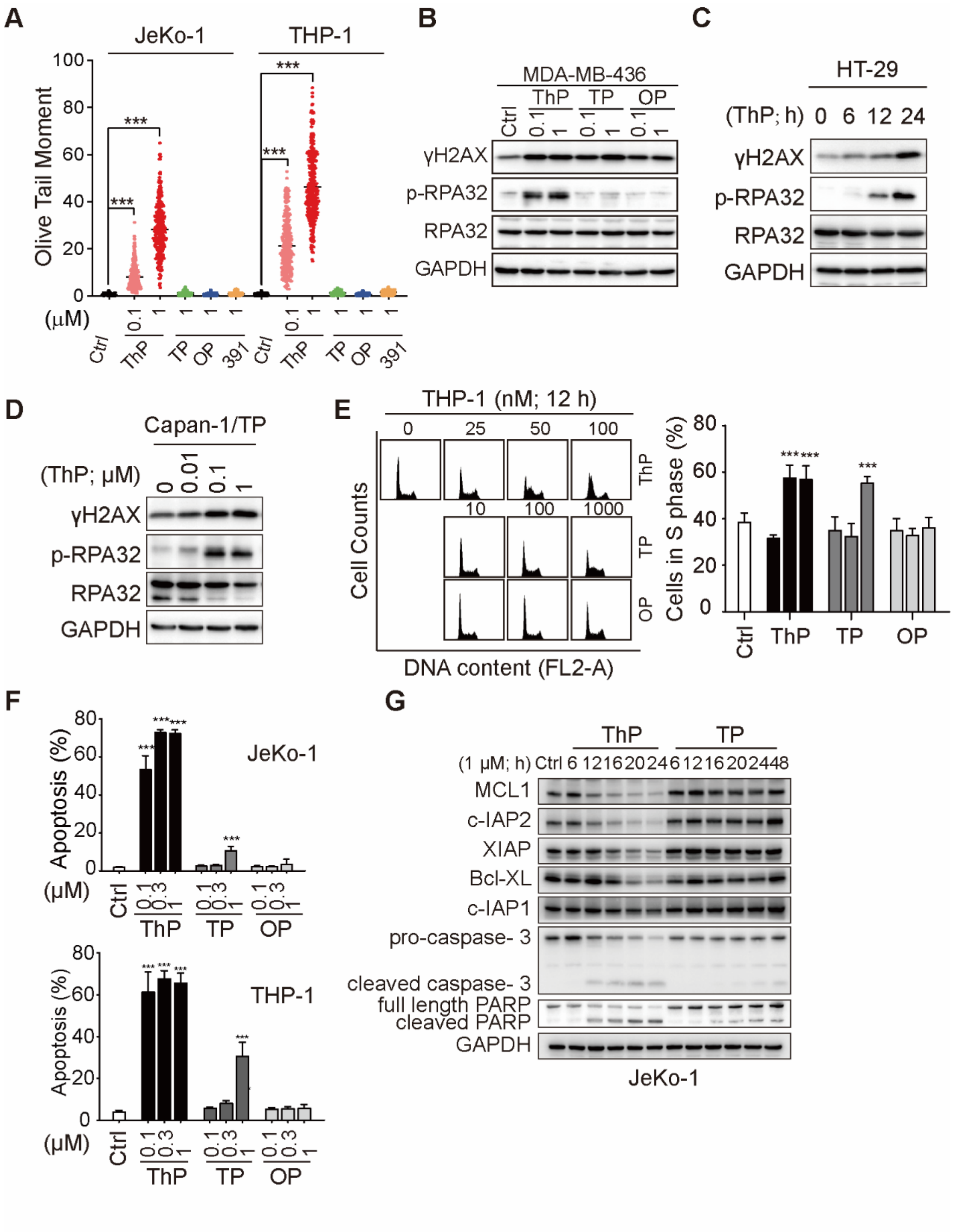

Appendix Fig.S1 Wang LM, et.al

Appendix Fig. S1. Effects of thioparib on DNA damage, cell cycle, and apoptosis. (**A**) Measurement of DNA damage in JeKo-1 and THP-1 cells following the indicated PARP inhibitor treatment, as subjected to comet assay. Olive tail moments of at least 100 cells from each group were quantified using CASP software. Data from three independent experiments were analyzed by one-way ANOVA. ***P < 0.0001. (**B-D**) Western blotting of γH2AX and p-RPA32 in MDA-MB-436 (**B**), HT-29 (**C**), and PARPi-resistant Capan-1/TP (**D**) cells after treatment with 1 μM or the indicated concentration of thioparib (ThP) for 24 h or the indicated time. (**E**) The S phase arrest in THP-1 cells following thioparib treatment. Cells were treated with the indicated PARPi for 12 h and then analyzed by PI staining-based flow cytometry. The percentage of cells in the S-phase (%S) is shown in the right panel**.** Data from three independent experiments were analyzed by two-way ANOVA. Left to right: ***P < 0.0001, < 0.0001, = 0.0002. (**F**) Cell apoptosis induced by thioparib in JeKo-1 and THP-1 cells was analyzed by Annexin V–FITC-based flow cytometry. Data are expressed as the mean ± SD. Data from three independent experiments were analyzed by two-way ANOVA. Upper panel: ***P < 0.0001, < 0.0001, < 0.0001, = 0.0005, (from left to right); lower panel: ***P < 0.0001. (**G**) The changes in protein levels of antiapoptotic proteins in JeKo-1 cells following thioparib or talazoparib treatment for the indicated time. ThP: Thioparib, OP: Olaparib, TP: Talazoparib, 391: Cpd-391.

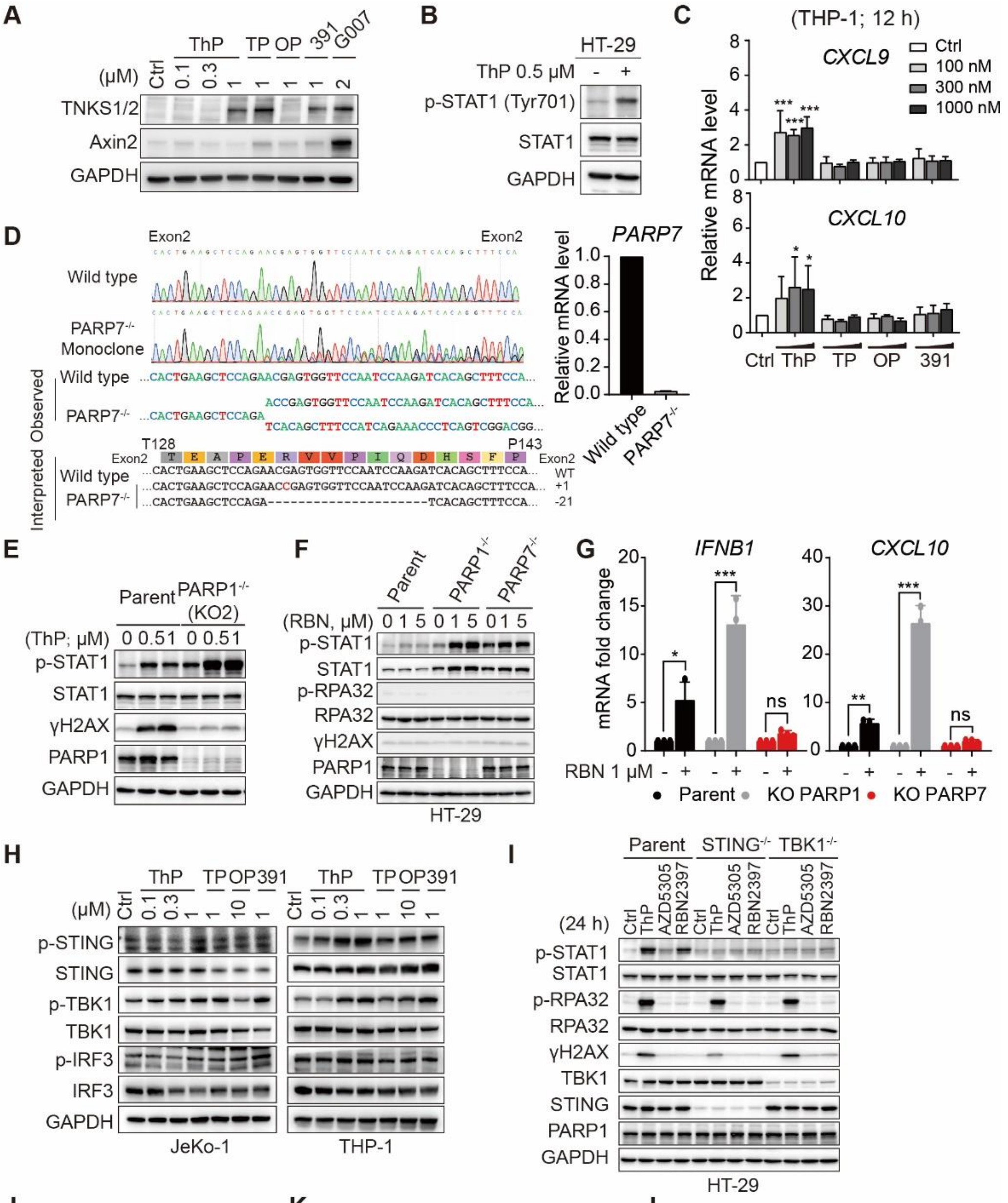

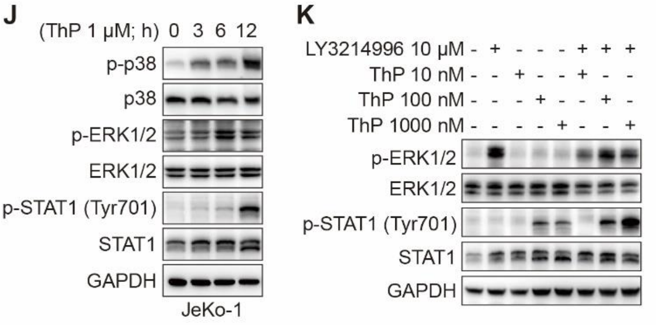

L

Target	Inhibition rate (%)<br>1 μM Thioparib
p38α	6
p38β	15
p38δ	41
p38γ	21
ERK1	7
ERK2	10
MEK1	5
MEK2	3

Appendix Fig.S2 Wang LM, et.al

Appendix Fig. S2. Effects of thioparib on Axin2 stabilization and type I IFN signaling. (**A**) Effects of thioparib on the protein levels of TNKS1/2 and Axin2 in SW480 cells. Cells were treated with the indicated drugs for 48 h and then subjected to western blotting. (**B**) Protein levels of p-STAT1 in HT-29 cells after treatment with 0.5 μM thioparib for 24 h, as determined by western blotting. (**C**) Effects of thioparib on *CXCL9* and *CXCL10* mRNA levels in THP-1 cells. Data are expressed as the mean ± SD from three independent experiments. Data were analyzed by two-way ANOVA. Upper panel: ***$P < 0.0001$, = 0.0002, < 0.0001; lower panel: *$P = 0.0148$, 0.0245 (from left to right). (**D**) Sequencing and RT-qPCR analysis of CRISPR/Cas9-mediated *PARP7* knockout HT-29 cells. Nucleotide sequences of the *PARP7* gene at the target site in the wild-type and $PARP7^{-/-}$ monoclonal cells were shown in the left panel. RT-qPCR detection of *PARP7* mRNA in wild type and KO cells was shown in the right panel. (**E**) Protein levels of p-STAT1, STAT1, γH2AX, and PARP1 in HT-29 parent and PARP1 knockout (#KO2) cells. (**F**) Protein levels of p-STAT1, γH2AX, and p-RPA32 in HT-29 parent, $PARP1^{-/-}$, and $PARP7^{-/-}$ cells after treatment with the PARP7 inhibitor RBN-2397 for 24 h. (**G**) The changes in the mRNA levels of *IFNB1* and *CXCL10* in HT-29 parent, $PARP1^{-/-}$, and $PARP7^{-/-}$ cells following RBN-2397 treatment. Data are expressed as the mean ± SD from three independent experiments and were analyzed by two-way ANOVA. Ns: not significant. Left panel: *$P = 0.0112$, ***$P < 0.0001$, ns: $P = 0.8867$; right panel: **$P = 0.0086$, ***$P < 0.0001$, ns: $P = 0.8208$. (**H**) Effects of thioparib on STING pathway determined by western blotting in JeKo-1 and THP-1 cells following the indicated drug treatment. (**I**) Western blotting

analysis of p-STAT1, p-RPA32, and γH2AX in HT-29 parent, STING$^{-/-}$, and TBK1$^{-/-}$cells following PARP1-selective inhibitor AZD5305 and PARP7 inhibitor RBN-2397 treatment. (**J**) The changes in the protein levels of p-p38, p-ERK1/2, and p-STAT1 in JeKo-1 cells after treatment with thioparib for the indicated time. (**K**) The changes in the protein levels of p-STAT1 in JeKo-1 cells after treatment with thioparib in combination with ERK inhibitor LY3214996. (**L**) A table summarizing the percentage of enzyme inhibition by 1 μM thioparib for the p38 MAPK isoforms, ERK1/2, and MEK1/2. Data were from Fig. EV1B. ThP: Thioparib, OP: Olaparib, TP: Talazoparib, 391: Cpd-391.

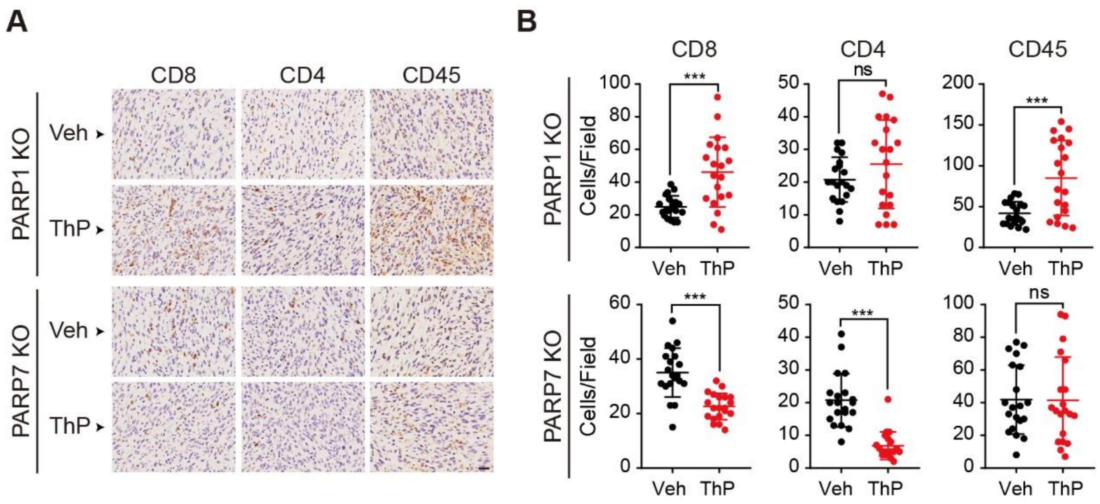

Appendix Fig.3 Wang LM, et.al

Appendix Fig. S3. Immunohistochemistry staining of CD4, CD8, and CD45 expression in PARP1/7 knockout MC38 tumor tissue sections. (**A**) Representative images of PARP1 and PARP7 knockout MC38 tumor tissue sections that were collected after vehicle or thioparib treatment for three weeks and immunostained with the indicated markers. Scale bar: 25 μm. (**B**) Quantification of cells positive for CD4, CD8, and CD45 per field was shown. Statistical analysis was performed by unpaired t-test. Upper panel: ns: $P = 0.1691$, ***$P = 0.0002$, 0.0003 (from left to right); lower panel: ns: $P = 0.9528$, ***$P < 0.0001$.

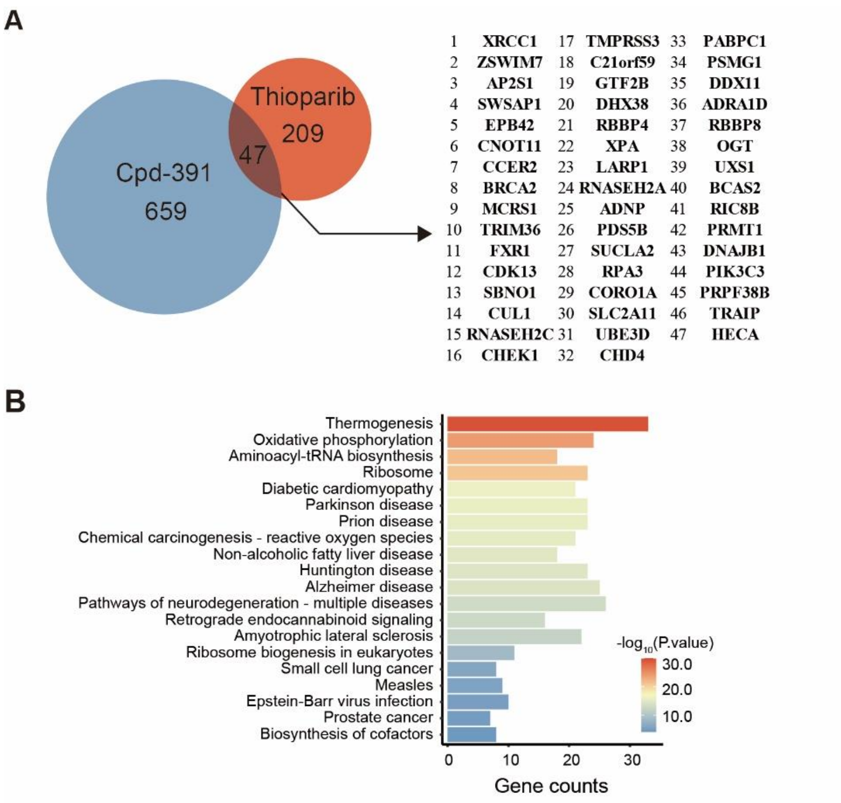

Appendix Fig.S4 Wang LM, et.al

Appendix Fig. S4. KEGG pathway analysis of the top candidates in thioparib and/or Cpd-391 treated groups. (**A**) Venn diagram of the sensitive gene candidates in thioparib and Cpd-391 groups with ≥ 3 sgRNA hits, p-value ≤ 0.05, and log fold change ≤ −0.5 (left panel); and the list of 47 common genes between the two groups are shown in the right panel. (**B**) The top 20 enrichment in the KEGG pathway analysis of gene candidates (with ≥ 3 sgRNA hits, p-value ≤ 0.01, and log fold change ≥ 1) whose disruptions lead to thioparib resistance.

Appendix Tables

Appendix Table S1. Summary of thioparib *in vitro* activities.

	Mean ± SD (nM)			
	**Thioparib**	**Cpd-391**	**Talazoparib**	**Olaparib**
PARP1 EC_{50}	0.13 ± 0.02	0.26 ± 0.05	1.66 ± 0.14	3.91 ± 0.07
PARP2 EC_{50}	0.006 ± 0.005	0.010 ± 0.002	0.61 ± 0.26	0.94 ± 0.40
DSB-FA IC_{50}	25.05 ± 9.89	58.01 ± 26.48	41.03 ± 9.75	592.65 ± 206

Appendix Table S2. PARPi' cytotoxicity in HR-deficient cells.

**No.**	**Cells**	**Mutations**	**TP**[ab]	**OP**[ab]	**RP**[b]	**VP**[b]	**ThP**[a]
**1**	**HCC1395**	*BRCA1*$^{-/-}$	3	8	ND	200	ND
**2**	**MDA-MB-468**	*BRCA1*$^{-/-}$	1	200	ND	4800	ND
**3**	**HCC1937**	*BRCA1*$^{-/-}$	125	8130	ND	39400	0.88
**4**	**MX-1**	*BRCA1*$^{-/-}$	0.3	23.2	5.3	ND	ND
**5**	**SUM149**	*BRCA1*$^{-/-}$	0.01	19.8	7.9	818	ND
**6**	**UWB1.289**	*BRCA1*$^{-/-}$	2.59	1270	ND	ND	0.27
**7**	**MDA-MB-436**	*BRCA1*$^{-/-}$	0.33	19.77	3000	30	0.05
**8**	**DoTC2-4510**	*BRCA2*$^{-/-}$	ND	890	ND	ND	ND
**9**	**V-C8**	*BRCA2*$^{-/-}$	1.28	236	ND	ND	0.96
**10**	**Capan-1**	*BRCA2*$^{-/-}$	0.02	1980	609	ND	0.27
**11**	**MB-468**	*PTEN*$^{-/-}$	6	368	220	ND	ND
**12**	**LNCap**	*PTEN*$^{-/-}$	3	589	737	ND	ND
**13**	**PC-3**	*PTEN*$^{-/-}$	383	13130	293	ND	1.64
**14**	**U251**	*PTEN*$^{-/-}$	2368	6040	4205	ND	0.63
**15**	**SK-ES-1**[a]	*EWS-FLI1*	17.71	3720	6782	ND	2.66
**16**	**RD-ES**[a]	*EWS-FLI1*	26.34	2040	3016	11826	1.27
	**Mean (nM)**	-	**195**	**2416**	**1887**	**9512**	**0.96**
	**SD**	-	609	3709	2289	15317	0.82

TP: Talazoparib, OP: Olaparib, RP: Rucaparib, VP: Veliparib, ThP: Thioparib

ND: Not detected

[a]Data are from Supplementary Table 3

[b]Reference data (Chen et al, 2019a)

Appendix Table S3. Antiproliferation activity of thioparib in cancer cells harboring deficient BRCA1, BRCA2, PTEN, or EWS-FL11.

cell lines	Mutations	IC_{50}(mean ± SD)（nM）			
		Thioparib	Cpd-391	Talazoparib	Olaparib
MDA-MB-436	*BRCA1*$^{-/-}$	0.05 ± 0.01	0.12 ± 0.06	0.33 ± 0.28	19.77 ± 1.89
UWB1.289	*BRCA1*$^{-/-}$	0.27 ± 0.05	0.51 ± 0.26	2.59 ± 0.55	1270 ± 270
HCC1937	*BRCA1*$^{-/-}$	0.88 ± 0.17	7.15 ± 2.59	125 ± 14	8130 ± 1800
V-C8	*BRCA2*$^{-/-}$	0.96 ± 0.78	3.5 ± 2.50	1.28 ± 0.31	236 ± 11
Capan-1	*BRCA2*$^{-/-}$	0.27 ± 0.05	0.70 ± 0.24	0.02 ±0.01	1980 ± 110
U251	*PTEN*$^{-/-}$	0.63 ± 0.07	15.52 ± 0.93	2368 ± 281	6040 ± 1900
PC-3	*PTEN*$^{-/-}$	1.64 ± 0.18	390 ± 66	383 ± 175	13130 ± 1030
RD-ES-1	*EWS-FLI1*	1.27 ± 0.42	6.81 ± 2.76	26.34 ± 7.95	2040 ± 20
SK-ES-1	*EWS-FLI1*	2.66 ± 0.49	7.56 ± 3.05	17.71 ± 6.41	3720 ± 330
**Average**		**0.96**	**47.99**	**325**	**4063**

Appendix Table S4. Antiproliferation activity of thioparib in PARPi-resistant cell lines.

Cell lines	IC_{50}(mean ± SD) （nM）			
	Thioparib	Cpd-391	Talazoparib	Olaparib
Capan-1/OP	2.40 ± 0.18	198 ± 10	328 ±72	29470 ± 8140
Capan-1/TP	1.49 ± 0.17	53.36 ± 3.39	146 ± 26	10860 ± 410
MDA-MB-436/OP	0.49 ± 0.31	269 ± 96	1715 ± 452	4460 ±2000
MDA-MB-436/TP	11.08 ± 3.27	344 ± 55	13980 ± 516	12460 ± 1560
U251/OP	2.82 ± 0.63	2321 ± 365	13207 ± 3060	38290 ± 8550
U251/TP	2.07 ± 0.28	1122 ± 93	44802 ± 63	25980 ± 6670
**Average**	**3.39**	**718**	**12363**	**20253**

Appendix Table S5. Antiproliferation activity of thioparib in the HR-deficient hematologic cancer cell lines.

Cell lines	IC_{50}(mean ± SD) （nM）			
	Thioparib	Cpd-391	Talazoparib	Olaparib
Jurkat	6.38 ± 0.90	9637 ± 848	3366 ± 395	17660 ± 1120
SU-DHL-1	8.81 ± 1.22	28.67 ± 7.59	20.33 ± 6.13	13020 ± 2270
JeKo-1	10.01 ± 1.29	162 ± 31	439 ± 65	8920 ± 600
MM.1R	8.55 ± 1.06	46.33 ± 2.87	128 ± 23	9310 ± 2320
MM.1S	12.02 ± 2.66	1016 ± 319	2100 ± 566	27500 ± 1430
NCI-H929	12.88 ± 1.85	145 ± 44	319 ± 86	19130 ± 2240
MV-4-11	6.44 ± 0.73	10.67 ± 4.11	196 ± 37	2210 ± 260
HL-60	12.46 ± 0.59	286± 34	402 ± 117	5980 ± 840
THP-1	11.81 ± 0.55	3441 ± 621	2098 ± 151	15300 ± 1340
NOMO-1	19.33 ± 6.13	12060 ± 2474	3363 ± 930	> 100000
KG-1	119 ± 13	18721 ± 5977	10865 ± 1912	42880 ± 6430
K-562	79.71 ± 35.10	25463 ± 5000	2673 ± 516	48100 ± 9100
**Average**	**26**	**5918**	**2164**	**25834**

Appendix Table S6. Antiproliferation activity of thioparib in the PARP1 knockout (KO) cell lines.

Cells	Gene Deficiency	IC_{50} (nM)			
		Thioparib	Cpd-391	Talazoparib	Olaparib
HT-29		11.43 ± 1.58	57.57 ± 15.60	28.01 ± 7.21	11960 ± 2900
HT-29 KO1	POLQ	71.14 ± 15.05	416.09 ± 68.07	2872.99 ± 749.04	61340 ± 26800
HT-29 KO2		45.53 ± 3.32	611.66 ± 329.37	1248.58 ± 112.53	34230 ± 5200
Capan-1/TP		12.56 ± 1.01	841.44 ± 60.93	1016.43 ± 125.54	38310 ± 16230
Capan-1/TP KO1	BRCA2	145.94 ± 10.04	48303.62 ± 1248.57	> 100000	181440 ± 5150
Capan-1/TP KO2		149.21 ± 18.18	48452.24 ± 5900.39	> 100000	170220 ± 15300
