# Supplementary material for: Thioparib inhibits homologous recombination repair, activates the type I IFN response, and overcomes olaparib resistance
Source: EMBO Mol Med. 2023 Jan 18;15(3):e16235. doi: 10.15252/emmm.202216235 (PMC9994488; doi:10.15252/emmm.202216235)
Supplement: Supplementary file 2 — Expanded View Figures PDF [file EMMM-15-e16235-s004.pdf]

Expanded View Figures

Figure EV1. Effects of thioparib on NAD^+-related or kinase enzymes.

A Concentration-response curves of thioparib against NAD^+-related enzymes SIRT1, SIRT2, SIRT3, SIRT5, SIRT6, and CD38 using *in vitro* enzymatic assays. Suramin, Nicotinamide, and Apigenin are used as the positive controls. Data from two technical replicates are shown as mean ± SEM.

B Kinase selectivity of 1 μM thioparib in the DiscoveRx KINOMEScan screening platform. Left panel, TREEspot interaction maps for thioparib in 468 kinase targets. Right panel, a table summarizing the percentage of enzyme inhibition by 1 μM thioparib, and the IC_{50} values of niraparib and rucaparib for the off-targets DYRK1B, CDK16, PIM3, and DYRK1A from published data.

C Thioparib sensitivity of V79 (BRCA2 wild-type), V-C8 (BRCA2 null), and V-C8 + H13 cells (with BRCA2 restored) in SRB assay. Data from three independent experiments are presented as mean ± SEM.

© 2023 The Authors

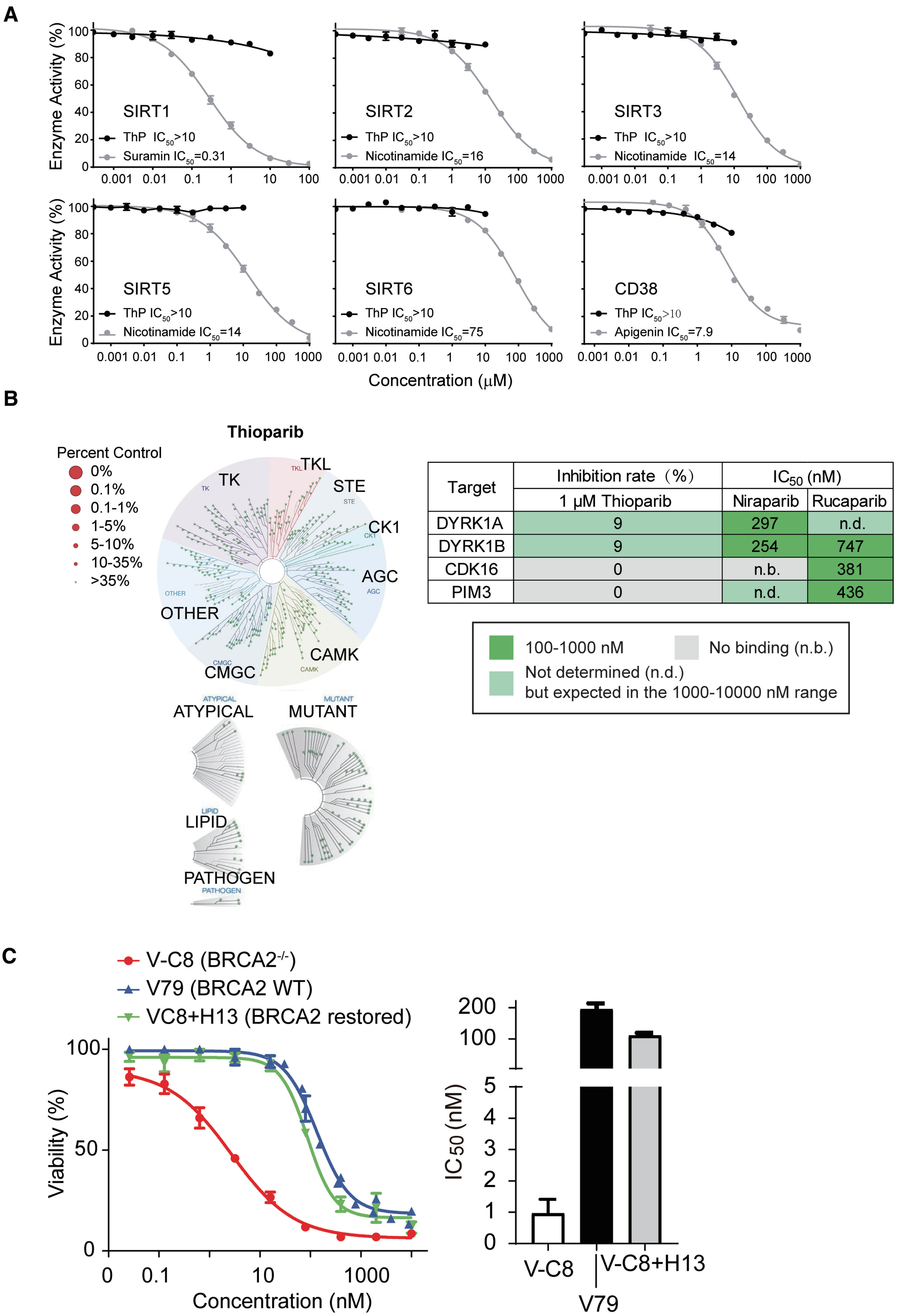

Figure EV1.

 © 2023 The Authors

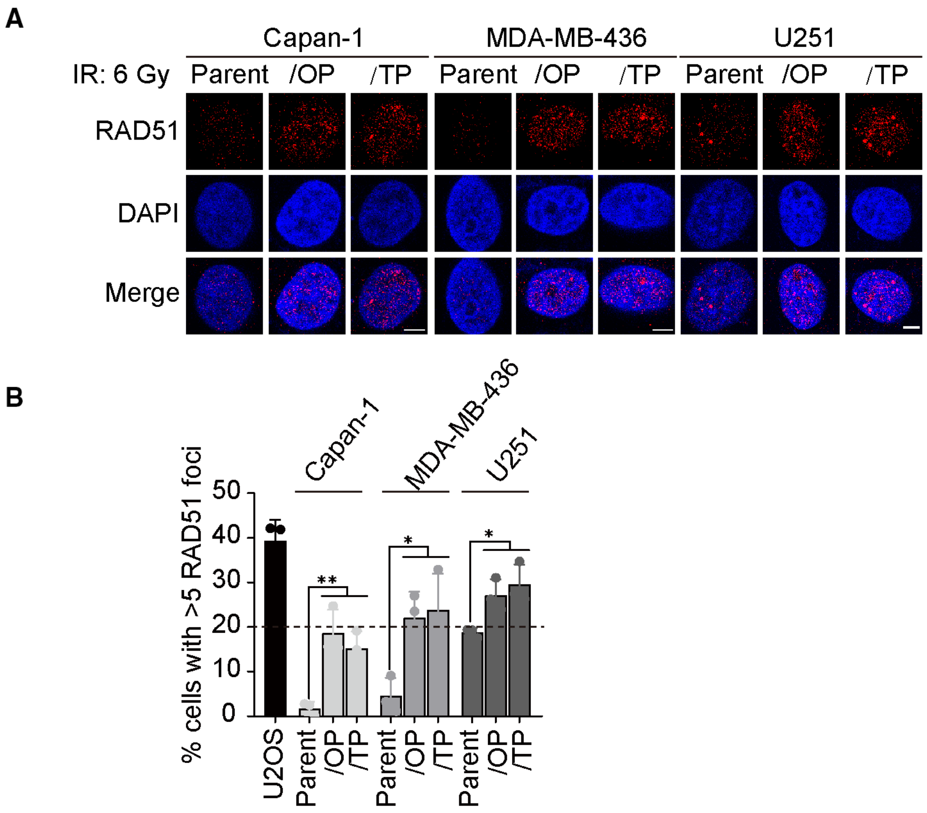

Figure EV2. Restoration of RAD51 foci formation in a panel of cell lines with acquired PARPi resistance.

A Immunofluorescence staining of RAD51 foci induced by 6 Gy irradiation in PARPi-resistant clones Capan-1/OP, Capan-1/TP, MDA-MB-436/OP, MDA-MB-436/TP, U251/OP, U251/TP, and their parental cells. Representative pictures are shown. Scale bar: 5 μm.

B Quantification of RAD51-positive cells with ≥5 foci. HR-proficient U2OS cells were used as the positive control (data from Fig 5C). Data represent the mean ± SD. Data from three independent experiments were analyzed by one-way ANOVA. $^{**}P = 0.0032$, $P = 0.0093$, $^{*}P = 0.0247$, $P = 0.0170$, $P = 0.0433$, $P = 0.0153$ (from left to right).

© 2023 The Authors

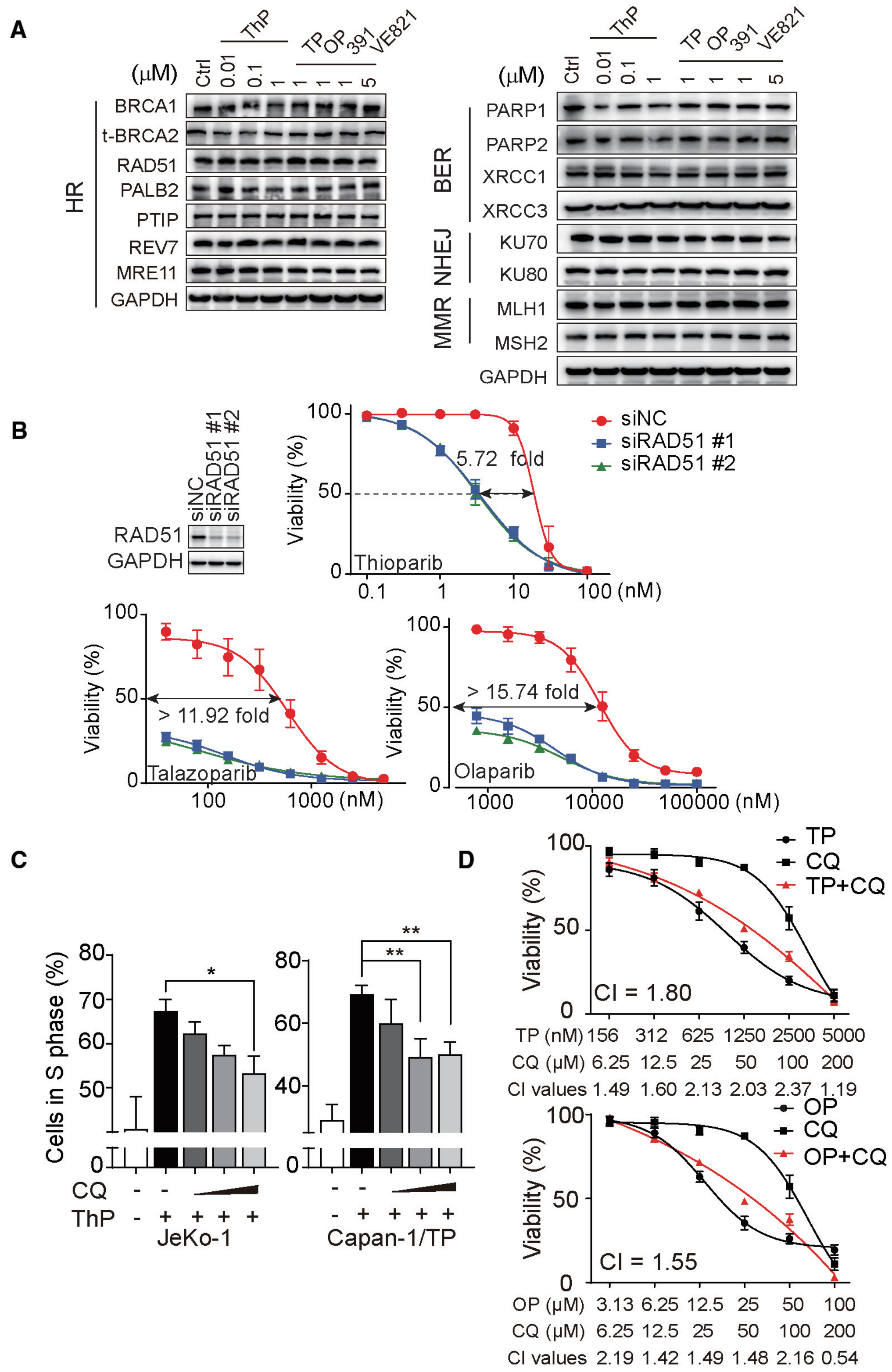

Figure EV3.

© 2023 The Authors

Figure EV3. Effects of thioparib on DNA damage-related proteins and the combination effects with chloroquine.

A The changes in protein levels of DNA repair-related proteins [including HR (left panel), BER, NHEJ, and MMR (right panel) pathways] following thioparib or the indicated drug treatment for 12 h.

B Depletion of RAD51 sensitized Capan-1/TP cells to thioparib, olaparib, and talazoparib. Data from three biological replicates are shown as mean ± SEM.

C The percentage of cells in the S phase in JeKo-1 and Capan-1/TP cells treated with thioparib and chloroquine alone or in combination. The cell cycle was analyzed by PI staining-based flow cytometry. Data are shown as the mean ± SD. Data from three independent experiments were analyzed by one-way ANOVA. Left to right: $*P = 0.0139$, $**P = 0.0072$, $P = 0.0093$.

D The effect of chloroquine and the indicated PARP inhibitor combination on the viability of JeKo-1 cells. Cells were pretreated with chloroquine for 4 h followed by talazoparib or olaparib treatment for 3 days and subjected to CCK-8 assay. Average CI values from three independent experiments were presented. Data are expressed as the mean ± SEM.

Data information: ThP, thioparib; OP, olaparib; TP, talazoparib; 391, Cpd-391; CQ, chloroquine.
Source data are available online for this figure.

Figure EV4. Effects of thioparib on p38 MAPK signaling.

A *IFN-β* and *CXCL10* mRNA levels in HT29 PARP1$^{-/-}$ or PARP7$^{-/-}$ cells. Statistical analysis was performed by one-way ANOVA. ns: $P = 0.1558$, $**P = 0.0075$, $*P = 0.0225$, $***P = 0.0003$. Mean ± SD of three independent experiments is shown. Data were from Fig 6E.

B Effects of thioparib on MAPK signaling determined by western blotting in JeKo-1 and THP-1 cells, followed by PARPi treatment for 12 h.

C Inhibition of p38 activity with losmapimod prevents thioparib-induced p-STAT1 in JeKo-1 and THP-1 cells.

D, E Inhibition of p38 activity with losmapimod reverses the increase in *IFNB1* and *CXCL10* mRNA levels induced by thioparib in JeKo-1 (D) and HT-29 PARP7$^{-/-}$ (E) cells. Cells were pretreated with 10 μM losmapimod for 4 h, followed by thioparib treatment. Data are shown as the mean ± SD from three independent experiments. Statistical analysis was performed by one-way ANOVA. Left panel: ns: $P = 0.7380$, $P > 0.9999$, $***P < 0.0001$, $P = 0.0009$; right panel: ns: $P = 0.8173$, $P = 0.1596$, $*P = 0.0301$, $**P = 0.0036$ (from left to right).

F The activation of p38 by thioparib is abolished by *PARP1* depletion. The wild-type and *PARP1* knockout THP-1 cells were treated with the indicated concentrations of thioparib and subjected to western blotting with the indicated antibodies.

G Phosphorylation of p38 MAPK is associated with increased DNA damage. JeKo-1 cells were treated with the indicated agents, and p-p38 and γH2AX were detected using western blotting.

H Immunofluorescence microscopic analysis of cytosolic dsDNA in wild type, PARP1$^{-/-}$ and PARP7$^{-/-}$ HT-29 cells treated with or without thioparib (1 μM for 36 h). Left, representative images of PicoGreen (green; dsDNA) and DAPI (blue; nucleus) staining. The yellow arrowheads indicate the occurrence of cytosolic dsDNA. Scale bar: 5 μm. Right, the graph shows the percentage of cells with cytosolic dsDNA ($n > 100$ cells each) from three biological replicates. Data are shown as the mean ± SD. Statistical analysis was performed by two-way ANOVA. $***P < 0.0001$, $P = 0.0005$ (from left to right), ns$P = 0.9065$.

I The protein levels of p-STAT1, p-RPA32, and γH2AX in HT-29 parent, PARP1, 7 single or double knockout cells after thioparib treatment.

J The changes in *IFNB1* and *CXCL10* mRNA levels in HT-29 parent, PARP1, 7 single or double KO cells exposed to thioparib. Data from three independent experiments are shown as the mean ± SD. Statistical analysis was performed by two-way ANOVA. Upper panel: the absolute mRNA levels by normalizing against the parent untreated group, and *P*-value was calculated compared with the parent untreated group, $***P = 0.0004$, $P = 0.0003$, $P = 0.0001$, $P = 0.0004$ (from left to right). Lower panel: the relative mRNA fold change normalized against the untreated group in each cell line, and *P*-value was calculated compared with the untreated group in each cell line, ns: $P = 0.5233$, $P > 0.9999$, $P = 0.6815$, $P > 0.9999$, $***P < 0.0001$, $P = 0.0002$, $P < 0.0001$, $P < 0.0001$ (from left to right).

Data information: ThP, thioparib; OP, olaparib; TP, talazoparib; 391, Cpd-391; Los, losmapimod; SP, simmiparib; CPT, irinotecan.
Source data are available online for this figure.

© 2023 The Authors

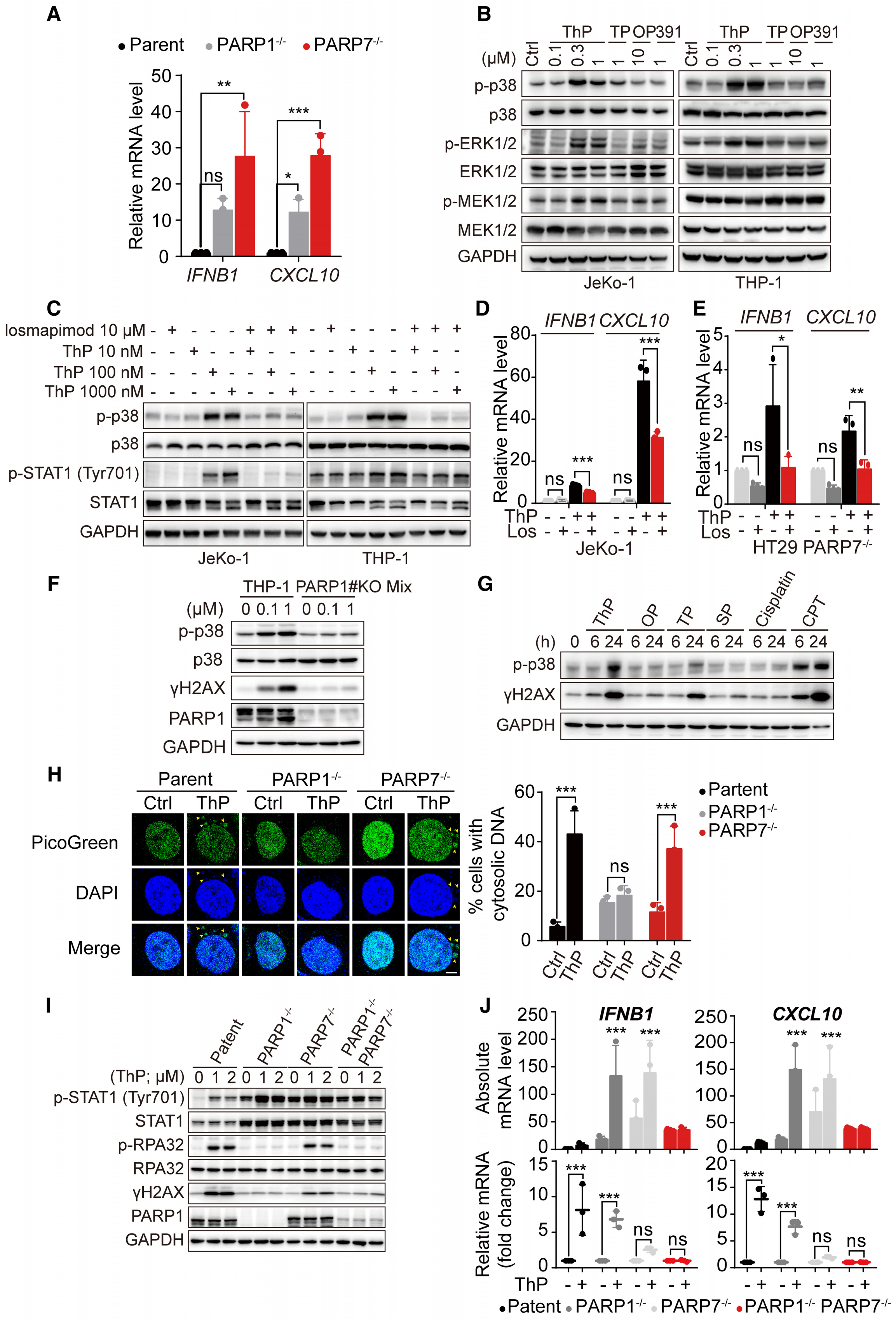

Figure EV4.

© 2023 The Authors

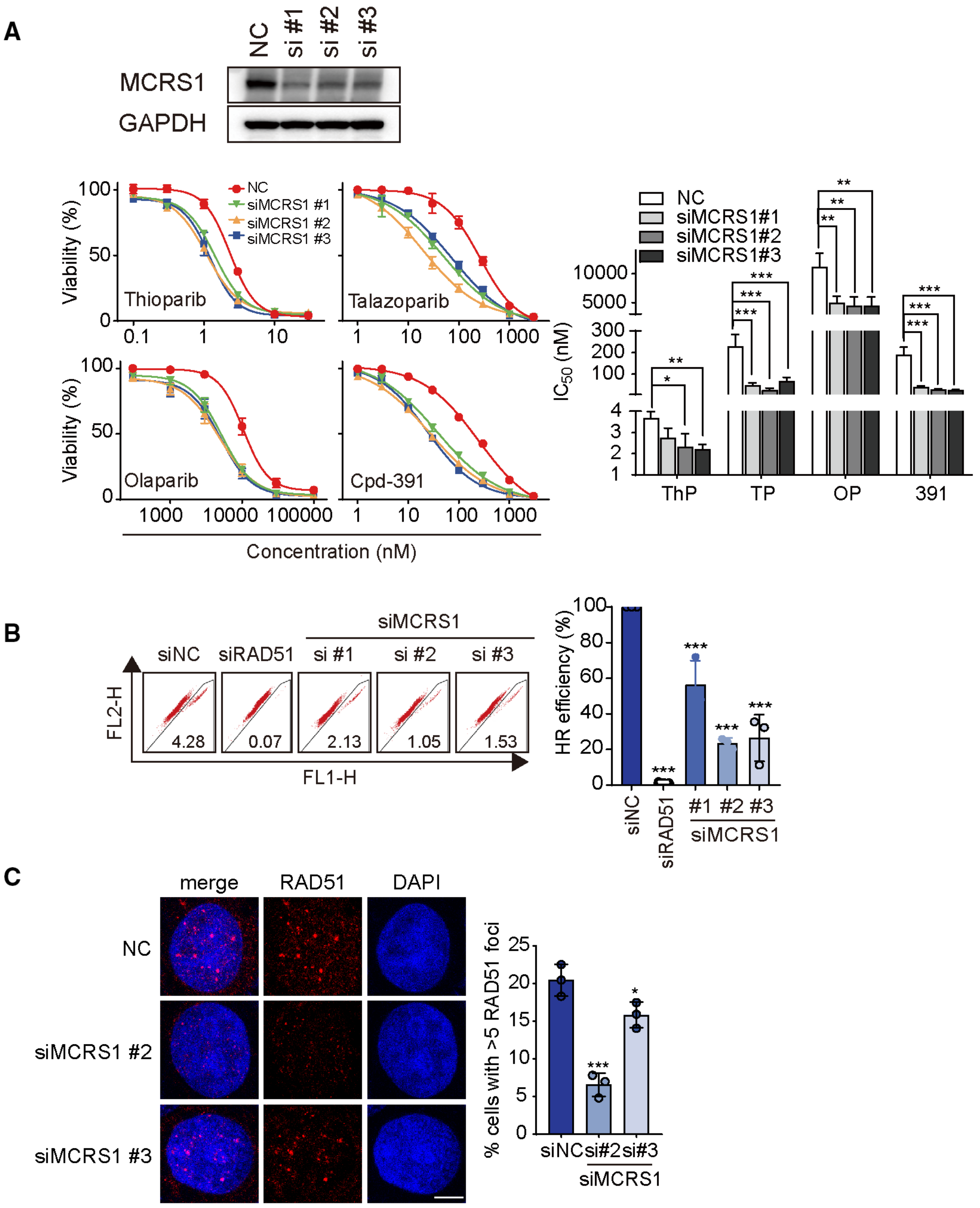

Figure EV5. MCRS1 depletion enhances PARPi sensitivity due to defective HR.

A MCRS1 knockdown increased the sensitivity of Capan-1/TP cells to the indicated PARPi. Statistical analysis was performed by one-way ANOVA. Left to right: $P = 0.0146$, $P = 0.0096$, $P = 0.0002$, $P = 0.0001$, $P = 0.0005$, $P = 0.0050$, $P = 0.0029$, $P = 0.0030$, $P < 0.0001$, $P < 0.0001$, $P < 0.0001$. In the left panel, data from three independent experiments are presented as mean ± SEM. In the right panel, IC_{50} values from the same experiments are shown as mean ± SD.

B HR-mediated DNA repair is impaired in MCRS1-depleted U2OS-DR-GFP cells. U2OS-DR-GFP reporter cells were transfected with siRNAs targeting MCRS1, RAD51 (positives control), or negative control for 24 h, followed by I-SceI transfection. Data from three biological replicates are shown as the mean ± SD. Statistical analysis was performed by one-way ANOVA. Left to right: ***$P < 0.0001$, $P = 0.0003$, $P < 0.0001$, $P < 0.0001$.

C Reduced RAD51 foci formation in MCRS1-depleted Capan-1/TP cells after 6 Gy of ionizing radiation (IR) exposure. Graphs (right panel) show the quantification of cells with ≥ 5 RAD51 foci. Scale bar: 5 μm. Data represent the mean ± SD. Data from three independent experiments were analyzed by one-way ANOVA, ***$P = 0.0002$, *$P = 0.0353$. ThP, thioparib; OP, olaparib; TP, talazoparib; 391, Cpd-391.

Source data are available online for this figure.

© 2023 The Authors
