# Supplementary material for: Thioparib inhibits homologous recombination repair, activates the type I IFN response, and overcomes olaparib resistance
Source: EMBO Mol Med. 2023 Jan 18;15(3):e16235. doi: 10.15252/emmm.202216235 (PMC9994488; doi:10.15252/emmm.202216235)

Figure EV4B left panel (JeKo-1)

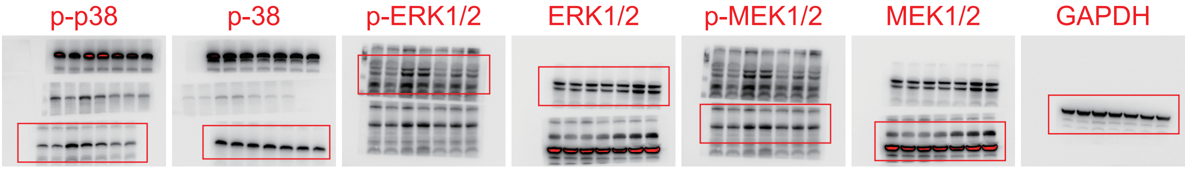

Figure EV4B right panel (THP-1)

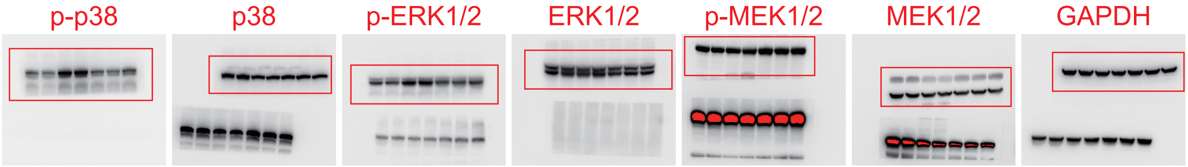

Figure EV4C left panel (JeKo-1)

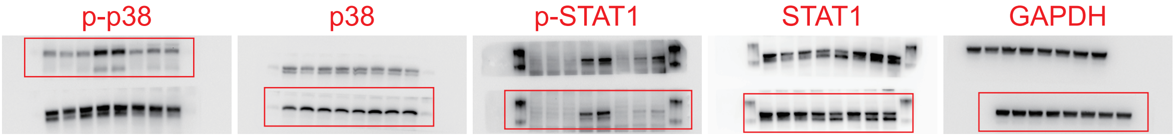

Figure EV4C right panel (THP-1)

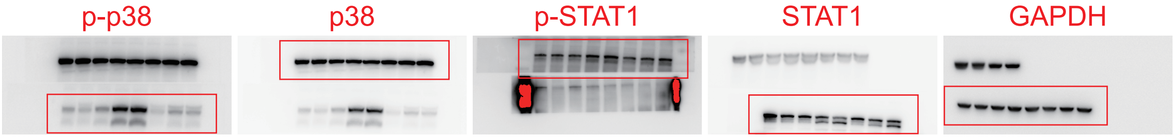

Figure EV4F

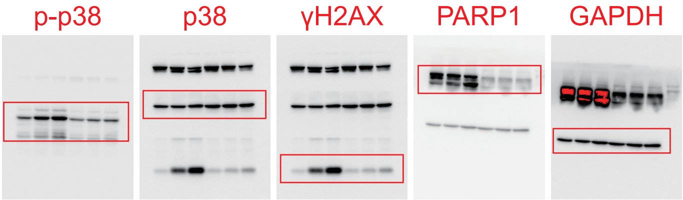

Figure EV4G

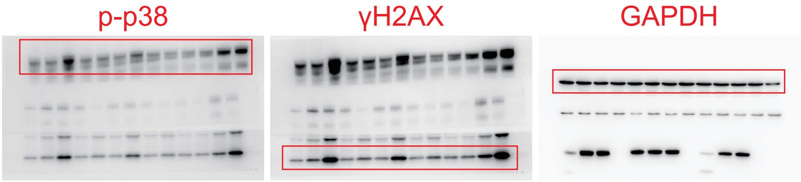

Figure EV4I

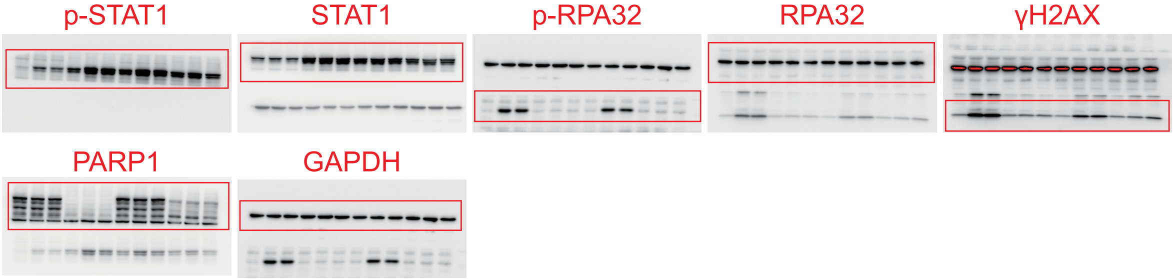

Supplement: Supplementary file 4 — Source Data for Expanded View [file EMMM-15-e16235-s008.zip › Source Data-Figure EV4.pdf]
