# Supplementary figures and images for: Thioparib inhibits homologous recombination repair, activates the type I IFN response, and overcomes olaparib resistance
Source: EMBO Mol Med. 2023 Jan 18;15(3):e16235. doi: 10.15252/emmm.202216235 (PMC9994488; doi:10.15252/emmm.202216235)

Figure EV3A left panel

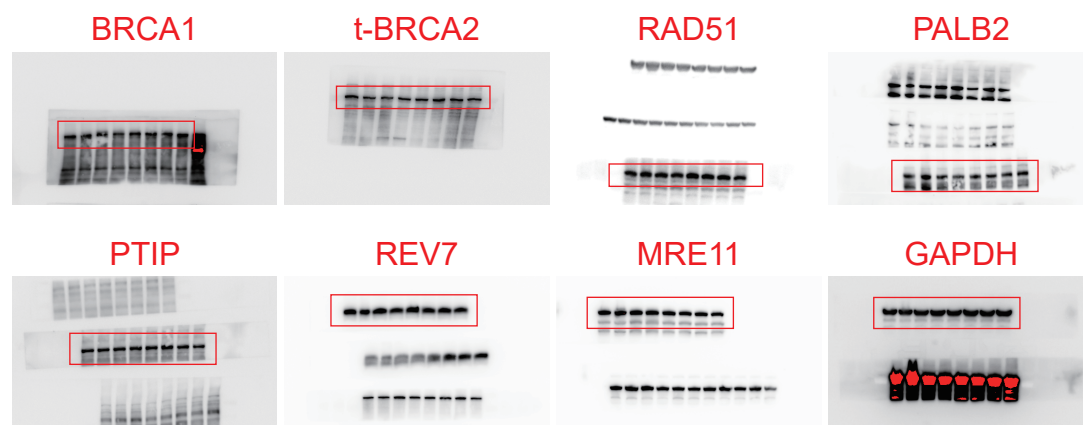

Figure EV3A right panel

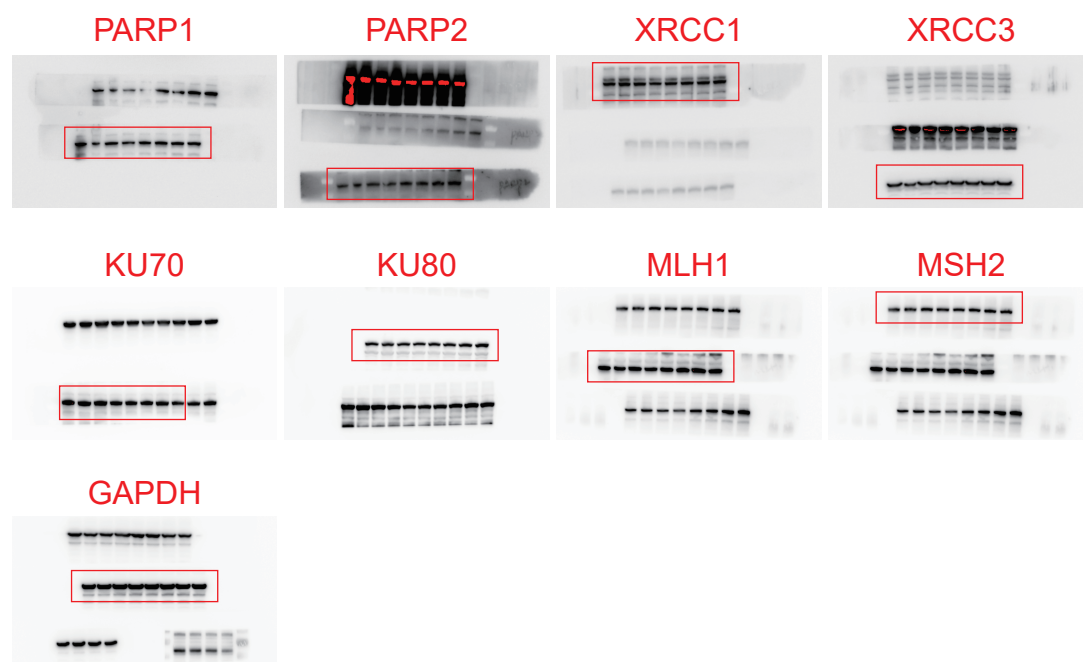

Figure EV3B

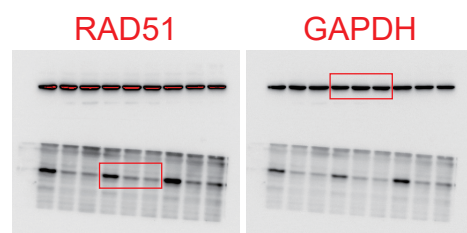

Supplement: Supplementary file 4 — Source Data for Expanded View [file EMMM-15-e16235-s008.zip › Source Data-Figure EV3.pdf]

Figure EV5A

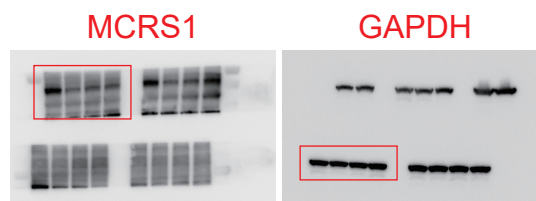

Supplement: Supplementary file 4 — Source Data for Expanded View [file EMMM-15-e16235-s008.zip › Source Data-Figure EV5.pdf]

Figure 5F left panel (JeKo-1)

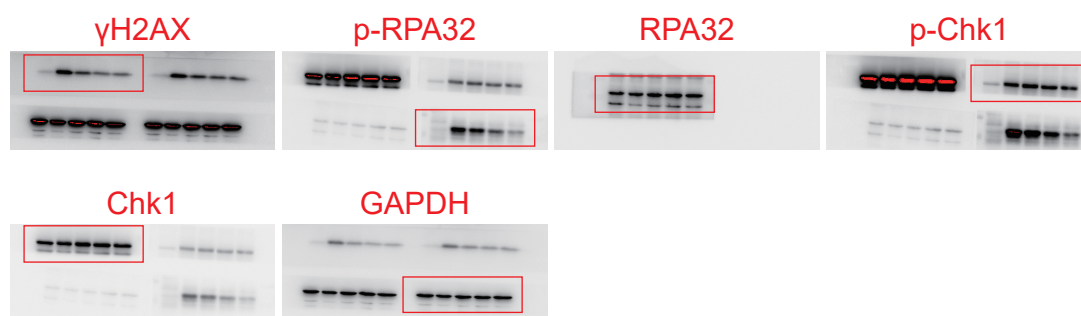

Figure 5F right panel (Capan-1/TP)

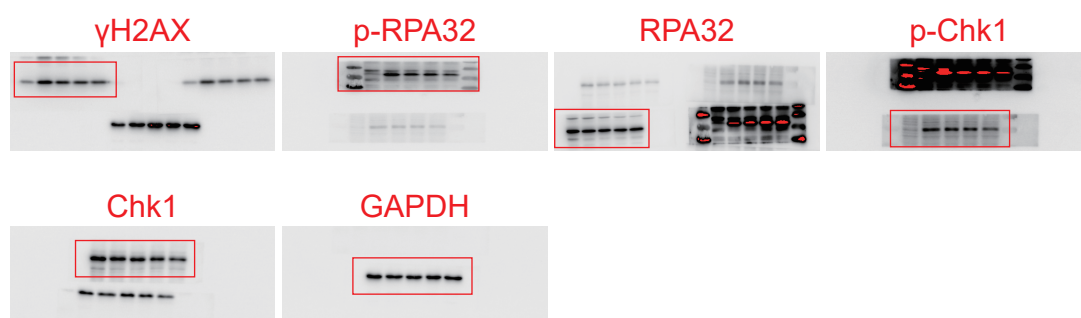

Figure 5H

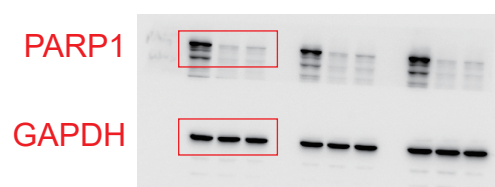

Supplement: Supplementary file 7 — Source Data for Figure 5 [file EMMM-15-e16235-s009.pdf]

Figure 6B left panel (JeKo-1)

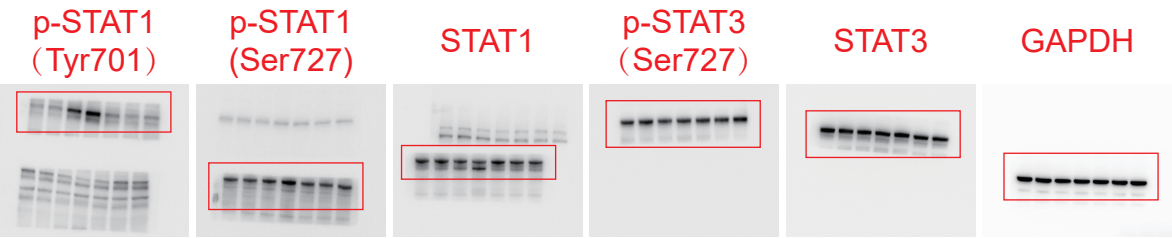

Figure 6B right panel (THP-1)

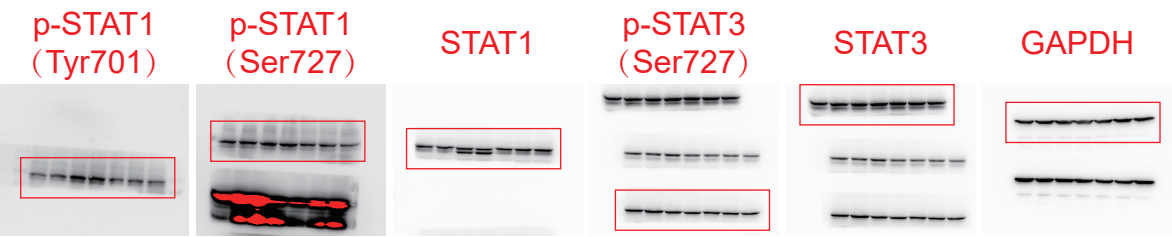

Figure 6D

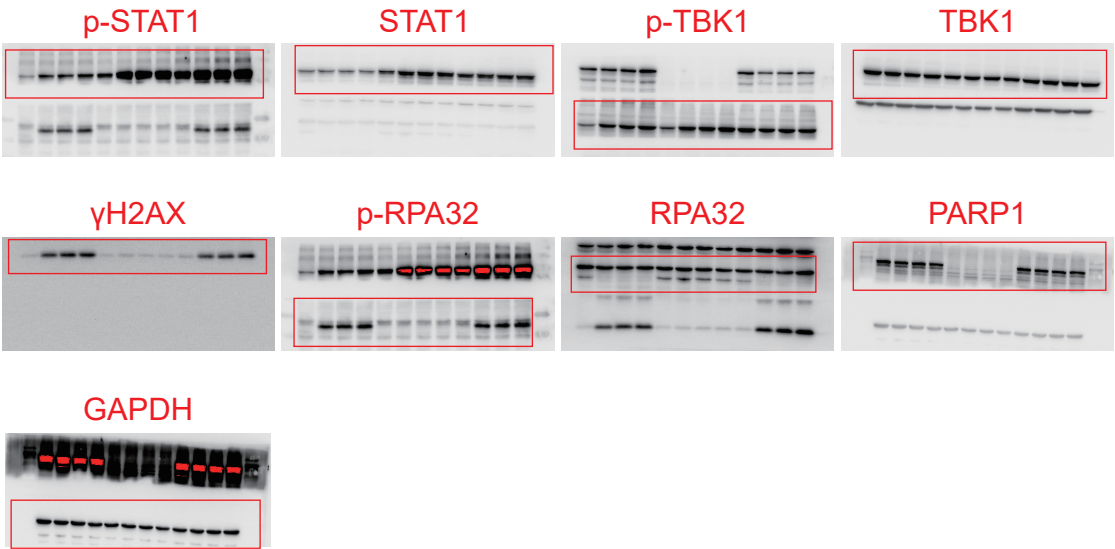

Figure 6F

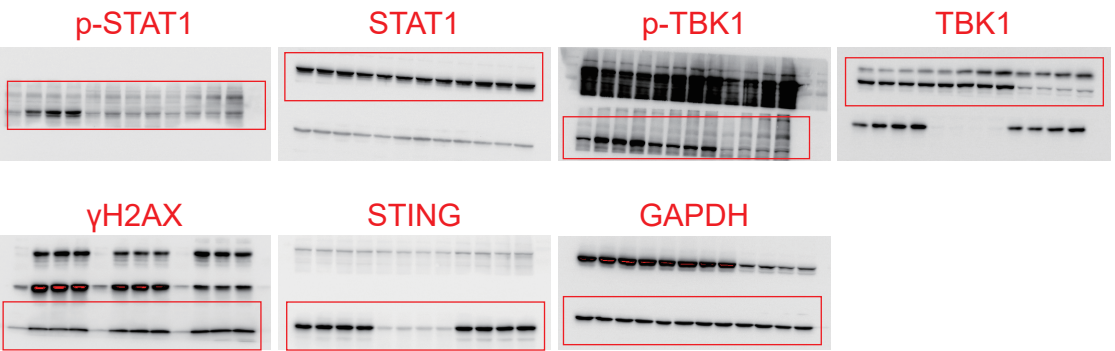

Supplement: Supplementary file 8 — Source Data for Figure 6 [file EMMM-15-e16235-s003.pdf]
