# Supplementary material for: Thioparib inhibits homologous recombination repair, activates the type I IFN response, and overcomes olaparib resistance
Source: EMBO Mol Med. 2023 Jan 18;15(3):e16235. doi: 10.15252/emmm.202216235 (PMC9994488; doi:10.15252/emmm.202216235)

Figure 4B left panel (JeKo-1)

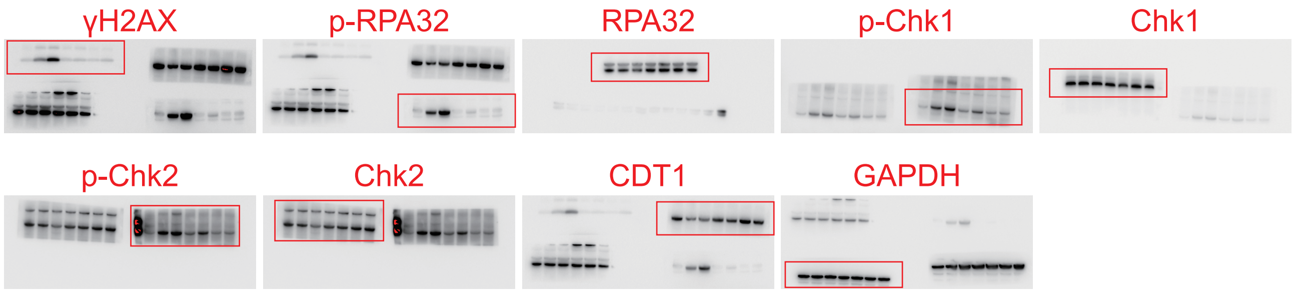

Figure 4B right panel (THP-1)

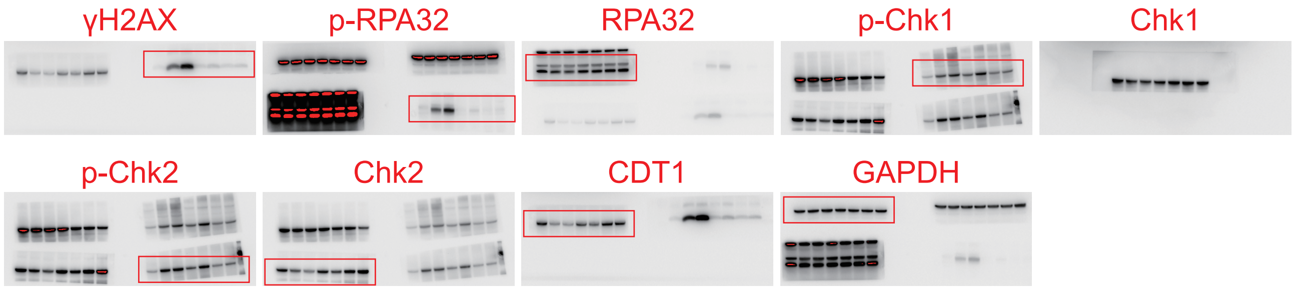

Figure 4E left panel (JeKo-1)

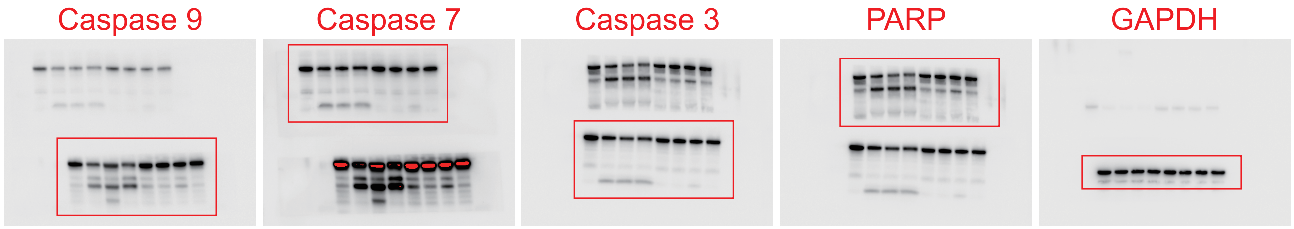

Figure 4E right panel (THP-1)

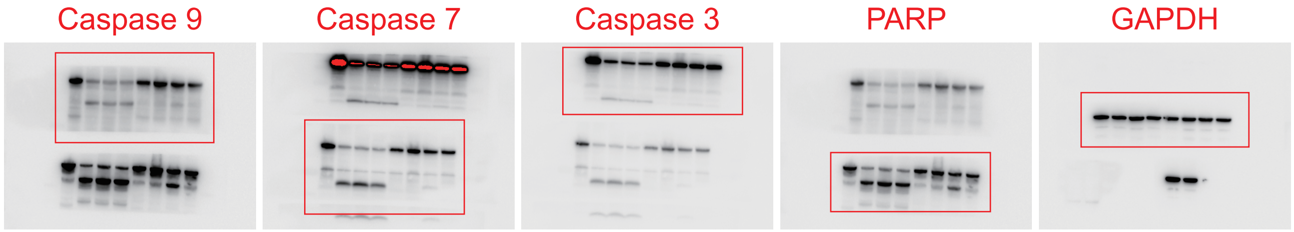

Figure 4F

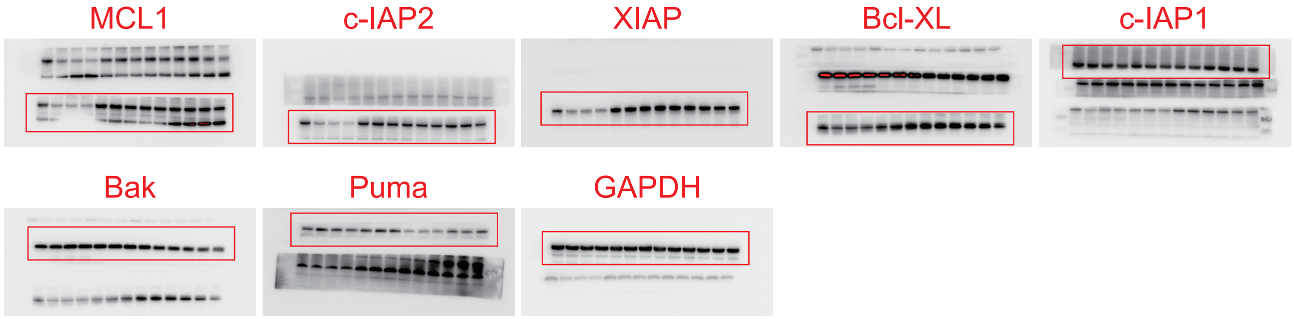

Figure 4G

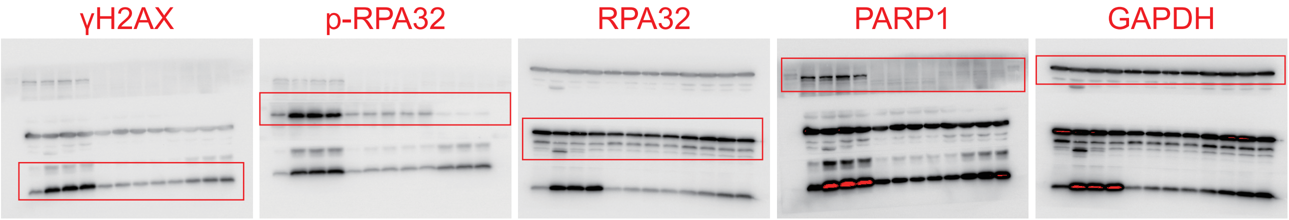

Figure 4H

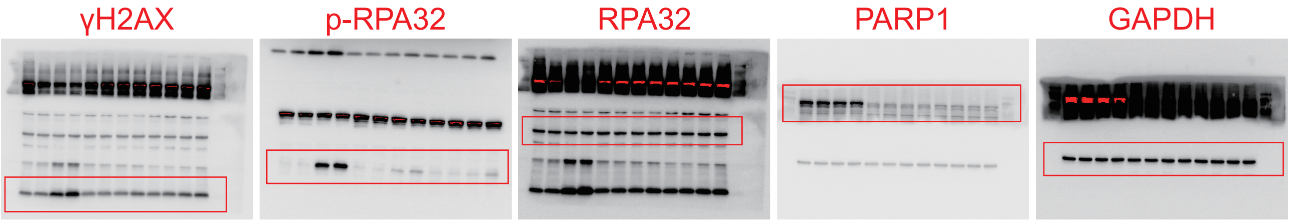

Supplement: Supplementary file 6 — Source Data for Figure 4 [file EMMM-15-e16235-s005.pdf]
