# Supplementary material for: Thioparib inhibits homologous recombination repair, activates the type I IFN response, and overcomes olaparib resistance
Source: EMBO Mol Med. 2023 Jan 18;15(3):e16235. doi: 10.15252/emmm.202216235 (PMC9994488; doi:10.15252/emmm.202216235)
Supplement: Supplementary file 9 [file EMMM-15-e16235-s006.pdf]

EMBO Molecular Medicine

Thioparib Inhibits Homologous Recombination Repair, Activates the Type-I IFN Response, and Overcomes Olaparib Resistance

Limin Wang, Pingyuan Wang, Xiaomin Chen, Hui Yang, Shanshan Song, Zilan Song, Li Jia, Huadong Chen, Xubin Bao, Ne Guo, Xiajuan Huan, Yong Xi, Yanyan Shen, Xinying Yang, Yi Su, Yiming Sun, Yinglei Gao, Yi Chen, Jian Ding, jingyu lang, Ze-Hong Miao, Ao Zhang, and Jinxue He
DOI: 10.15252/emmm.202216235

Corresponding authors: Ze-Hong Miao (zhmiao@simm.ac.cn), Jingyu Lang (jylang@sibs.ac.cn), Ao Zhang (ao6919zhang@sjtu.edu.cn), Jinxue He (jinxue_he@simm.ac.cn)

Review Timeline:

Submission Date:	29th Apr 22
Editorial Decision:	7th Jun 22
Revision Received:	14th Oct 22
Editorial Decision:	21st Nov 22
Revision Received:	12th Dec 22
Editorial Decision:	16th Dec 22
Revision Received:	19th Dec 22
Accepted:	21st Dec 22

Editor: Lise Roth

Transaction Report:

(Note: With the exception of the correction of typographical or spelling errors that could be a source of ambiguity, letters and reports are not edited. Depending on transfer agreements, referee reports obtained elsewhere may or may not be included in this compilation. Referee reports are anonymous unless the Referee chooses to sign their reports.)

7th Jun 2022

Dear Dr. Wang,

Thank you for the submission of your manuscript to EMBO Molecular Medicine, and please accept my apologies for the delay in getting back to you, since one referee needed more time to complete his/her report. We also discussed further the decision with the referees once they had submitted their evaluation. As you will see below, the reviewers raise substantial concerns on your work, which unfortunately preclude its publication in EMBO Molecular Medicine in its current form.

The reviewers find that the question addressed by the study is of potential interest, however they also agree that the mechanistic insight is not sufficient at this stage.
The referees mentioned that a revised version of your manuscript should provide solid evidence on the mechanism of action of thioparib, addressing both genomic instability and immune signaling. On the other hand, they agreed that thioparib lack of specificity was a lesser concern at this stage, and we would thus ask you to address this question in writing in your revised manuscript.

Addressing the reviewers' concerns will be necessary for further considering the manuscript in our journal. As revising the manuscript according to the referees' recommendations appears to require a lot of additional work and experimentation, and given the potential interest of your findings, we are ready to extend the deadline to 6 months with the understanding that acceptance of the manuscript would entail a second round of review.

EMBO Molecular Medicine encourages a single round of revision only and therefore, acceptance or rejection of the manuscript will depend on the completeness of your responses included in the next, final version of the manuscript. For this reason, and to save you from any frustrations in the end, I would strongly advise against returning an incomplete revision. Should you find that the requested revisions are not feasible within the constraints outlined here and prefer, therefore, to submit your paper elsewhere, we would welcome a message to this effect.

If you would like to discuss the revisions further, I remain available via email or zoom.

When submitting your revised manuscript, please carefully review the instructions that follow below. We perform an initial quality control of all revised manuscripts before re-review; failure to include requested items will delay the evaluation of your revision.

We require:

1) A .docx formatted version of the manuscript text (including legends for main figures, EV figures and tables). Please make sure that the changes are highlighted to be clearly visible.

2) Individual production quality figure files as .eps, .tif, .jpg (one file per figure). For guidance, download the 'Figure Guide PDF' (https://www.embopress.org/page/journal/17574684/authorguide#figureformat).

3) A .docx formatted letter INCLUDING the reviewers' reports and your detailed point-by-point responses to their comments. As part of the EMBO Press transparent editorial process, the point-by-point response is part of the Review Process File (RPF), which will be published alongside your paper.

4) A complete author checklist, which you can download from our author guidelines (https://www.embopress.org/page/journal/17574684/authorguide#submissionofrevisions). Please insert information in the checklist that is also reflected in the manuscript. The completed author checklist will also be part of the RPF.

5) It is mandatory to include a 'Data Availability' section after the Materials and Methods. Before submitting your revision, primary datasets produced in this study need to be deposited in an appropriate public database, and the accession numbers and database listed under 'Data Availability'. Please remember to provide a reviewer password if the datasets are not yet public (see https://www.embopress.org/page/journal/17574684/authorguide#dataavailability).
In case you have no data that requires deposition in a public database, please state so in this section.

6) For data quantification: please specify the name of the statistical test used to generate error bars and P values, the number (n) of independent experiments (specify technical or biological replicates) underlying each data point and the test used to calculate p-values in each figure legend. The figure legends should contain a basic description of n, P and the test applied. Graphs must include a description of the bars and the error bars (s.d., s.e.m.). Please indicate exact p values, not a range.

7) We would also encourage you to include the source data for figure panels that show essential data. Numerical data should be provided as individual .xls or .csv files (including a tab describing the data). For blots or microscopy, uncropped images should be submitted (using a zip archive if multiple images need to be supplied for one panel). Additional information on source data

and instruction on how to label the files are available at
.

8) Our journal encourages inclusion of *data citations in the reference list* to directly cite datasets that were re-used and obtained from public databases. Data citations in the article text are distinct from normal bibliographical citations and should directly link to the database records from which the data can be accessed. In the main text, data citations are formatted as follows: "Data ref: Smith et al, 2001" or "Data ref: NCBI Sequence Read Archive PRJNA342805, 2017". In the Reference list, data citations must be labeled with "[DATASET]". A data reference must provide the database name, accession number/identifiers and a resolvable link to the landing page from which the data can be accessed at the end of the reference. Further instructions are available at .

9) We replaced Supplementary Information with Expanded View (EV) Figures and Tables that are collapsible/expandable online. A maximum of 5 EV Figures can be typeset. EV Figures should be cited as 'Figure EV1, Figure EV2" etc... in the text and their respective legends should be included in the main text after the legends of regular figures.
- For the figures that you do NOT wish to display as Expanded View figures, they should be bundled together with their legends in a single PDF file called *Appendix*, which should start with a short Table of Content. Appendix figures should be referred to in the main text as: "Appendix Figure S1, Appendix Figure S2" etc.
- Additional Tables/Datasets should be labeled and referred to as Table EV1, Dataset EV1, etc. Legends have to be provided in a separate tab in case of .xls files. Alternatively, the legend can be supplied as a separate text file (README) and zipped together with the Table/Dataset file.
See detailed instructions here:
.

10) The paper explained: EMBO Molecular Medicine articles are accompanied by a summary of the articles to emphasize the major findings in the paper and their medical implications for the non-specialist reader. Please provide a draft summary of your article highlighting
- the medical issue you are addressing,
- the results obtained and
- their clinical impact.
This may be edited to ensure that readers understand the significance and context of the research. Please refer to any of our published articles for an example.

11) For more information: There is space at the end of each article to list relevant web links for further consultation by our readers. Could you identify some relevant ones and provide such information as well? Some examples are patient associations, relevant databases, OMIM/proteins/genes links, author's websites, etc...

12) Conflict of interest: We updated our journal's competing interests policy in January 2022 and request authors to consider both actual and perceived competing interests. Please review the policy https://www.embopress.org/competing-interests and update your competing interests if necessary.

13) Every published paper now includes a 'Synopsis' to further enhance discoverability. Synopses are displayed on the journal webpage and are freely accessible to all readers. They include a short stand first (maximum of 300 characters, including space) as well as 2-5 one-sentences bullet points that summarizes the paper. Please write the bullet points to summarize the key NEW findings. They should be designed to be complementary to the abstract - i.e. not repeat the same text. We encourage inclusion of key acronyms and quantitative information (maximum of 30 words / bullet point). Please use the passive voice. Please attach these in a separate file or send them by email, we will incorporate them accordingly.
Please also suggest a striking image or visual abstract to illustrate your article as a PNG file 550 px wide x 300-600 px high.

14) As part of the EMBO Publications transparent editorial process initiative (see our Editorial at http://embomolmed.embopress.org/content/2/9/329), EMBO Molecular Medicine will publish online a Review Process File (RPF) to accompany accepted manuscripts.
In the event of acceptance, this file will be published in conjunction with your paper and will include the anonymous referee reports, your point-by-point response and all pertinent correspondence relating to the manuscript. Let us know whether you agree with the publication of the RPF and as here, if you want to remove or not any figures from it prior to publication.
Please note that the Authors checklist will be published at the end of the RPF.

EMBO Molecular Medicine has a "scooping protection" policy, whereby similar findings that are published by others during review or revision are not a criterion for rejection. Should you decide to submit a revised version, I do ask that you get in touch after three months if you have not completed it, to update us on the status.

I look forward to receiving your revised manuscript.

Yours sincerely,

Lise Roth

Lise Roth, PhD
Senior Editor
EMBO Molecular Medicine

***** Reviewer's comments *****

Referee #1 (Comments on Novelty/Model System for Author):

There are several examples where the results are inconsistent among different panels containing the same samples. Many different cell lines were used throughout the manuscript with conflicting results, which makes it hard to understand the underlying mechanisms as they seem to be cell-type dependent. The medical impact is low because thioparib is a non-selective inhibitor acting not only on many different PARP family members but also on unrelated proteins such as p38 and most likely many others. Thus the observed phenotypes are pleiotropic. To show the effect of immune signaling activation on tumor formation, the authors should have used an immunocompetent mouse model.

Referee #1 (Remarks for Author):

In this manuscript Wang et al identified thioparib as a pan-PARP inhibitor with more potent catalytic inhibition compared to olaparib and talazoparib in vitro, in HR-deficient cells and in PARPi-resistant cells deficient in 53BP1 or with BRCA1/2 reversion. Furthermore, they showed that thioparib reduces tumour growth in immunocompromised mice engrafted with BRCA1-deficient breast cancer cells MDA-MB-436 or BRCA2-deficient pancreatic cancer cells Capan-1, as well as olaparib-resistant MDA-MB-436. Thioparib is also more effective in haematological cancer-derived cell lines and mouse xenografts. On the cellular level thioparib inhibits HR, leads to DNA damage, replication stress, S-phase arrest and apoptosis. These effects are driven by PARP1 inhibition. The authors also showed activation of IFN signalling but it remains unclear whether this is driven by PARP7 inhibition or another thioparib target. I have a few major concerns:

1) Thioparib is a non-selective pan-PARP inhibitor, which means that it can induce various adverse effects by impairing different pathways. Moreover, it also inhibits non-PARP targets such as p38. This would be a major concern for cancer therapy as it would result in different side effects on normal tissue, which is especially problematic considering the effect of thioparib on DNA damage, cell cycle arrest, apoptosis and immune signaling. Considering the pleiotropic effects of thioparib, it is challenging to pinpoint direct targets responsible for the observed phenotypes. Therefore I am doubtful of the relevance of these findings for future application of this inhibitor.
2) The results presented in Fig. 6 are inconsistent. The authors cannot claim that thioparib induces IFN signalling through PARP7 inhibition. Details are listed below (starting at point 10).
3) The effects of thioparib on immune signaling should be studied in immunocompetent mice.
4) Various cell lines were used for different experiments. The authors should have consistently used at least two cell lines for all functional experiments as the effects of thioparib seem to be cell type-dependent (see point 10).
Detailed comments (as they appear in the manuscript):
1) Introduction
Please consult the recent perspective on PARPi cytotoxicity vs catalytic inhibition PMID: 35259019.
2) Results
In PMID: 30088474 it was shown that PARPi do not have an effect on DNA dissociation.
3) Talazoparib is missing in Fig. 1b.
4) Figures need to be restructured in such a way that panels are called in the text in a sequential order. For example, Fig. 1f,g should be moved to Fig. 2, Fig. 2c to Fig. 1...
5) line 148 - Fig. 1c (not 2c)
6) line 160 - What do you mean by 'similar allosteric influences'?
7) Why did the authors switch to JeKo-1 and THP-1 cells for DDR analysis when in the first part of the manuscript all the experiments were performed with MDA-MB-436?
8) Fig. 4b: pChk1 and pChk2 are also induced after talazoparib treatment. CDT1 did not 'mostly disappear' but was reduced after thioparib.
9) line 387: 'following thioparib treatment'

10) Fig. 6b,d: There is a discrepancy between the results presented in panels b and d. In panel b thioparib strongly induces pSTAT1, whereas in panel d the effect is negligible. This could be due to the fact that different cell lines were used (Jeko-1 and THP-1 in panel b, HT-29 in panel d).
11) Another discrepancy in Fig. 6d and 6f. In Fig. 6d STAT1 levels are increased in PARP1 KO even without thioparib treatment. In Fig. 6f STAT1 levels are not increased in PARP1 KO. Please clarify this.
12) Fig. 6e: Please also show absolute values for IFNb1 and CXCL10 expression levels without thioparib treatment such that it's possible to compare the effect of PARP1/7 KO in untreated conditions. This is related to point 11).
13) PARP7 blot is missing in Fig. 6d to show that PARP7 was knocked out.
14) PARP7 was shown to inhibit TBK1 phosphorylation (PMID: 27089381). Please show pTBK1 on the blots in Fig. 6d,f.
15) pSTING and pTBK1 were only increased in one cell line (THP-1). However, they were also increased after talazoparib or olaparib treatment (Fig. S4g), suggesting again that thioparib effects are not due to PARP7 inhibition. PARP1/2 inhibitors were previously shown to induce cGAS/STING in NSCLC and TNBC (PMID: 31015319, PMID: 30777870). The fact that pSTAT1 is induced in PARP1 KO cells after thioparib treatment suggests that thioparib effects on pSTAT1 are neither PARP1 nor PARP7 dependent.
16) Fig. 6f: Why does thioparib have stronger effect on pSTAT1 in PARP1 KO cells?
17) Experiments in Fig. 6f should be performed in STING/TBK1 single KO (not combined with PARP1 KO) to determine whether pSTAT1 induced by thioparib is STING/TBK1-dependent.
18) What is the combined effect of olaparib/talazoparib and thioparib on IFN signalling?
19) Fig. 6g: How does thioparib inhibit p38 phosphorylation?

Referee #2 (Remarks for Author):

The authors provide evidence that thioparib is a new generation of PARP inhibitors that sensitizes tumors defective in HR and triggers an IFN type 1 response. Overall the work is well structured and the conclusions are supported from the original observations. What remains unclear is the mechanism through which thioparib inhibits HR. Some conflicting results (increased Rad51 levels and inhibition of HR in way that differ from that of other known inhibitors makes it somewhat difficult to understand any putative mechanism. Also, the authors could better explain what is the functional consequence of type IFN signaling on tumor survival in this context or how specific the IFN response is to thioparib.
Further comments are shown below:
358: What "nonfunctional Rad51 foci" really mean? If possible, the authors could test whether knockdown of Rad51 sensitizes cells to thioparib.
Line 362-368: It remains unclear how thioparib inhibits HR if it impairs this DNA repair pathways in a way that differs from that of other PARP1 inhibitors.
Line 363: The sentence does not read well. Is it e.g., "to lead" or "leading to" HR inhibition?
Line 355: Please fix the square typo
Line 416-417: There are recent reports indicating that DNA damage (as well as R-loops) triggers the accumulation of ds and/or ssDNAs in the cytoplasm of cells. Is thioparib triggering a similar response? The authors could stain for DAPI-associated chromatin fragments or use antibodies against ssDNA fragments or even R-loops in these cells. It is also somewhat unclear how the thioparib-induced type I IFN response is linked to the remaining story on PARP inhibition.
The specificity of the IFN response to thioparib (compared to other inhibitors) is not shown.

Referee #3 (Comments on Novelty/Model System for Author):

Caveats regarding the adequacy of the cell lines used in these studies are noted in my specific comments.

Referee #3 (Remarks for Author):

This interesting paper reports the discovery and characterization of a new inhibitor of the poly-ADP ribose polymerase (PARP) enzymes, thioparib, and describes the compound's in vitro and in vivo activity in several settings. The key novel features of thioparib that distinguish it from existing drugs include its high potency, its activity against cancer cells refractory to other PARP inhibitors (PARPi) via several mechanisms, and its ability to heighten the anti-tumour innate immune response via the cGAS/STING pathway.

The experimental evidence provided in this well-written paper supports many of the conclusions drawn by the authors, but there are a few caveats listed in my specific comments below that need to be addressed. The discovery and characterization of thioparib represents an original advance in the field that is likely to be of translational and clinical impact, and of interest to the readership of EMBO Molecular Medicine.

Specific comments are as follows.

(1) Introduction (lines 102-111): The authors refer to their previous work (Chen et al 2019) to suggest that only a weak correlation exists between PARP1-DNA trapping and the cytotoxicity of PARPi such as talazoparib, olaparib and rucaparib. Instead, they suggest that differences in the cytotoxicity of PARPi originate from their abilities to inhibit the autoPARylation of PARP1 - but such inhibition in turn, prevents its dissociation from DNA.

Thus, isn't inhibited autoPARylation expected to trap PARP on DNA? If so, why the weak correlation of trapping with cytotoxicity? Please explain. Similar questions apply to the statements in the Results section (lines 126-132).

The authors make a somewhat discrepant statement elsewhere (lines 285-87), where they suggest that PARPi kill BRCA-mutant cells via inhibition of BER. How does this relate to cytotoxicity induced by DNA trapping of PARP?

(2) Figure 2c/2d: The authors claim that thioparib is effective in killing BRCA1/2-mutant cancer cells that have acquired resistance via somatic reversion mutations. This is potentially an important claim - but it is difficult to reconcile with the published evidence that somatic reversion mutants in BRCA1/2 at least partially restore DNA repair by HR. Does HR restoration occur in the revertant cell lines used in this study? If so, by what mechanism does thioparib kill the revertant cells? These results seem preliminary, and need further experimental analysis. Similar studies to those carried out in Fig 4 would provide some insight.

On this point, the authors later show (Fig 4g, h and Table S6) that PARP1 depletion decreases the sensitivity of revertant cells to thioparib. This finding is puzzling if the revertants have restored HR capacity, and suggest that thioparib may have unrelated mechanisms of action.

(3) Figure 2e: Similar concerns apply to the authors' work on the primary PARP-resistant PDX model BR-00-28, generated from a BRCA1-deficient human breast cancer. Do these cells retain HR capacity to any degree? If so, by what mechanism does thioparib work here? Again, the results shown seem preliminary and need further study.

Point-by-point Response

Response to the Review

Referee #1 (Remarks for Author):

In this manuscript Wang et al identified thioparib as a pan-PARP inhibitor with more potent catalytic inhibition compared to olaparib and talazoparib in vitro, in HR-deficient cells and in PARPi-resistant cells deficient in 53BP1 or with BRCA1/2 reversion. Furthermore, they showed that thioparib reduces tumour growth in immunocompromised mice engrafted with BRCA1-deficient breast cancer cells MDA-MB-436 or BRCA2-deficient pancreatic cancer cells Capan-1, as well as olaparib-resistant MDA-MB-436. Thioparib is also more effective in haematological cancer-derived cell lines and mouse xenografts. On the cellular level thioparib inhibits HR, leads to DNA damage, replication stress, S-phase arrest and apoptosis. These effects are driven by PARP1 inhibition. The authors also showed activation of IFN signalling but it remains unclear whether this is driven by PARP7 inhibition or another thioparib target. I have a few major concerns:

Q1.1. Thioparib is a non-selective pan-PARP inhibitor, which means that it can induce various adverse effects by impairing different pathways. Moreover, it also inhibits non-PARP targets such as p38. This would be a major concern for cancer therapy as it would result in different side effects on normal tissue, which is especially problematic considering the effect of thioparib on DNA damage, cell cycle arrest, apoptosis and immune signaling. Considering the pleiotropic effects of thioparib, it is challenging to pinpoint direct targets responsible for the observed phenotypes. Therefore I am doubtful of the relevance of these findings for future application of this inhibitor.

Response: We thank the reviewer for the insightful comments and suggestions. As the reviewer notes, our data demonstrate that thioparib is a pan-PARP inhibitor. However, we do not agree with the reviewer on the point that "it also inhibits non-PARP targets such as p38". The data, now included in **Appendix Fig. S2K**, show that thioparib is unable to inhibit the *in vitro* activity of p38α, p38β, p38σ, or p38γ, indicating that p38 MAPK isoforms are likely not the direct targets of thioparib.

We agree that it is important to pinpoint targets responsible for the observed phenotypes, and now we provide additional evidence for the identification of a bona fide mechanism of thioparib. These data are now presented in the revised **Fig. 5, Fig.6,** and **Fig.EV4**.

Q1.2. The results presented in Fig. 6 are inconsistent. The authors cannot claim that thioparib induces IFN signalling through PARP7 inhibition. Details are listed below (starting at point 10).

10) Fig. 6b,d: There is a discrepancy between the results presented in panels b and d. In panel b thioparib strongly induces pSTAT1, whereas in panel d the effect is negligible. This could be due to the fact that different cell lines were used (Jeko-1 and THP-1 in panel b, HT-29 in panel d).

Response: We apologize for any confusion in the immunoblots. Actually, thioparib also strongly induces pSTAT1 in HT-29 cells. We now have replaced an improved blot from longer exposure times (**new Fig.6D**).

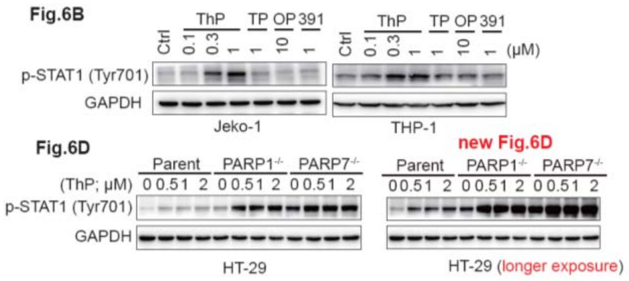

Fig. R1. Concentration-dependent increase in p-STAT1 in JeKo-1, THP-1 (upper; Fig 6B), and HT-29 cells (lower; updated Fig. 6D) exposed to thioparib.

Q1.3. The effects of thioparib on immune signaling should be studied in immunocompetent mice.

Response: As suggested by the reviewer, we determined the effects of thioparib in immunocompetent MC38-bearing mice. The new results confirm the immune activities of thioparib as shown in the *in vitro* human cell models (new **Fig.6G-I**).

Q1.4. Various cell lines were used for different experiments. The authors should have consistently used at least two cell lines for all functional experiments as the effects of thioparib seem to be cell type-dependent (see point 10).

Response: Thanks for the reviewer's suggestion. We apologize if the rationale for selecting various cell lines was not made clear in the manuscript. We used JeKo-1(BRCA1/2 mutant), THP-1(RAD51D mutant), and HT-29(POLQ mutant) cells for most of the functional experiments as these cells are extremely sensitive to thioparib but not to other PARPi, and we used U2OS cells for the DR-GFP assay because these cells are readily transfectable.

As our response to the reviewers' comments presented above, the effects of thioparib are consistent in the tested cell lines (**Fig 4B&4G**, **new Fig 6B&6D**).

Q1.5. Detailed comments (as they appear in the manuscript):

1) Introduction

Please consult the recent perspective on PARPi cytotoxicity vs catalytic inhibition PMID: 35259019.

Response: Thanks for the suggestion. We now quote and discuss this very relevant paper.

Q1.6. 2) Results

In PMID: 30088474 it was shown that PARPi do not have an effect on DNA dissociation.

Response: In PMID: 30088474, the authors utilized FA assays to explore the association and dissociation kinetics of PARP1-DNA complexes in the absence of NAD^+; PARP1 dissociation was initiated by additional excessive DNA oligomer; and the data showed no effect of PARPi on the release of DNA.

In this current study, we used a biochemical system similar to that reported previously (PMID: 26217019; 30675909), and the association reaction was measured by **mixing DNA oligomer, PARP1 enzyme, and** $\mathbf{NAD^+}$. The inclusion of NAD^+ results in auto-PARylation of PARP1 itself, which led to the rapid dissociation of PARP1 from the DNA within 10 min. Under this condition, PARPi significantly decreases the rate of PARP1 dissociation from DNA.

In PMID: 26217019, the authors performed PARP1-DNA binding assay both in the presence and absence of NAD^+, and pointed out that PARPi were capable of enhancing PARP-DNA binding in the presence of NAD^+, which is consistent with the results from our previous reports and this study (PMID:30675909).

Based on the previous studies, we proposed that PARP-DNA binding activity is an important factor for PARPi-induced cytotoxicity. Our results are in agreement with our observations in biochemical systems.

Q1.7. 3) Talazoparib is missing in Fig. 1b.

Response: Talazoparib is included and presented in new Fig.1B.

Q1.8. Figures need to be restructured in such a way that panels are called in the text in a sequential order. For example, Fig. 1f,g should be moved to Fig. 2, Fig. 2c to Fig. 1....

Response: We think that the figures show a clear logic in our manuscript. In Fig.1, our data indicate that thioparib induces synthetic lethality in BRCA-deficient tumors (PARPi-sensitive models) both *in vitro* and *in vivo* (Table S3 and Fig.1F, G). Its potency is 50- to 4200-fold stronger than that of other PARPi, including the strongest PARPi talazoparib. According to the above findings, we then explored the antitumor effect of thioparib in PARPi-resistant models *in vitro* and *in vivo* (Fig.2). We have

carefully re-checked the text and made sure that the panels were called in a sequential order.

Q1.9. 5) line 148 - Fig. 1c (not 2c).

Response: We have corrected the error.

Q1.10. 6) line 160 - What do you mean by 'similar allosteric influences'?

Response: Thanks. The statement has been revised.

Q1.11. 7) Why did the authors switch to JeKo-1 and THP-1 cells for DDR analysis when in the first part of the manuscript all the experiments were performed with MDA-MB-436?

Response: MDA-MB-436 is highly sensitive to most of the PARPi, and we found that thioparib (ThP) promoted DNA damage to extents that were comparable to those induced by talazoparib (TP) (**Appendix Fig.S1B**). To clarify the difference between thioparib and other PARPi, we thus switch to JeKo-1 and THP-1 cells for DDR analysis. The data showed that DDR alterations were rapidly induced within 6-12 hours by thioparib in both cells. Compared with other PARPi, thioparib treatment has caused significantly greater DNA damage in both cell lines (**Fig.4B**). In the first part of the manuscript, we performed our experiments not only in MDA-MB-436 cells, but also in Capan-1, JeKo-1, THP-1, etc.

Q1.12. 8) Fig. 4b: pChk1 and pChk2 are also induced after talazoparib treatment. CDT1 did not 'mostly disappear' but was reduced after thioparib.

Response: The statement has been revised as suggested.

Q1.13. 9) line 387: 'following thioparib treatment'

Response: We have corrected the error.

Q1.14. 10) Fig. 6b,d: There is a discrepancy between the results presented in panels b and d. In panel b thioparib strongly induces pSTAT1, whereas in panel d the effect is negligible. This could be due to the fact that different cell lines were used (Jeko-1 and THP-1 in panel b, HT-29 in panel d).

Response: As described in our response to the reviewer's comment #2, we provide improved blots from longer exposure times (**new Fig.6D**).

Q1.15. 11) Another discrepancy in Fig. 6d and 6f. In Fig. 6d STAT1 levels are increased in PARP1 KO even without thioparib treatment. In Fig. 6f STAT1 levels are not increased in PARP1 KO. Please clarify this.

Response: We thank the reviewer for pointing this out. We utilized CRISPR-Cas9 and two distinct guide RNAs to generate PARP1 knockout HT-29 clones (#KO1 and KO2; **Fig. 4G**). As suggested by the reviewer, the data in Fig. 6D and 6F were re-checked carefully, and found that there is some difference between the two KO clones.

Indeed, the total levels of STAT1 are increased in PARP1#KO1 clone (**new Fig.6D**), but not in #KO2 clone (previous Fig.6F, **now the data was removed**). However, regardless of the levels of STAT1, treatment of both #KO1 and KO2 clones with thioparib led to a significant increase in p-STAT1.

Q1.16. 12) Fig. 6e: Please also show absolute values for IFNb1 and CXCL10 expression levels without thioparib treatment such that it's possible to compare the effect of PARP1/7 KO in untreated conditions. This is related to point 11).

Response: We thank the reviewer for the suggestion. We have included the absolute mRNA levels of *IFNB1* and *CXCL10* without treatment in the parent, PARP1, and PARP7 KO cells (**new Figure. EV 4A**).

Q1.17. 13) PARP7 blot is missing in Fig. 6d to show that PARP7 was knocked out.

Response: Based on the reviewer's suggestions we analyzed the expression levels of PARP7 in parent and PARP7 KO cells by western blotting. We find that, in line with previous studies, commercially available anti-PARP7 antibody (ab84664) failed to detect PARP7 protein (PMID: 33799807). As seen below, several bands at approximately 70-100 kDa were detected in both parent and PARP7 KO cells.

Since it is difficult to identify a reliable commercially available antibody to detect PARP7 protein, we sequenced the target genome of PARP7 KO monoclonal cells. As seen below (**Appendix Fig. S2D**), PARP7 KO cells contained an insertion mutation (903_904insC) and a deletion mutation (903_923del) causing a frameshift at the targeted locus. PARP7 mRNA levels were confirmed by RT-qPCR analysis.

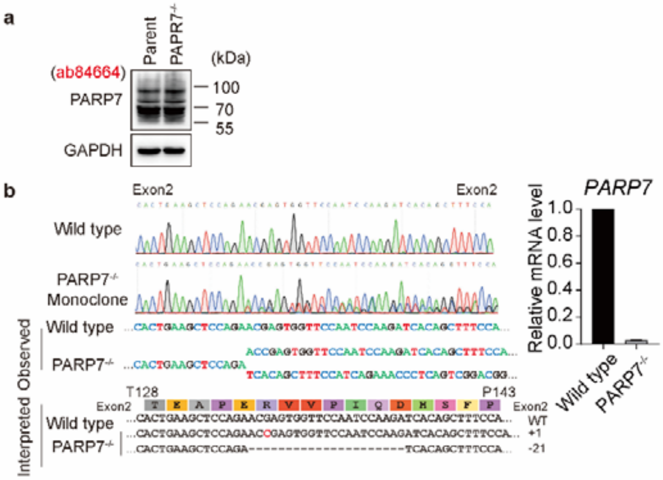

Fig. R2. Confirmation of HT-29 PARP7 knockout cells. (**a**) Blotting with anti-PARP7 (ab84664) resulted in nonspecific bands across approximately 70-100 kDa. (**b**) Sequencing and RT-qPCR analysis of PARP7 KO cells, showing insertions/deletions resulting in frameshift mutations.

Q1.18. 14) PARP7 was shown to inhibit TBK1 phosphorylation (PMID: 27089381). Please show pTBK1 on the blots in Fig. 6d,f.

Response: Following the suggestions, we added pTBK1 in **new Fig.6D, 6F.**

Q1.19. 15) pSTING and pTBK1 were only increased in one cell line (THP-1). However, they were also increased after talazoparib or olaparib treatment (Fig. S4g), suggesting again that thioparib effects are not due to PARP7 inhibition. PARP1/2 inhibitors were previously shown to induce cGAS/STING in NSCLC and TNBC (PMID: 31015319, PMID: 30777870). The fact that pSTAT1 is induced in PARP1 KO cells after thioparib treatment suggests that thioparib effects on pSTAT1 are neither PARP1 nor PARP7 dependent.

Response: These comments and suggestions are insightful, which directed our exploration toward the identification of the mechanism of how thioparib affects pSTAT1 and IFN signaling. As a result, we have generated a substantial amount of new data, including the following:

1) Type I IFN response to thioparib or RBN-2397 was diminished in STING or TBK1 KO cells, demonstrated by abrogation of pSTAT1; while a highly selective PARP1 inhibitor AZD5305 had little effect on pSTAT1 (**new Fig.6F; Appendix Fig. S2H**).
2) As shown in **Fig.6D &6E**, only PARP7 KO is able to prevent the additional increase in p-STAT1 induced by thioparib.
3) Compared to single KO, the double KO of PARP1/7 completely diminished thioparib-induced type I IFN response (**Fig. EV 4I and 4J**).
4) *In vivo* data suggest that PARP7, but not PARP1, is indispensable in antitumor immunity of thioparib in an immunocompetent MC38 mouse model (**new Fig. 6G-I**).

Taken together, these results support that PARP7, but not PARP1, makes a major contribution to the immunomodulatory activity of thioparib in the tested cell lines.

Q1.20. 16) Fig. 6f: Why does thioparib have stronger effect on pSTAT1 in PARP1 KO cells?

Response: The gray analysis of the pSTAT1 bands were shown below (R3; previous Fig. 6F). In fact, the data showed that thioparib treatment caused comparable effects (NOT stronger) in increasing p-STAT1 in parental and PARP1 KO cells.

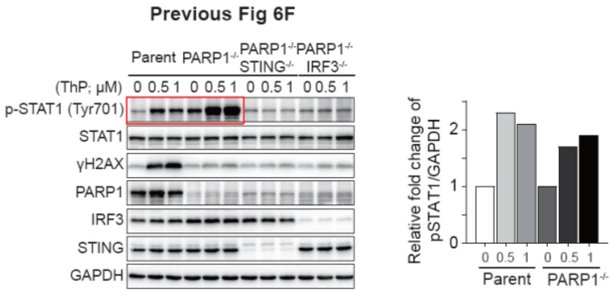

Fig. R3. Western blot analysis of p-STAT1 in HT-29 parent and KO cells.

The reviewer suggested that Fig. 6F should be performed in STING/TBK1 single KO (comment #17), we therefore removed these data from our revised manuscript.

Q1.21. 17) Experiments in Fig. 6f should be performed in STING/TBK1 single KO (not combined with PARP1 KO) to determine whether pSTAT1 induced by thioparib is STING/TBK1-dependent.

Response: We agree with the reviewer and we have repeated the western blot in STING/TBK1 single KO cell lines (**new Fig.6F**).

Q1.22. 18) What is the combined effect of olaparib/talazoparib and thioparib on IFN signalling?

Response: As suggested by the reviewer, we evaluated the combined effect of olaparib and thioparib on IFN signaling. The data revealed that the combination show antagonistic effects on IFN-inducible genes, while olaparib alone had no significant impact.

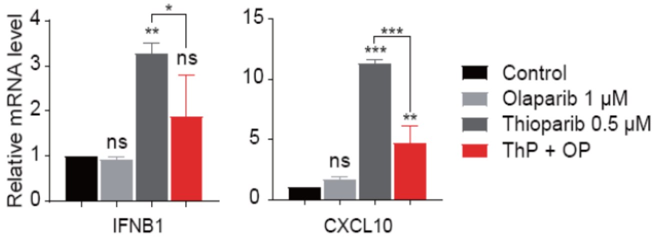

Fig. R4. The combined effect of olaparib and thioparib on IFN signaling in HT-29 cells.

Q1.23. 19) Fig. 6g: How does thioparib inhibit p38 phosphorylation?

Response: As described in our response to the reviewer's comment #1, p38 MAPK isoforms are not direct targets of thioparib (**Appendix Fig. S2K**). Interestingly, we found that p38 activation induced by thioparib was abolished by PARP1 depletion (**Fig. EV4F**). We also observed that p-p38 was associated with high levels of γH2AX

(**Fig. EV4G**). Similar to these results, Wood et al. (PMID: 19564926) showed that p38 MAPK was activated by stimuli that induce DNA DSBs, resulting in its nuclear translocation. Together, these data suggest that thioparib induced p38 phosphorylation most likely through the induction of DNA damage.

Referee #2 (Remarks for Author):

The authors provide evidence that thioparib is a new generation of PARP inhibitors that sensitizes tumors defective in HR and triggers an IFN type 1 response. Overall the work is well structured and the conclusions are supported from the original observations. What remains unclear is the mechanism through which thioparib inhibits HR. Some conflicting results (increased Rad51 levels and inhibition of HR in way that differ from that of other known inhibitors makes it somewhat difficult to understand any putative mechanism. Also, the authors could better explain what is the functional consequence of type IFN signaling on tumor survival in this context or how specific the IFN response is to thioparib. Further comments are shown below:

Q2.1. 358: What "nonfunctional Rad51 foci" really mean? If possible, the authors could test whether knockdown of Rad51 sensitizes cells to thioparib.

Response: The statement has been revised. Indeed, knockdown of Rad51 sensitizes cells to all the tested PARP inhibitors (thioparib, olaparib, and talazoparib). The data are now presented in **Fig. EV3B**.

Q2.2 Line 362-368: It remains unclear how thioparib inhibits HR if it impairs this DNA repair pathways in a way that differs from that of other PARP1 inhibitors.

Response: We thank the reviewer for the constructive suggestions. To address this issue, we generated stable U2OS PARP1$^{-/-}$ cells to determine the contribution of PARP1 in HR suppression. As shown in **new Fig. 5H**, KO of PARP1 completely (~100%) rescued the suppressive effect of PARPi (including talazoparib, olaparib, and Cpd 391) on HR repair. However, PARP1 depletion only partially (~50%) rescued the repression of HR function caused by thioparib. PARP1 is necessary but only partially contributes to HR suppression in thioparib-treated cells.

Therefore, we assume that PARP1 inhibition is one of the mechanisms that impair HR upon thioparib treatment. However, thioparib also impairs HR repair in a way that differs from that of other PARPi, and the unknown factors likely make up the other 50% effect. We hope to address this specific issue in the future.

Q2.3. Line 363: The sentence does not read well. Is it e.g., "to lead" or "leading to" HR inhibition?.

Response: This has been revised.

Q2.4. Line 355: Please fix the square typo.

Response: The typo has been corrected.

Q2.5. Line 416-417: There are recent reports indicating that DNA damage (as well as R-loops) triggers the accumulation of ds and/or ssDNAs in the cytoplasm of cells. Is thioparib triggering a similar response? The authors could stain for DAPI-associated chromatin fragments or use antibodies against ssDNA fragments or even R-loops in these cells.

Response: As suggested by the reviewer, we stained cytosolic dsDNA with PicoGreen in thioparib-treated cells. We found that thioparib treatment was able to induce the formation of cytosolic dsDNA significantly, which can be diminished to the level in untreated cells after KO of PARP1, but not PARP7 (**Fig. EV4H**).

Q2.6. It is also somewhat unclear how the thioparib-induced type I IFN response is linked to the remaining story on PARP inhibition.

Response: As noted above and in response to reviewer 1, we now provide more evidence for the mechanisms of how thioparib triggers type I IFN response using a gene knockout system and *in vivo* immunocompetent mice models (**Fig.6, Fig.EV4**). The results further support our findings that PARP7-STING/TBK1 pathway plays crucial roles in thioparib-induced IFN response. We also showed that PARP1-dependent p38 MAPK signaling increased type I IFN response parallelly in the thioparib-treated cells. Our *in vivo* data suggest that PARP7 is indispensable in the antitumor immunity of thioparib.

Q2.7. The specificity of the IFN response to thioparib (compared to other inhibitors) is not shown.

Response: To clarify the specificity of thioparib on the IFN response, we used selective PARP1 inhibitor AZD5305, other PARPi (including olaparib, talazoparib, and Cpd 391), and PARP7 inhibitor RBN-2397 as a control. The data show that the type I IFN response is strongly induced by thioparib or RBN-2397, but not AZD5303 or other PARPi (**Fig. 6A-C; Appendix Fig. S2E, F, G, H**).

Referee #3 (Remarks for Author):

This interesting paper reports the discovery and characterization of a new inhibitor of the poly-ADP ribose polymerase (PARP) enzymes, thioparib, and describes the compound's in vitro and in vivo activity in several settings. The key novel features of thioparib that distinguish it from existing drugs include its high potency, its activity against cancer cells refractory to other PARP inhibitors (PARPi) via several mechanisms, and its ability to heighten the anti-tumour innate immune response via the cGAS/STING pathway.

The experimental evidence provided in this well-written paper supports many of the conclusions drawn by the authors, but there are a few caveats listed in my specific

comments below that need to be addressed. The discovery and characterization of thioparib represents an original advance in the field that is likely to be of translational and clinical impact, and of interest to the readership of EMBO Molecular Medicine. Specific comments are as follows.

Q3.1. (1) Introduction (lines 102-111): The authors refer to their previous work (Chen et al 2019) to suggest that only a weak correlation exists between PARP1-DNA trapping and the cytotoxicity of PARPi such as talazoparib, olaparib and rucaparib. Instead, they suggest that differences in the cytotoxicity of PARPi originate from their abilities to inhibit the autoPARylation of PARP1 - but such inhibition in turn, prevents its dissociation from DNA.

Thus, isn't inhibited autoPARylation expected to trap PARP on DNA? If so, why the weak correlation of trapping with cytotoxicity? Please explain. Similar questions apply to the statements in the Results section (lines 126-132).

Response: We appreciate the reviewer's comments. Indeed, PARPi and thioparib could potentially "trap" PARP1 on DNA by preventing autoPARlyation. In our previous work, we have utilized various strategies to prove that there was a weak correlation between PARP1-DNA trapping and the cytotoxicity of PARPi (PMID: 30675909)

In the "PARP1 trapping" model introduced by Dr. Thomas Helleday (Mol Oncol 2011;5:387-393; Biomolecules 2012;2: 635-49), trapping is defined as the chromatin binding PARP1; Subsequently, the Pommier's group introduced MMS to treat cells in order to detect the chromatin binding PARP1, or called PARP1-DNA complexes. We previously carefully analyzed the possible reasons for "the weak correlation of trapping with cytotoxicity" in the section of discussion (PMID: 30675909). Briefly, the reasons include the following:

1) It is difficult to accurately detect PARP1 that only binds to DNA (PARP1-DNA complexes in the chromatin), because PARP1 has been shown to interact with RNA and many nuclear proteins such as histones, XRCC6, and PCNA. Thus, the detected PARP1 in the so-called trapped PARP1-DNA complexes actually represents the total PARP1 associated with the chromatin but not just the PARP1 that binds to DNA.

2) To conduct "trapped PARP1-DNA complexes" protein fractionation, the current protocols need to make dramatic treatments to obtain the chromatin, which might lead to the uncontrollable loss of PARP1. Such uncontrollability has been evidenced by the big differences in the data appearing in the previous reports.

3) Moreover, the measurements of the cell viability, IC_{50}, require no addition of MMS but the measurements of trapping require the addition of MMS.

All these reasons might be responsible for a weak correlation between PARP1-DNA trapping by PARPi and their cytotoxicity. Therefore, the trapping model might be correct if it could accurately detect the PARP1 that only binds DNA.

Q3.2.The authors make a somewhat discrepant statement elsewhere (lines 285-87), where they suggest that PARPi kill BRCA-mutant cells via inhibition of BER. How does this relate to cytotoxicity induced by DNA trapping of PARP?.

Response: The anticancer mechanism of PARPi for cancer therapy has not been fully understood yet (PMID: 27416328). Initially, PARPi were believed to impair BER-mediated single-strand break repair (SSB), and the accumulated SSBs are converted to DSBs, which results in the cell death of HR-deficient cells via "synthetic lethality". However, this mechanism can not fully explain the poor correlation between the PARP inhibition capacity of the inhibitors and their cytotoxic effects. Then, PARP1 trapping was proposed as another possible mechanism for the effects of PARPi. Previous studies have shown that PARPi promotes the toxic binding of PARP1 on BER intermediates and impedes BER repair (PMID: 34102106), suggesting an interaction between different mechanisms.

Q3.3. (2) Figure 2c/2d: The authors claim that thioparib is effective in killing BRCA1/2-mutant cancer cells that have acquired resistance via somatic reversion mutations. This is potentially an important claim - but it is difficult to reconcile with the published evidence that somatic reversion mutants in BRCA1/2 at least partially restore DNA repair by HR

. Does HR restoration occur in the revertant cell lines used in this study? If so, by what mechanism does thioparib kill the revertant cells? These results seem preliminary, and need further experimental analysis. Similar studies to those carried out in Fig 4 would provide some insight.

On this point, the authors later show (Fig 4g, h and Table S6) that PARP1 depletion decreases the sensitivity of revertant cells to thioparib. This finding is puzzling if the revertants have restored HR capacity, and suggest that thioparib may have unrelated mechanisms of action.

Response: Thanks for the insightful comments and suggestions. Our previous work has reported the molecular mechanisms of acquired PARPi-resistant in the revertant cell lines (PMID: 28414200; 29274141; 33042619). As suggested by the reviewer, we further evaluated whether these revertant cells had an increased proficiency in HR repair. We quantified the number of RAD51 foci after irradiation in both parent and resistant cells. As shown in **new Fig. EV2**, BRCA1/2 null cells are deficient in the ability to form RAD51 foci after DNA damage. However, all revertant cells had partially acquired the ability to form RAD51 foci after exposure to X-rays.

As the reviewer notes, thioparib is effective in killing BRCA1/2-mutant cells, including those acquired resistant to olaparib. We speculate that thioparib effectively kills the resistant cells by blocking HR activity. As our response to reviewer 2, we have used PARP1-depletion cells to gain additional insights. The data show that KO of PARP1 only partially (~50%) rescued the effect of thioparib on HR activity, but

almost completely reversed the effect of other PARPi (**new Fig. 5H**). Our findings suggest that PARP1 inhibition is at least one of the mechanisms that explain how thioparib inhibits HR repair. We speculate that thioparib may have unknown mechanisms differing from that of other PARPi, and the unknown factors contribute to HR repair suppression. We hope to address this specific issue in the future.

Q3. (3) Figure 2e: Similar concerns apply to the authors' work on the primary PARP-resistant PDX model BR-00-28, generated from a BRCA1-deficient human breast cancer. Do these cells retain HR capacity to any degree? If so, by what mechanism does thioparib work here? Again, the results shown seem preliminary and need further study.

Response: BR-05-0028 samples used for PDX development were derived from a breast cancer patient with BRCA1-mutated (exon10:c.C1630T:p.Q544X), and the tumor tissues were transplanted and grown in nude mice subcutaneously. However, it is difficult for us to analyze the HR capacity of the tumor tissues. As mentioned above, the remaining mechanisms of how thioparib overcomes PARPi-resistance is a focus of our experiments in the future.

21st Nov 2022

Dear Dr. Wang,

Thank you for the submission of your manuscript to EMBO Molecular Medicine, and please accept my apologies for the delay in getting back to you as I one referee needed more time to provide his/her report. We have now received feedback from the three referees who originally reviewed your manuscript. As you will see below, while referees #2 and #3 are supportive of publication, referee #1 still raises several concerns. After discussion with my colleagues, we think these concerns can be addressed in a minor round of revisions, and we would therefore invite you to further revise the manuscript to address all referee #1's concerns.

Moreover, please also address the following editorial points:

1/ Manuscript text:
- Please address the queries from our data editor in the Data edited manuscript file (figure legends). Please use this file for any further modification. Please remove the highlighted text and only keep in track changes mode any new modification.
- Material and methods:
o Synthesis of thioparib and Cpd-391: please make sure that the information from patent CN201510818057.7 is easily accessible to all (I only found a version in Chinese).
o Please move the supplementary Materials and Methods currently in the Appendix to the main manuscript file.
o Animal experiments: Please indicate the housing and husbandry conditions.
- Please remove the list of appendix materials from the manuscript file.
- Please merge the funding section with the acknowledgements. Please make sure that the information provided in the manuscript is the same as what is provided in the submission system.
- Author contributions: CRediT has replaced the traditional author contributions section because it offers a systematic machine-readable author contributions format that allows for more effective research assessment. Please remove the Authors Contributions from the manuscript and use the free text boxes beneath each contributing author's name in our system to add specific details on the author's contribution. More information is available in our guide to authors.
- We note that there are currently a total of 4 co-corresponding authors (3 in the submission system). Is that correct? Do you confirm equal contribution of these 4 people, able to take full responsibility for the paper and its content? While there is no limit per se to the number of co-corresponding authors, 3 is rare, 4 even more so, and may not reflect as intended to the community.
- Please rename the "Disclosure statement and competing interests" section. We updated our journal's competing interests policy in January 2022 and request authors to consider both actual and perceived competing interests. Please review the policy https://www.embopress.org/competing-interests and update your competing interests if necessary.
- Please rename and place the Data Availability section after the Materials and Methods.
- Please move the Figure legends after the References and add a heading "Expanded View Figure Legends".

2/ Figures:
- Please make sure that all figures and figure panels are referenced in the manuscript text. Callouts for figures EV2A and EV2B are currently missing.
- Please change the name of Appendix Data File 1 to "Dataset EV1", and add a legend in a separate tab. Please remove it from the Appendix table of content.

3/ At EMBO Press we encourage authors to provide source data for the main and EV figures. Numerical data should be provided as individual .xls or .csv files (including a tab describing the data). For blots or microscopy, uncropped images should be submitted (using a zip archive if multiple images need to be supplied for one panel). Additional information on source data and instruction on how to label the files are available at
.

4/ Checklist:
Please fill in the Data Availability section.

5/ Thank you for providing the Pape Explained. Please include it in the main manuscript file.

6/ Thank you for providing a synopsis. I slightly edited the text, please let me know if you agree with the following, or amend as you see fit:

PARP inhibitors (PARPi) resistance is ubiquitous in the clinic. A newly discovered pan-PARP inhibitor, thioparib, is highly effective against olaparib-resistant cancer models, which suggests that therapeutic vulnerabilities still exist in PARPi-resistant tumors.

- Thioparib is a novel, potent, and orally bioavailable pan-PARP inhibitor.

- Thioparib overcomes primary and acquired olaparib resistance in vitro and in vivo.
- Thioparib suppresses HR-mediated DNA repair.
- Thioparib induces a robust type I interferon response.

Thank you for providing a nice synopsis image. Please resize it as a PNG/JPEG/TIFF file 550 px wide x 300-600 px high, and make sure that the text remains legible.

7/ As part of the EMBO Publications transparent editorial process initiative (see our Editorial at http://embomolmed.embopress.org/content/2/9/329), EMBO Molecular Medicine will publish online a Review Process File (RPF) to accompany accepted manuscripts.
This file will be published in conjunction with your paper and will include the anonymous referee reports, your point-by-point response and all pertinent correspondence relating to the manuscript. Let us know whether you agree with the publication of the RPF and as here, if you want to remove or not any figures from it prior to publication.
Please note that the Authors checklist will be published at the end of the RPF.

I look forward to receiving your revised manuscript.

Yours sincerely,

Lise Roth

Lise Roth, PhD
Senior Editor
EMBO Molecular Medicine

***** Reviewer's comments *****

Referee #1 (Comments on Novelty/Model System for Author):

The authors have performed additional experiments that indicate (i) immunostimulatory effects of thioparib through PARP7 inhibition BUT ALSO (ii) adverse effects of concomitant PARP1/2 inhibition, clearly observed in the newly added immunocompetent mouse model. Therefore, the findings described in this manuscript remain of low medical impact because of pan-PARP inhibition causing conflicting in vivo effects.

Referee #1 (Remarks for Author):

Q1.1.

How was the inhibition of p38 measured? The table in Fig. S2K states inhibition rates based on one thioparib concentration (1 uM). Please clarify how this experiment was conducted and how inhibition rates were determined.

Based on the new immunocompetent mouse model data in Fig. 6, pleiotropic effects of thioparib that are mediated through PARP1 (such as activation of p38) are clearly counterproductive because PARP1 KO mice show a much better response to thioparib treatment due to PARP7 inhibition. Therefore, using a specific PARP7 inhibitor, rather than a pan-inhibitor such as thioparib, would be relevant for clinical applications. This must be highlighted in the manuscript, including the abstract.

Q1.2.

Yes, the differences are visible now.

Q1.3.

Please provide also CD4, CD8 and CD45 expression levels for conditions in panel 6I.

Q1.5.

The added sentence referencing Rudolf et al 2022 clashes with the preceding one citing Murai et al 2014, as Rudolf et al show that PARP1/2 inhibitors don't have similar enzymatic inhibiting activities.

Q1.6.

The way the text is written is still very confusing and can lead to misinterpretation. It is important to explain the differences between PARP1-DNA binding (without PARP1 activity, allows analysis of allosteric effects) and PARP1 trapping (when PARP1 is active in the presence of NAD). When you mention PARP1-DNA binding in the text, you actually refer to PARP1 trapping. Please rephrase this section for clarity.

Q1.10.

It currently says: 'similar influences', which is again vague. I suggest saying 'showed similar inhibitory effects on PARP1 and PARP2 activities'

Q1.15.
It is necessary to comment on this in the manuscript text as Fig. 6D and F still look very different considering STAT1 levels in PARP1 KO.

Q1.16.

Please modify Fig. 6E directly by normalizing everything against Parent untreated. Otherwise the figure gives a false impression of very low IFNB1 and CXCL10 levels in PARP7 KO. They are actually as high as in PARP1 KO, again showing that PARP7 KO or inhibition alone is sufficient to induce IFNB1 response. Data in EV4J should be normalized the same way.

Q1.19.

Thank you for performing these additional experiments, which indeed point at PARP7 inhibition as the major source of immunostimulatory effects. However, it's important to also indicate in the manuscript text that PARP1 inhibition by thioparib contributes to cGAS/STING activation, most likely due to accumulation of cytosolic dsDNA caused by replication stress-induced mitotic defects.

Based on the immunocompetent mouse model, pan-inhibitory effects of thioparib coming from PARP1 inhibition are counterproductive, as already noted above.

Q1.20.

The blots clearly show that pSTAT1 is increased under basal conditions in PARP1 KO. The quantification blot shows normalized data (1 being basal condition for both WT and PARP1 KO cells), which means that WT and KO cannot be compared under basal conditions.

The simplest explanation here would be that cGAS/STING is activated in PARP1 KO due to DNA damage-induced accumulation of cytosolic dsDNA (see my comment above).

All combined, PARP1 inhibition through thioparib or PARP1 KO induce DNA damage and activate cGAS/STING. As a result, thioparib and RBN-2397 (specific PARP7 inhibitor) have a stronger effect on pSTAT1 in PARP1 KO.

Referee #2 (Remarks for Author):

The manuscript is suitable for publication to EMBO Molecular Medicine.

Referee #3 (Comments on Novelty/Model System for Author):

The authors have revised their manuscript to address my prior concerns regarding technical quality and adequacy of their model systems. Their identification of a new PARP inhibitor with a wider spectrum of activity beyond PARP1, potential to overcome PARP1i resistance, and potential to activate immune signaling, is novel and of potentially high medical impact.

Referee #3 (Remarks for Author):

The authors have satisfactorily addressed my original comments. The revised paper demonstrates that Thioparib is a PARP inhibitor with a wider spectrum of activity beyond PARP1, with potential value in overcoming PARP1i resistance, and potential to activate immune signaling. The revised paper now extends confirmation of immune activation by Thioparib using an in vivo model. The authors also provide new evidence that Thioparib's mechanism of action on DNA repair is both via PARP1 inhibition as well as other pathways to be defined, and the mechanism of immune activation is through PARP7-STING. This level of mechanistic insight, albeit not fully complete, seems appropriate to the scope of the paper.

Point-by-point Responses to the Referees' Comments

We thank the referees for their time and efforts to re-review our manuscript. Because Referees #2 and #3 had no new concerns about our manuscript, here we only responded to Referee #1's comments as follows.

Referee #1's Comments:

Referee #1 (Comments on Novelty/Model System for Author):

The authors have performed additional experiments that indicate (i) immunostimulatory effects of thioparib through PARP7 inhibition BUT ALSO (ii) adverse effects of concomitant PARP1/2 inhibition, clearly observed in the newly added immunocompetent mouse model. Therefore, the findings described in this manuscript remain of low medical impact because of pan-PARP inhibition causing conflicting in vivo effects.

Response: We understood the referee's concern about pan-PARP inhibition causing conflicting *in vivo* effects between immunostimulatory effects due to PARP7 inhibition and adverse effects of concomitant PARP1/2 inhibition. However, as shown in extensive clinical trials and clinical uses of other well-known PARP1/2 inhibitors such as olaparib and talazoparib, adverse effects of PARP1/2 inhibition are controllable through proper dosage regimens. Actually, thioparib concomitantly inhibits PARP1/2/7, conferring it a wider spectrum of activity beyond PARP1, potential value in overcoming PARP1i resistance, and potential to activate immune signaling, as pointed out in Referee #3's remarks and as discussed in the section of Discussion in the manuscript.

Referee #1 (Remarks for Author):

Q1.1. How was the inhibition of p38 measured? The table in Fig. S2K states inhibition rates based on one thioparib concentration (1 µM). Please clarify how this experiment was conducted and how inhibition rates were determined.

Based on the new immunocompetent mouse model data in Fig. 6, pleiotropic effects of thioparib that are mediated through PARP1 (such as activation of p38) are clearly counterproductive because PARP1 KO mice show a much better response to thioparib treatment due to PARP7 inhibition. Therefore, using a specific PARP7 inhibitor, rather than a pan-inhibitor such as thioparib, would be relevant for clinical applications. This must be highlighted in the manuscript, including the abstract.

Response: As suggested by the referee, we have added more details about the "p38 enzyme inhibition assay" in the Materials and Methods (Lines 750-753, Page 33).

Indeed, immunocompetent MC38 PARP1#KO mice show a better response to thioparib treatment due to PARP7 inhibition, suggesting PARP7 is an attractive target

for immunotherapy. However, pan-PARP inhibitor thioparib has been demonstrated to be effective against not only some PARP7i-sensitive tumors but also PARP1i-resistant cancers. We have added more discussions in the revised manuscript (Lines 663-668, Pages 29-30).

Q1.2. Yes, the differences are visible now.

Thank you!

Q1.3. Please provide also CD4, CD8 and CD45 expression levels for conditions in panel 6I.

Response: We have included the data of CD4, CD8 and CD45 expression levels for conditions in panel 6I as suggested (new Appendix Fig. S3) (Lines 513-515, Page 23).

Q1.5. The added sentence referencing Rudolf et al 2022 clashes with the preceding one citing Murai et al 2014, as Rudolf et al show that PARP1/2 inhibitors don't have similar enzymatic inhibiting activities.

Response: The statement has been revised (Lines 103-105, Page 5).

Q1.6. The way the text is written is still very confusing and can lead to misinterpretation. It is important to explain the differences between PARP1-DNA binding (without PARP1 activity, allows analysis of allosteric effects) and PARP1 trapping (when PARP1 is active in the presence of NAD). When you mention PARP1-DNA binding in the text, you actually refer to PARP1 trapping. Please rephrase this section for clarity.

Response: We agree with the referee. This section has been revised for clarity (Lines 109-119, Pages 5-6 and Lines 154-156, Page 7).

Q1.10. It currently says: 'similar influences', which is again vague. I suggest saying 'showed similar inhibitory effects on PARP1 and PARP2 activities'?

Response: Thank you. We have revised it as suggested (Line 173, Page 8).

Q1.15. It is necessary to comment on this in the manuscript text as Fig. 6D and F still look very different considering STAT1 levels in PARP1 KO.

Response: Based on the referee's comment, we have described in the result part that basal STAT1 levels were upregulated in the PARP1 KO1 clone (Fig.6D), but not in the KO2 clone (new Appendix Fig.S2E) (Lines 446-449, Page 20). In the current version of Fig.6F, no PARP1 KO cells were included.

Q1.16. Please modify Fig. 6E directly by normalizing everything against Parent untreated. Otherwise the figure gives a false impression of very low IFNB1 and CXCL10 levels in PARP7 KO. They are actually as high as in PARP1 KO, again

showing that PARP7 KO or inhibition alone is sufficient to induce IFNB1 response. Data in EV4J should be normalized the same way.

Response: We have included the absolute mRNA levels of *IFNB1* and *CXCL10* by normalizing against parent untreated in Fig.6E and EV4J.

Q1.19. Thank you for performing these additional experiments, which indeed point at PARP7 inhibition as the major source of immunostimulatory effects. However, it's important to also indicate in the manuscript text that PARP1 inhibition by thioparib contributes to cGAS/STING activation, most likely due to accumulation of cytosolic dsDNA caused by replication stress-induced mitotic defects.

Based on the immunocompetent mouse model, pan-inhibitory effects of thioparib coming from PARP1 inhibition are counterproductive, as already noted above.

Response: We thank the reviewer for the insightful comments. A few more discussions on the contribution of PARP1 inhibition to cGAS/STING activation have been added in the revised manuscript (Lines 497-501, Page 22).

As in responses to Comments on Novelty/Model System for Author and Q1.1, the pan-PARP inhibitor thioparib has been demonstrated to be effective against not only some PARP7i-sensitive tumors but also PARP1i-resistant cancers, which is of potentially high medical impact.

Q1.20. The blots clearly show that pSTAT1 is increased under basal conditions in PARP1 KO. The quantification blot shows normalized data (1 being basal condition for both WT and PARP1 KO cells), which means that WT and KO cannot be compared under basal conditions.

The simplest explanation here would be that cGAS/STING is activated in PARP1 KO due to DNA damage-induced accumulation of cytosolic dsDNA (see my comment above).

All combined, PARP1 inhibition through thioparib or PARP1 KO induce DNA damage and activate cGAS/STING. As a result, thioparib and RBN-2397 (specific PARP7 inhibitor) have a stronger effect on pSTAT1 in PARP1 KO.

Response: Thanks. The referee's comments and suggestions have been integrated into our revised manuscript. A few more discussions on PARP1-cGAS/STING signaling and specific PARP7 inhibitor have been added in the revised manuscript (Lines 497-501, Page 22 and Lines 663-668, Pages 29-30).

16th Dec 2022

Dear Dr. Wang,

Thank you for the submission of your revised manuscript to EMBO Molecular Medicine. We have now received feedback from referee #1, who is supportive of publication pending minor revisions. Therefore, please address this referee's concerns in the text.
Please accept all previous changes, and only keep in track changes mode the new modifications.

I look forward to receiving your revised manuscript.

Yours sincerely,

Lise Roth

Lise Roth, PhD

Senior Editor

EMBO Molecular Medicine

.

***** Reviewer's comments *****

Referee #1 (Comments on Novelty/Model System for Author):

My main concern is how the data is interpreted and presented to the reader. It should be clearly stated in the Abstract that the anti-tumor effect of thioparib is stronger in PARP1 KO mice, as indicated under my first comment to the authors.

Referee #1 (Remarks for Author):

1) As previously explained, the abstract must indicate that thioparib is more efficient in PARP1 KO mouse tumor models. Otherwise the readers will get the wrong impression that inhibition of both PARP1 and PARP7 by thioparib is beneficial for cancer therapy. I suggest adding the following statement after 'and resulted in tumor growth retardation in an immunocompetent mouse model.'
However, the inhibitory effect of thioparib on tumor growth was more pronounced in PARP1 knock-out mice, suggesting that a specific PARP7 inhibitor, rather than a pan-inhibitor such as thioparib, would be more relevant for clinical applications.
2) Lines 671-673:
'However, pan-PARP inhibitor thioparib which acts on both DNA damage response and
antitumor immunity, is highly effective against not only some PARP7i-sensitive tumors but also
PARP1i-resistant cancers.'
You cannot make this statement because you haven't tested PARP1i-resistant cancer cell lines in a mouse model. You can only state:
'Based on our in vitro data, thioparib is potent against PARP1i-resistant cancer cell lines, which remains to be tested in a mouse model.'
3) The section on PARP trapping is still confusing. I would rewrite as follows:
In addition to catalytic inhibition, PARPi exert their cytotoxicity by preventing PARP1 autoPARylation and trapping it on damaged DNA. Compared with other PARPi, talazoparib is a 3- to 8-fold more potent PARP1 inhibitor in vitro (Shen et al, 2013), but it exhibits 100-fold greater potency at trapping PARP-DNA complexes compared to olaparib and rucaparib in cells (Murai et al, 2014). In the "PARP1 trapping" model, trapping is defined as chromatin-bound PARP1 in methyl methanesulfonate (MMS)-treated cells (Helleday, 2011; Ström et al, 2012). By measuring changes in PARP1-DNA binding (i.e., changes in fluorescence

anisotropy values, termed ΔFA values) in the presence of NAD+, we showed that the differences in the cytotoxicity of PARPi originate from increased PARP1-DNA binding due to autoPARylation inhibition of PARP1 on DNA (Chen et al, 2019). These results provide evidence that PARP-DNA binding activity is an important factor for PARPi-induced cytotoxicity. Furthermore, recent studies suggest that PARP trapping is primarily due to the inhibition of the activity of PARP1 and that the basis for the high potency of talazoparib lies in its extensive interactions with the active sites of PARP1 (Rudolph et al, 2022). Thus, we propose that novel PARPi should be screened and assessed by both PARP enzymatic and PARP1-DNA binding activities.
4) The added statement to address the contribution of PARP1 to IFNB1 induction is misplaced:
'The above data indicated that thioparib has stronger effects on p-STAT1 and IFNB1/CXCL10 gene expression in PARP1 KO cells as compared to parental cells (Fig. 6D and 6E). We thus proposed that PARP1 inhibition with thioparib treatment leads to replication-associated DNA damage, triggers the release of cytosolic dsDNA, and subsequently activates the cGAS/STING signaling.'
Thioparib cannot act on PARP1 in PARP1 KO cells, so PARP1 inhibition by thioparib cannot explain increased p-STAT1 and IFNB1/CXCL10. PARP1 inhibition by thioparib may explain why cGAS/STING signaling is induced in PARP7 KO. The fact that cGAS/STING is much more induced in PARP1 KO treated with thioparib compared to WT again shows that concomitant inhibition of PARP1 is counterproductive.

Point-by-point Responses to Referee #1's Comments

We thank the referee for the time and efforts to re-review our manuscript. Sincere thanks also to Referee #1 for the suggestions on data interpreting and presenting.

Referee #1's Comments:

Referee #1 (Comments on Novelty/Model System for Author):

My main concern is how the data is interpreted and presented to the reader. It should be clearly stated in the Abstract that the anti-tumor effect of thioparib is stronger in PARP1 KO mice, as indicated under my first comment to the authors.

Response: Sincere thanks again to Referee #1 for the time and efforts spent on our manuscript. We agree with the referee and have stated in the Abstract that the anti-tumor effect of thioparib is stronger in PARP1 KO mice. Other concerns on data interpretation and presentation were also addressed as the referee suggested.

Referee #1 (Remarks for Author):

1) As previously explained, the abstract must indicate that thioparib is more efficient in PARP1 KO mouse tumor models. Otherwise the readers will get the wrong impression that inhibition of both PARP1 and PARP7 by thioparib is beneficial for cancer therapy. I suggest adding the following statement after 'and resulted in tumor growth retardation in an immunocompetent mouse model.'

However, the inhibitory effect of thioparib on tumor growth was more pronounced in PARP1 knock-out mice, suggesting that a specific PARP7 inhibitor, rather than a pan-inhibitor such as thioparib, would be more relevant for clinical applications.

Response: We have revised our manuscript as suggested (Line 34-37, Page 2).

2) Lines 671-673:

'However, pan-PARP inhibitor thioparib which acts on both DNA damage response and antitumor immunity, is highly effective against not only some PARP7i-sensitive tumors but also PARP1i-resistant cancers. '

You cannot make this statement because you haven't tested PARP1i-resistant cancer cell lines in a mouse model. You can only state:

'Based on our in vitro data, thioparib is potent against PARP1i-resistant cancer cell lines, which remains to be tested in a mouse model.'

Response: Thank you. We have revised our manuscript as suggested and slightly edited the text (Lines 663-666, Pages 29-30).

3) The section on PARP trapping is still confusing. I would rewrite as follows:

In addition to catalytic inhibition, PARPi exert their cytotoxicity by preventing PARP1 autoPARylation and trapping it on damaged DNA. Compared with other PARPi, talazoparib is a 3- to 8-fold more potent PARP1 inhibitor in vitro (Shen et al, 2013), but it exhibits 100-fold greater potency at trapping PARP-DNA complexes compared to olaparib and rucaparib in cells (Murai et al, 2014). In the "PARP1 trapping" model, trapping is defined as chromatin-bound PARP1 in methyl methanesulfonate (MMS)-treated cells (Helleday, 2011; Ström et al, 2012). By measuring changes in PARP1-DNA binding (i.e., changes in fluorescence anisotropy values, termed ΔFA values) in the presence of NAD+, we showed that the differences in the cytotoxicity of PARPi originate from increased PARP1-DNA binding due to autoPARylation inhibition of PARP1 on DNA (Chen et al, 2019). These results provide evidence that PARP-DNA binding activity is an important factor for PARPi-induced cytotoxicity. Furthermore, recent studies suggest that PARP trapping is primarily due to the inhibition of the activity of PARP1 and that the basis for the high potency of talazoparib lies in its extensive interactions with the active sites of PARP1 (Rudolph et al, 2022). Thus, we propose that novel PARPi should be screened and assessed by both PARP enzymatic and PARP1-DNA binding activities.

Response: Thank you. The statement has been revised as suggested (Lines 103-117, Pages 5-6).

4) The added statement to address the contribution of PARP1 to IFNB1 induction is misplaced:

'The above data indicated that thioparib has stronger effects on p-STAT1 and IFNB1/CXCL10 gene expression in PARP1 KO cells as compared to parental cells (Fig. 6D and 6E). We thus proposed that PARP1 inhibition with thioparib treatment leads to replication-associated DNA damage, triggers the release of cytosolic dsDNA, and subsequently activates the cGAS/STING signaling.'

Thioparib cannot act on PARP1 in PARP1 KO cells, so PARP1 inhibition by thioparib cannot explain increased p-STAT1 and IFNB1/CXCL10. PARP1 inhibition by thioparib may explain why cGAS/STING signaling is induced in PARP7 KO. The fact that cGAS/STING is much more induced in PARP1 KO treated with thioparib compared to WT again shows that concomitant inhibition of PARP1 is counterproductive.

Response: We thank the referee for the insightful comments. We have edited the statement addressing the contribution of PARP1 to IFNB1 induction in the sections of Results and Discussion (Lines 441-444, Page 20, and Lines 653-657, Page 29).

21st Dec 2022

Dear Dr. Wang,

Thank you for submitting your revised files. I am pleased to inform you that your manuscript is accepted for publication and is now being sent to our publisher to be included in the next available issue of EMBO Molecular Medicine.

Please note that I have replaced the text of the Data Availability section by: "This study includes no data deposited in external repositories." Indeed, this section is intended for new primary large datasets that must be deposited in external public repository. Please contact us immediately if you do not agree with this change.

Please also note that an ORCID identified is still missing for Ao Zhang. All corresponding authors must have an ORCID identifier before online publication.

We would like to remind you that as part of the EMBO Publications transparent editorial process initiative, EMBO Molecular Medicine will publish a Review Process File online to accompany accepted manuscripts. If you do NOT want the file to be published or would like to exclude figures, please immediately inform the editorial office via e-mail.

Congratulations on your interesting work,

With kind regards,

Lise Roth

Lise Roth, Ph.D
Senior Editor
EMBO Molecular Medicine

*** *** *** IMPORTANT INFORMATION *** *** ***

SPEED OF PUBLICATION
The journal aims for rapid publication of papers, using using the advance online publication "Early View" to expedite the process: A properly copy-edited and formatted version will be published as "Early View" after the proofs have been corrected. Please help the Editors and publisher avoid delays by providing e-mail address(es), telephone and fax numbers at which author(s) can be contacted.

Should you be planning a Press Release on your article, please get in contact with embomolmed@wiley.com as early as possible, in order to coordinate publication and release dates.

LICENSE AND PAYMENT:

All articles published in EMBO Molecular Medicine are fully open access: immediately and freely available to read, download and share.

EMBO Molecular Medicine charges an article processing charge (APC) to cover the publication costs. You, as the corresponding author for this manuscript, should have already received a quote with the article processing fee separately. Please let us know in case this quote has not been received.

Once your article is at Wiley for editorial production you will receive an email from Wiley's Author Services system, which will ask you to log in and will present you with the publication license form for completion. Within the same system the publication fee can be paid by credit card, an invoice, pro forma invoice or purchase order can be requested.

Payment of the publication charge and the signed Open Access Agreement form must be received before the article can be published online.

PROOFS

You will receive the proofs by e-mail approximately 2 weeks after all relevant files have been sent o our Production Office. Please return them within 48 hours and if there should be any problems, please contact the production office at embopressproduction@wiley.com.

Please inform us if there is likely to be any difficulty in reaching you at the above address at that time. Failure to meet our deadlines may result in a delay of publication.

All further communications concerning your paper proofs should quote reference number EMM-2022-16235-V4 and be directed to the production office at embopressproduction@wiley.com.

Thank you,

Lise Roth, Ph.D
Senior Editor
EMBO Molecular Medicine

EMBO Press Author Checklist

Corresponding Author Name: Limin-Wang	
Journal Submitted to: EMBO Molecular Medicine	
Manuscript Number: EMM-2022-16235	

USEFUL LINKS FOR COMPLETING THIS FORM
[The EMBO Journal - Author Guidelines]
[EMBO Reports - Author Guidelines]
[Molecular Systems Biology - Author Guidelines]
[EMBO Molecular Medicine - Author Guidelines]

Reporting Checklist for Life Science Articles (updated January 2022)
This checklist is adapted from Materials Design Analysis Reporting (MDAR) Checklist for Authors. MDAR establishes a minimum set of requirements in transparent reporting in the life sciences (see Statement of Task: 10.31222/osf.io/9sm4x). Please follow the journal's guidelines in preparing your manuscript.
Please note that a copy of this checklist will be published alongside your article.

Abridged guidelines for figures
1. Data
The data shown in figures should satisfy the following conditions:
- the data were obtained and processed according to the field's best practice and are presented to reflect the results of the experiments in an accurate and unbiased manner.
- ideally, figure panels should include only measurements that are directly comparable to each other and obtained with the same assay.
- plots include clearly labeled error bars for independent experiments and sample sizes. Unless justified, error bars should not be shown for technical replicates.
- if n<5, the individual data points from each experiment should be plotted. Any statistical test employed should be justified.
- Source Data should be included to report the data underlying figures according to the guidelines set out in the authorship guidelines on Data Presentation.

2. Captions
Each figure caption should contain the following information, for each panel where they are relevant:
- a specification of the experimental system investigated (eg cell line, species name).
- the assay(s) and method(s) used to carry out the reported observations and measurements.
- an explicit mention of the biological and chemical entity(ies) that are being measured.
- an explicit mention of the biological and chemical entity(ies) that are altered/varied/perturbed in a controlled manner.
- the exact sample size (n) for each experimental group/condition, given as a number, not a range;
- a description of the sample collection allowing the reader to understand whether the samples represent technical or biological replicates (including how many animals, litters, cultures, etc.).
- a statement of how many times the experiment shown was independently replicated in the laboratory.
- definitions of statistical methods and measures:
  - common tests, such as t-test (please specify whether paired vs. unpaired), simple χ2 tests, Wilcoxon and Mann-Whitney tests, can be unambiguously identified by name only, but more complex techniques should be described in the methods section;
  - are tests one-sided or two-sided?
  - are there adjustments for multiple comparisons?
  - exact statistical test results, e.g., P values = x but not P values < x;
  - definition of 'center values' as median or average;
  - definition of error bars as s.d. or s.e.m.

Please complete ALL of the questions below.
Select "Not Applicable" only when the requested information is not relevant for your study.

Materials

Newly Created Materials	Information included in the manuscript?	In which section is the information available? (Reagents and Tools Table, Materials and Methods, Figures, Data Availability Section)
New materials and reagents need to be available; do any restrictions apply?	Yes	Materials and Methods: synthesis of thioparib and Cpd-391

Antibodies	Information included in the manuscript?	In which section is the information available? (Reagents and Tools Table, Materials and Methods, Figures, Data Availability Section)
For **antibodies** provide the following information:<br>- Commercial antibodies: RRID (if possible) or supplier name, catalogue number and or/clone number<br>- Non-commercial: RRID or citation	Yes	Appendix Materials and Methods: western blotting and RAD51 foci formation assay

DNA and RNA sequences	Information included in the manuscript?	In which section is the information available? (Reagents and Tools Table, Materials and Methods, Figures, Data Availability Section)
**Short novel DNA or RNA including primers, probes**: provide the sequences.	Yes	Appendix Materials and Methods: real-time quantitative PCR, RNA interference, and generation of gene knovkout cells using CRISPR/Cas9

Cell materials	Information included in the manuscript?	In which section is the information available? (Reagents and Tools Table, Materials and Methods, Figures, Data Availability Section)
**Cell lines:** Provide species information, strain. Provide accession number in repository **OR** supplier name, catalog number, clone number, and/**OR** RRID.	Yes	Appendix Materials and Methods: Cell lines
**Primary cultures:** Provide species, strain, sex of origin, genetic modification status.	Not Applicable	
Report if the cell lines were recently **authenticated** (e.g., by STR profiling) and tested for mycoplasma contamination.	Yes	Appendix Materials and Methods: Cell lines

Experimental animals	Information included in the manuscript?	In which section is the information available? (Reagents and Tools Table, Materials and Methods, Figures, Data Availability Section)
**Laboratory animals or Model organisms:** Provide species, strain, sex, age, genetic modification status. Provide accession number in repository **OR** supplier name, catalog number, clone number, **OR** RRID.	Yes	Materials and Methods: in vivo anticancer activity experiments
**Animal observed in or captured from the field:** Provide species, sex, and age where possible.	Not Applicable	
Please detail **housing and husbandry conditions**.	Yes	Materials and Methods: in vivo anticancer activity experiments

Plants and microbes	Information included in the manuscript?	In which section is the information available? (Reagents and Tools Table, Materials and Methods, Figures, Data Availability Section)
**Plants:** provide species and strain, ecotype and cultivar where relevant, unique accession number if available, and source (including location for collected wild specimens).	Not Applicable	
**Microbes:** provide species and strain, unique accession number if available, and source.	Not Applicable	

Human research participants	Information included in the manuscript?	In which section is the information available? (Reagents and Tools Table, Materials and Methods, Figures, Data Availability Section)
If collected and within the bounds of privacy constraints report on age, sex and gender or ethnicity for all study participants.	Not Applicable	

Core facilities	Information included in the manuscript?	In which section is the information available? (Reagents and Tools Table, Materials and Methods, Figures, Data Availability Section)
If your work benefited from core facilities, was their service mentioned in the acknowledgments section?	Not Applicable	

Design

Study protocol	Information included in the manuscript?	In which section is the information available? (Reagents and Tools Table, Materials and Methods, Figures, Data Availability Section)
If study protocol has been **pre-registered, provide DOI in the manuscript**. For clinical trials, provide the trial registration number **OR** cite DOI.	Not Applicable	
Report the **clinical trial registration number** (at ClinicalTrials.gov or equivalent), where applicable.	Not Applicable	

Laboratory protocol	Information included in the manuscript?	In which section is the information available? (Reagents and Tools Table, Materials and Methods, Figures, Data Availability Section)
Provide DOI OR other citation details if **external detailed step-by-step protocols** are available.	Not Applicable	

Experimental study design and statistics	Information included in the manuscript?	In which section is the information available? (Reagents and Tools Table, Materials and Methods, Figures, Data Availability Section)
Include a statement about **sample size** estimate even if no statistical methods were used.	Yes	Materials and Methods: study design and statistical analyses and respective figure legends
Were any steps taken to minimize the effects of subjective bias when allocating animals/samples to treatment (e.g. **randomization procedure**)? If yes, have they been described?	Yes	Materials and Methods: study design and statistical analyses and in vivo anticancer activity experiments
Include a statement about **blinding** even if no blinding was done.	Yes	Materials and Methods: study design and statistical analyses
Describe **inclusion/exclusion criteria** if samples or animals were excluded from the analysis. Were the criteria pre-established?<br><br>If sample or data points were omitted from analysis, report if this was due to attrition or intentional exclusion and provide justification.	Yes	Materials and Methods: study design and statistical analyses
For every figure, are **statistical tests** justified as appropriate? Do the data meet the assumptions of the tests (e.g., normal distribution)? Describe any methods used to assess it. Is there an estimate of variation within each group of data? Is the variance similar between the groups that are being statistically compared?	Yes	Materials and Methods: study design and statistical analyses and respective figure legends

Sample definition and in-laboratory replication	Information included in the manuscript?	In which section is the information available? (Reagents and Tools Table, Materials and Methods, Figures, Data Availability Section)
In the figure legends: state number of times the experiment was **replicated** in laboratory.	Yes	Materials and Methods: study design and statistical analyses and respective figure legends
In the figure legends: define whether data describe **technical or biological replicates**.	Yes	Materials and Methods: study design and statistical analyses and respective figure legends

Ethics

Ethics	Information included in the manuscript?	In which section is the information available? (Reagents and Tools Table, Materials and Methods, Figures, Data Availability Section)
Studies involving **human participants**: State details of **authority granting ethics approval** (IRB or equivalent committee(s), provide reference number for approval.	Not Applicable	
Studies involving **human participants**: Include a statement confirming that **informed consent** was obtained from all subjects and that the experiments conformed to the principles set out in the WMA Declaration of Helsinki and the Department of Health and Human Services Belmont Report.	Not Applicable	
Studies involving **human participants:** For publication of **patient photos**, include a statement confirming that consent to publish was obtained.	Not Applicable	
Studies involving experimental **animals**: State details of **authority granting ethics approval** (IRB or equivalent committee(s), provide reference number for approval. Include a statement of compliance with ethical regulations.	Yes	Materials and Methods: in vivo anticancer activity experiments
Studies involving **specimen and field samples:** State if relevant **permits** obtained, provide details of authority approving study; if none were required, explain why.	Not Applicable	

Dual Use Research of Concern (DURC)	Information included in the manuscript?	In which section is the information available? (Reagents and Tools Table, Materials and Methods, Figures, Data Availability Section)
Could your study fall under dual use research restrictions? Please check biosecurity documents and list of **select agents and toxins** (CDC): https://www.selectagents.gov/sat/list.htm	Not Applicable	
If you used a select agent, is the security level of the lab appropriate and reported in the manuscript?	Not Applicable	
If a study is subject to dual use research of concern regulations, is the name of the **authority granting approval and reference number** for the regulatory approval provided in the manuscript?	Not Applicable	

Reporting

The MDAR framework recommends adoption of discipline-specific guidelines, established and endorsed through community initiatives. Journals have their own policy about requiring specific guidelines and recommendations to complement MDAR.

Adherence to community standards	Information included in the manuscript?	In which section is the information available? (Reagents and Tools Table, Materials and Methods, Figures, Data Availability Section)
State if relevant guidelines or checklists (e.g., **ICMJE, MIBBI, ARRIVE, PRISMA**) have been followed or provided.	Not Applicable	
For **tumor marker prognostic studies**, we recommend that you follow the **REMARK** reporting guidelines (see link list at top right). See author guidelines, under 'Reporting Guidelines'. Please confirm you have followed these guidelines.	Not Applicable	
For **phase II and III randomized controlled trials**, please refer to the **CONSORT** flow diagram (see link list at top right) and submit the CONSORT checklist (see link list at top right) with your submission. See author guidelines, under 'Reporting Guidelines'. Please confirm you have submitted this list.	Not Applicable	

Data Availability

Data availability	Information included in the manuscript?	In which section is the information available? (Reagents and Tools Table, Materials and Methods, Figures, Data Availability Section)
Have **primary datasets** been deposited according to the journal's guidelines (see 'Data Deposition' section) and the respective accession numbers provided in the Data Availability Section?	Yes	Data Availability Section
Were **human clinical and genomic datasets** deposited in a public access-controlled repository in accordance to ethical obligations to the patients and to the applicable consent agreement?	Not Applicable	
Are **computational models** that are central and integral to a study available without restrictions in a machine-readable form? Were the relevant accession numbers or links provided?	Not Applicable	
If publicly available data were reused, provide the respective **data citations in the reference list**.	Not Applicable	
